# Genetic polymorphism and phylogenetic differentiation of the Huaxia Platinum System in three Chinese minority ethnicities

**DOI:** 10.1038/s41598-019-39794-y

**Published:** 2019-03-04

**Authors:** Jing Liu, Zheng Wang, Guanglin He, Mengge Wang, Yiping Hou

**Affiliations:** Institute of Forensic Medicine, West China School of Basic Medical Sciences & Forensic Medicine, Sichuan University, Chengdu, 610041 China

## Abstract

Short tandem repeats (STRs) with features of high polymorphism and abundant evolution information play a significant role in genetic applications such as human forensics, anthropology and population genetics. The Huaxia Platinum System was specifically exploited to allow coamplification of all markers in the expanded Combined DNA Index System and the Chinese National Database. Herein, in continuation of our previous studies, 493 unrelated individuals were firstly genotyped to investigate the efficacy of this novel system in three minority ethnicities of China (Hui, Tibetan and Uygur). Additionally, genetic relationships among our three investigated populations and other previously published populations were analyzed using pairwise genetic distances, multidimensional scaling (MDS), principal component analysis (PCA), cladogram and STRUCTURE. The combined match probabilities (CMP) for the Hui, Tibetan and Uygur groups were 1.6894 × 10^−27^, 6.1666 × 10^−27^ and 5.0655 × 10^−27^, respectively, and the combined powers of exclusion (CPE) were 0.999999999646627, 0.999999999304935 and 0.999999999433994. Population comparison analysis manifested that the Hui and Tibetan populations had genetic affinities with the Han, Yi and Korean populations, while the Uygur group had a close relationship with the Kazakh population. The aforementioned results suggested that the Huaxia Platinum System is a polymorphic and effective tool that is appropriate for personal identification and population genetics.

## Introduction

Short tandem repeats (STRs), also known as microsatellites, are DNA regions scattered throughout the genome with tandemly repeated short sequence motifs varying from 2–6 bases, and they have been the most commonly used genetic marker for human forensics^1,2^. In 1998, the Federal Bureau of Investigation (FBI) Laboratory selected 13 STR loci to constitute the Combined DNA Index System (CODIS), which has recently blossomed into the expanded CODIS containing an additional 7 STRs to increase the power of discrimination (PD)^3,4^. There are several commercial STR kits, such as the GlobalFiler PCR Amplification Kit (Thermo Fisher Scientific, USA)^5^ and PowerPlex Fusion Systems (Promega, USA)^6^, that contain 20 expanded CODIS loci (Supplementary Table S1) and have been developed and validated to promote data sharing. For the Chinese National Database (CND), the biggest DNA database in the worldwide, 20 STRs are required for uploading DNA profiles^7,8^. A total of 23 unique STRs exist between the expanded CODIS and the CND^9^. Recently, the Huaxia Platinum System (Thermo Fisher Scientific, USA) was specially developed to allow multiplex amplification and fluorescent detection of the 23 unique STRs as well as Amelogenin and Y-InDel (rs2032678) for sex determination. In light of our previous validation study, this assay was proven to be a highly polymorphic, informative, reliable and efficient tool for forensic investigation^9^.

China has 56 officially recognized ethnic groups and is the most populous country in the world. It is highly diverse, and its substructure is extremely complicated. The Hui, Tibetan and Uygur minority ethnicities are the most representative populations of West China. Among them, the Hui group is the largest and most widespread minority in the country, and most of them live in the Ningxia Hui Autonomous Region, Xinjiang Uygur Autonomous Region, and Qinghai and Gansu provinces. Their language belongs to Sinitic. The demographic history and previous population genetic studies indicated that the Hui minority originates from West and Central Asian-migrating Muslim groups and has a substantial admixture of the Han, Uygur and Mongolian groups^10,11^. The Tibetan population is one of the oldest ethnic groups in China, and their ancestor is the Diqiang, who are mainly distributed in the Tibet Autonomous Region, Qinghai and Western Sichuan. Tibetan belongs to the subbranch of the Tibeto-Burman of the Sino-Tibetan language, which is classified into Sinitic and Tibeto-Burman^12^. A previous study suggested that there are relatively small genetic diversities among Sino-Tibetan populations^13^. The Uygur minority is the fifth largest ethnic group in China and chiefly inhabits the Xinjiang Uygur Autonomous Region of Northwest China. Their language pertains to the Turkic subbranch of the Altaic language family. The evidence from a physical anthropology study showed that the Uygur people have an admixture between Europeans and East Asians, which makes it a research hotspot^14^.

In a continuation of our previous studies^9,15,16^, herein, we characterized the genetic diversity of the Huaxia Platinum System in the three aforementioned minority ethnicities (183 Ningxia Wuzhong Hui, 200 Sichuan Chengdu Tibetan and 110 Xinjiang Kumul Uygur individuals). Then, we evaluated population comparisons between the three studied populations and previously published populations based on 20 expanded CODIS loci. Furthermore, we conducted genetic structure analysis based on the genotype data of 23 STRs included in the Huaxia Platinum System from 11 populations. The details of relevant populations and their abbreviations are displayed in Supplementary Table S2.

## Results

### Forensic parameters of the Huaxia Platinum System

This study was implemented to obtain batches of genotype data (Supplementary Tables S3) from 23 STRs from the three populations (Ningxia Wuzhong Hui, Sichuan Chengdu Tibetan and Xinjiang Kumul Uygur). No significant deviation from Hardy-Weinberg equilibrium (HWE) was detected after Bonferroni correction was performed (p > 0.05/23 ≈ 0.0022), and no significant departure from linkage disequilibrium (LD) in the locus-by-locus pairwise comparison test was observed after Bonferroni correction was performed (p > 0.05/253 ≈ 0.0002) at any STR loci or in any ethnic group (Table 1 and Supplementary Tables S4–S6).

**Table 1:** Forensic parameters of 23 autosomal STR loci included in the Huaxia Platinum system in three Chinese populations (n_Hui_ = 183, n_Tibetan_ = 200, n_Uygur_ = 110, after Bonferroni correction p > 0.0022).

Locus	MP			PE			PIC			TPI			Ho			He			HWE-p		
	Hui	Tibetan	Uygur	Hui	Tibetan	Uygur	Hui	Tibetan	Uygur	Hui	Tibetan	Uygur	Hui	Tibetan	Uygur	Hui	Tibetan	Uygur	Hui	Tibetan	Uygur
CSF1PO	0.1244	0.1494	0.1570	0.4620	0.4680	0.5020	0.6770	0.6412	0.6420	1.7941	1.8182	1.9643	0.7213	0.7250	0.7455	0.7260	0.6897	0.7042	0.8859	0.2639	0.3430
D10S1248	0.1116	0.1108	0.0841	0.6154	0.5098	0.5494	0.7229	0.7072	0.7416	2.6143	2.0000	2.2000	0.8087	0.7550	0.7727	0.7637	0.7468	0.7759	0.1516	0.9060	0.9359
D12S391	0.0490	0.0706	0.0483	0.6676	0.5806	0.7219	0.8173	0.7772	0.8271	3.0500	2.3810	3.6667	0.8361	0.7950	0.8636	0.8396	0.8064	0.8490	0.8973	0.5952	0.6678
D13S317	0.0780	0.0636	0.0565	0.6154	0.6753	0.5990	0.7642	0.7914	0.8019	2.6143	3.1250	2.5000	0.8087	0.8400	0.8000	0.7964	0.8192	0.8277	0.6793	0.4389	0.4419
D16S539	0.0914	0.0802	0.0871	0.4620	0.5184	0.5334	0.7231	0.7506	0.7407	1.7941	2.0408	2.1154	0.7213	0.7600	0.7636	0.7630	0.7832	0.7780	0.1849	0.3205	0.7162
D18S51	0.0390	0.0483	0.0483	0.6782	0.6462	0.6682	0.8427	0.8193	0.8226	3.1552	2.8571	3.0556	0.8415	0.8250	0.8364	0.8604	0.8375	0.8457	0.4616	0.5953	0.7872
D19S433	0.0520	0.0585	0.0623	0.6570	0.6177	0.7219	0.8038	0.7974	0.7974	2.9516	2.6316	3.6667	0.8306	0.8050	0.8636	0.8279	0.8240	0.8229	0.9214	0.6322	0.2632
D1S1656	0.0544	0.0455	0.0498	0.6051	0.7047	0.7038	0.8017	0.8297	0.8203	2.5417	3.4483	3.4375	0.8033	0.8500	0.8546	0.8244	0.8489	0.8413	0.4533	0.8281	0.7039
D21S11	0.0633	0.0565	0.0770	0.6465	0.6177	0.5990	0.7992	0.7935	0.7749	2.8594	2.6316	2.5000	0.8251	0.8150	0.8000	0.8226	0.8189	0.8027	0.9286	0.7627	0.9424
D22S1045	0.0977	0.0888	0.1023	0.5074	0.4054	0.5176	0.7230	0.7285	0.7220	1.9891	1.5873	2.0370	0.7486	0.6850	0.7546	0.7663	0.7698	0.7641	0.5733	0.0047	0.8144
D2S1338	0.0361	0.0503	0.0435	0.7543	0.6559	0.7038	0.8502	0.8182	0.8465	4.1591	2.9412	3.4375	0.8798	0.8250	0.8546	0.8672	0.8393	0.8659	0.6162	0.6971	0.7273
D2S441	0.0944	0.0983	0.1012	0.5650	0.5098	0.4143	0.7348	0.7226	0.6962	2.2875	2.0000	1.6176	0.7814	0.7500	0.6909	0.7720	0.7641	0.7364	0.7614	0.6740	0.2788
D3S1358	0.1076	0.1371	0.1256	0.4890	0.4130	0.5494	0.7026	0.6556	0.6944	1.9063	1.6129	2.2000	0.7377	0.6900	0.7727	0.7475	0.7111	0.7442	0.7605	0.4907	0.4930
D5S818	0.0872	0.0968	0.1093	0.6465	0.3762	0.5334	0.7528	0.7115	0.7147	2.8594	1.4925	2.1154	0.8251	0.6600	0.7636	0.7880	0.7529	0.7587	0.2189	0.0044	0.9036
D6S1043	0.0344	0.0268	0.0398	0.7765	0.7246	0.5823	0.8553	0.8741	0.8432	4.5750	3.7037	2.3913	0.8907	0.8650	0.7909	0.8716	0.8867	0.8624	0.4385	0.3238	0.0295
D7S820	0.0957	0.0853	0.0688	0.5263	0.5358	0.4283	0.7305	0.7362	0.7753	2.0795	2.1277	1.6667	0.7596	0.7650	0.7000	0.7678	0.7711	0.8075	0.7908	0.8107	0.0042
D8S1179	0.0579	0.0595	0.0729	0.6360	0.7047	0.6682	0.8014	0.8007	0.7825	2.7727	3.4483	3.0556	0.8197	0.8550	0.8364	0.8264	0.8270	0.8109	0.8098	0.2870	0.4955
FGA	0.0408	0.0360	0.0393	0.6257	0.7346	0.7401	0.8388	0.8487	0.8495	2.6912	3.8462	3.9286	0.8142	0.8700	0.8727	0.8575	0.8653	0.8673	0.0936	0.8449	0.8665
Penta D	0.0578	0.0814	0.0777	0.5849	0.6367	0.6682	0.7972	0.7628	0.7669	2.4079	2.7778	3.0556	0.7924	0.8250	0.8364	0.8217	0.7947	0.7954	0.2995	0.3472	0.2863
Penta E	0.0160	0.0165	0.0240	0.8100	0.8982	0.7038	0.9171	0.9182	0.8985	5.3824	10.0000	3.4375	0.9071	0.9500	0.8546	0.9250	0.9263	0.9101	0.3582	0.1895	0.0419
TH01	0.1560	0.2105	0.1137	0.3335	0.3093	0.5176	0.6250	0.5606	0.7012	1.3657	1.2987	2.0370	0.6339	0.6200	0.7546	0.6661	0.6198	0.7432	0.3559	0.9186	0.7856
TPOX	0.1929	0.2449	0.1909	0.4190	0.2403	0.4283	0.5866	0.5100	0.5948	1.6339	1.1236	1.6667	0.6940	0.5550	0.7000	0.6475	0.5746	0.6585	0.1879	0.5753	0.3581
vWA	0.0735	0.0728	0.0714	0.6154	0.6272	0.5494	0.7692	0.7717	0.7679	2.6143	2.7027	2.2000	0.8087	0.8100	0.7727	0.8017	0.8036	0.7975	0.8121	0.6896	0.5185

MP: matching probability; PE: power of exclusion; PIC: polymorphism information content; TPI: typical paternity index; Ho: observed heterozygosity; He: expected heterozygosity; HWE-p: the p value of the Hardy-Weinberg equilibrium test.

The allele frequencies of 23 autosomal STR loci for the three populations are given in Supplementary Tables S7–S9. The forensic informative metrics include the match probability (MP), power of exclusion (PE), polymorphism information content (PIC), typical paternity index (TPI), observed heterozygosity (Ho) and expected heterozygosity (He), which are listed in Table 1. The Ho ranged from 0.5550 (TPOX) to 0.9500 (Penta E) in the Sichuan Chengdu Tibetan (SCT) population, with an average value of 0.7780, which was lower than the average values of the Xinjiang Kumul Uygur (XKU, 0.7937) and Ningxia Wuzhong Hui (NWH, 0.7952) ethnic groups. In the three populations, Penta E had the highest discrimination power and the MP values were 0.0160 (NWH), 0.0165 (SCT) and 0.0240 (XKU). TPOX had the lowest discrimination power and the MP values were 0.1929 (NWH), 0.2449 (SCT) and 0.1909 (XKU). The combined match probabilities (CMP) for the NWH, SCT and XKU groups were 1.6894 × 10^−27^, 6.1666 × 10^−27^ and 5.0655 × 10^−27^, respectively, and the combined powers of exclusion (CPE) were 0.999999999646627, 0.999999999304935 and 0.999999999433994.

### Interpopulation genetic distances

We performed locus-by-locus pairwise comparisons (F_st_) and calculated Nei’s standard genetic distances (R_st_) between our three studied populations and 47 previously published worldwide populations (Asian^9,15–29^, North American^30–32^, European^33–36^, Oceanian^37^, South American^38–41^ and South African^42^ populations) based on allele frequencies of 20 expanded CODIS loci to infer interpopulation similarity and differentiation (Supplementary Tables S10–S13 and Fig. S1). The locus-by-locus F_st_ and corresponding p values showed no significant genetic difference between the NWH group and Han, Tibetan, Yi and Korean populations at all loci; no significant genetic difference between the SCT population and Yi and Tibetan ethnic groups at all loci; and no significant genetic difference between the XKU ethnic group and the Xinjiang Uygur-3 and Kazakh populations at all loci. However, for the NWH group, significant genetic differences were observed with 37 reference populations, ranging from 1 to 17 loci. For the SCT group, significant genetic differences were observed with 46 reference populations, varing from 1 to 19 loci, and for the XKU group, significant genetic differences were observed with 47 reference populations, spanning 1 to 15 loci. Subsequently, all three investigated populations showed the largest R_st_ with the South Africa amaXhosa population, for which the R_st_ values were 0.1913 (NWH), 0.2240 (SCT) and 0.2140 (XKU). The smallest R_st_ values were 0.0072 (between the NWH and Central Chinese Han populations), 0.0181 (between the SCT and Sichuan Liangshan Yi populations) and 0.0324 (between the XKU and Xinjiang Uygur-3 populations). The NWH and SCT populations had a relatively small R_st_ with the Han, Yi, Tibetan and Korean populations, and the XKU ethnic group had a relatively small R_st_ with the other three Uygur and Kazakh populations.

For the purpose of ascertaining the genetic similarity and diversity between our three studied populations and 14 previously reported Chinese populations (Han^9,15,16,18–21^, Yi^16^, Tibetan^9,16^, Uygur^9,17,23^ and Kazakh^22^ populations), Nei’s standard genetic distances were calculated (Supplementary Table S14 and Fig. S2). The NWH population had a smaller R_st_ with the Han population, the SCT group had relatively closer genetic distances with the Yi and other Tibetan populations, and the XKU ethnic minority had genetic affinities with other Uygur and Kazakh populations.

### **Multidimensional scaling an**d principal component analysis

**Figure 1:**
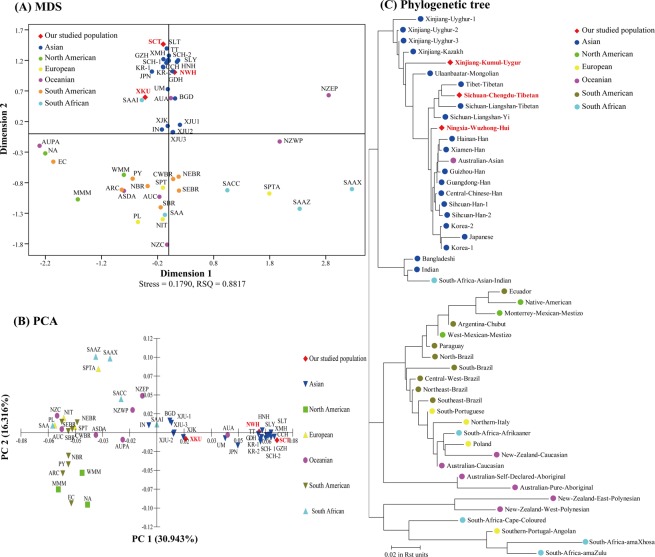
Population comparison analysis between our three studied populations and 47 previously published worldwide populations. (**A**) An MDS plot based on the genetic distance (R_st_) of 20 expanded CODIS loci was drawn. All populations are marked by their abbreviations. (**B**) The PCA plot was performed based on the allele frequencies of 20 expanded CODIS loci. All populations are marked by their abbreviations. (**C**) The phylogenetic tree was constructed based on R_st_ values with the neighbor-joining method. The population abbreviations are listed in Supplementary Table S2.

**Figure 2:**
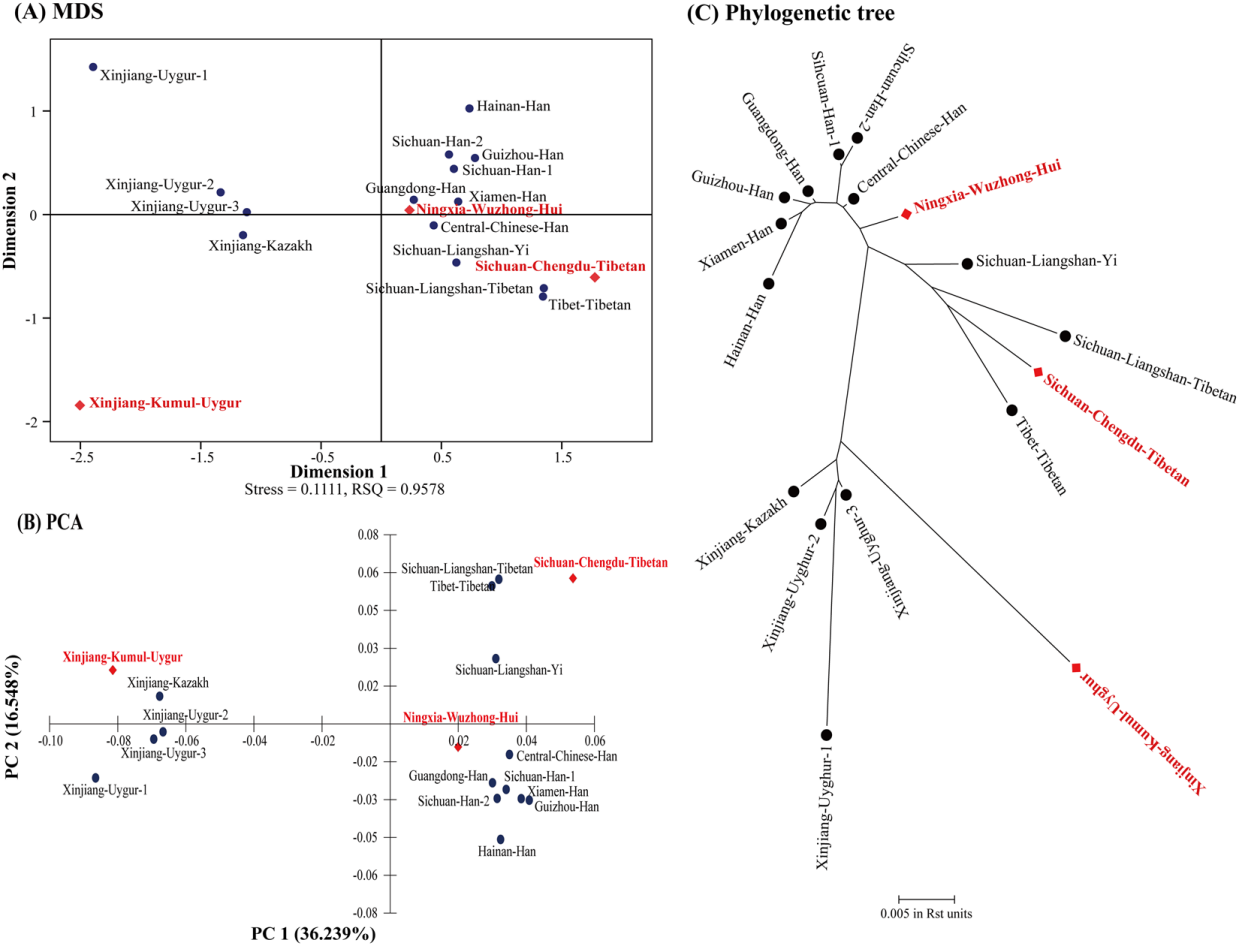
Population comparison analysis between our three studied populations and 15 previously published populations in China. (**A**) The MDS plot based on the genetic distance (R_st_) of 20 expanded CODIS loci was drawn. (**B**) The PCA plot was performed based on the allele frequencies of 20 expanded CODIS loci. (**C**) The phylogenetic tree was constructed based on R_st_ values with the neighbor-joining method. Red and bold fonts are our research groups.

To further explore the genetic relationships among the 50 populations, a multidimensional scaling (MDS) plot using R_st_ values was drawn by SPSS v19.0 software. As demonstrated in Fig. 1A, our three studied populations and most Asian populations were distributed in the middle-upper part of the x-axis. Among them, the NWH and SCT populations grouped together with the Han, Yi, Tibetan, Korean and Japanese populations; however, the XKU group was relatively farther away from these populations. Most European, South American and some Oceanian populations were located in the middle-lower part of the x-axis, and other populations were scattered around in the MDS. For comparisons among Chinese populations, our three studied groups separated from each other (Fig. 2A). The NWH population gathered together with the Han group and was distributed in the first quadrant, the SCT group was situated in the fourth quadrant with the Central Chinese Han, Yi and Tibetan ethnic groups, and the XKU and remaining three Uygur and Kazakh populations were distributed in the left part of the y-axis.

As shown in Fig. 1B, a principal component analysis (PCA) plot was illustrated based on allele frequencies of 20 expanded CODIS loci by MVSP v3.22 software^43^. The first principal component accounted for 30.943% of the variation and the second accounted for 16.316%. Four conglomerate groups were observed: the first group mainly consisted of our three studied populations and most Asian populations and was located at the middle-right part of the y-axis. The NWH and SCT ethnic groups densely aggregated with the Han, Yi, Tibetan and Korean populations, and the XKU population intensively grouped with other Uygur, Kazakh and Bangladeshi populations; the second group included most European and Oceanian populations, as well as some South American and South African populations, and was located at the middle-left part of the y-axis; the third one contained the North American and four South American populations and was positioned at the lower-left part of the graph; and the last group contained the Southern Portugal Angolan and two South African populations and was clustered at the upper-left part. In addition, a PCA diagram of 17 Chinese populations (Fig. 2B) indicated that the NWH population had genetic similarity with the Han population, the SCT ethnic group had close relationships with other Tibetan and Yi ethnic groups, and the XKU group clustered with other Uygur and Kazakh minority ethnicities.

### Phylogenetic tree reconstruction

To further analyze the population genetic background of our three studied populations, a phylogenetic tree was reconstructed via the neighbor-joining method based on R_st_ values by using Molecular Evolutionary Genetics Analysis v7.0.26 (MEGA v7.0.26) software^44^. As shown in Fig. 1C, three main branches were clustered: the lower branch was clustered by Southern Portugal Angolan, two Oceanian populations (New Zealand East Polynesian and New Zealand West Polynesian) and three South African populations (Cape-Coloured, amaXhosa and amaZulu); the middle one was divided into two subbranches and contained the American, most European and the Oceanian populations; the upper one was also split into two subclades, our three studied populations, eighteen Asian populations and the Australian Asian population clustered in the upper subclade, while the lower subclade consisted of the Bangladeshi, Indian and South Africa Asian Indian populations. For our three investigated ethnic groups, the NWH group clustered together with the Han, Australian Asian, Japanese and Korean populations and then clustered with the SCT, Yi and other Tibetan populations; however, the XKU population first tended to cluster together with Kazakh and then with other Uygur populations.

Furthermore, as shown in Fig. 2C, the cladogram of 17 Chinese populations also grouped into three branches. The NWH population clustered with the Han group, the SCT population clustered with the Yi and other Tibetan ethnic groups, and the XKU group clustered with other Uygur and Kazakh populations, which made the genetic relationships between our three studied populations and 14 Chinese populations clearer.

### Population structure analysis

**Figure 3:**
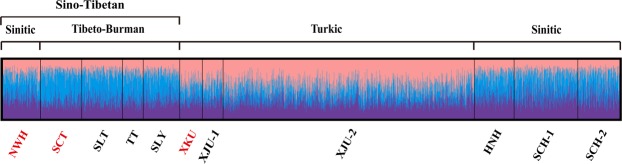
The STRUCTURE plot based on 23 STRs included in the Huaxia Platinum System with the optimum cluster number K = 3. Each vertical line stands for data of an individual partitioned into K clusters whose lengths denote the proportion to the estimated component in each of the deductive K groups. Red fonts represent our studied populations and the population abbreviations are displayed in Supplementary Table S2.

To illustrate the genetic structure of our three studied populations, a STRUCTURE plot was implemented based on the genotype data of 23 STRs by combining eight previously published populations^7,13–15^. The probable admixture levels and cluster membership patterns of each population are presented in Fig. 3 and Supplementary Table S15, and the optimum K value was three. The NWH and SCT populations shared similar membership proportions with other Sino-Tibetan populations, while the XKU group had similar admixture levels with other Turkic populations and was different from Sino-Tibetan populations. The proportion of membership of the Sino-Tibetan populations in cluster 2 ranged from 37.17% to 49.03% and was approximately 10% to 20% higher than the Turkic populations (24.18% to 26.93%). In contrast, the proportion of membership of the Sino-Tibetan populations in cluster 3 varied from 20.91% to 26.65% and was approximately 20% lower than the Turkic populations (41.94% to 45.13%).

## Discussion

Three minority ethnicities, the Hui, Tibetan and Uygur, have always been a hot topic in research by linguists, anthropologists, archaeologists and population geneticists^10,17,45,46^. However, the limited genetic research of the three populations in China is far from sufficient. In this study, we focused on the genetic makeup and phylogeny of the three ethnic groups using 23 autosomal STRs included in the Huaxia Platinum System. First, we confirmed that these STR loci conformed to HWE and that there was no LD existing among them. Thus the 23 STRs could be used as independent genetic markers for forensic investigation and population genetics in subsequent research. The assay performed well in the three populations and it could be a robust and effective tool for forensic applications, such as for individual identification and paternity tests.

Population substructure dissection is pivotal in population genetic studies, genotype-phenotype association research and forensic genetics, especially in East Asia with its high cultural, ethnical and geographical diversity. We performed population comparisons based on 20 autosomal STR loci (more loci can provide a higher refinement in the construction of a phylogeny than other similar studies) and found that the results of the interpopulation genetic distances, MDS, PCA and phylogenetic tree were consistent. For population comparisons worldwide, only Asian populations can be distinguished from other continental populations, which proved that the Huaxia Platinum System is useful for the identification of Asian populations. For population comparisons within China, the NWH group showed significant genetic homogeneity with the Han, Yi and Tibetan populations, which supports the origin theory of Hui via simple cultural diffusion^45^. The SCT population also had genetic affinities with the Han and Yi populations, and our results indicated that the high altitude Tibetan (Tibet Tibetan) and the low altitude Tibetan (SCT) populations had strong genetic similarity, this feature is maintained in accordance with previous findings and is indicated by high-throughput genotyping and sequencing data^47–49^. Although the XKU had close genetic relationships with other Uygur and Kazakh ethnic groups, it was not as close as that between other Uygur groups. This may be caused by the special geographical position and history of Kumul. Kumul is a multiethnic region (according to the 2010 population census, the Han Chinese accounted for 68.4%, while the Uygur, Kazakh, Hui, Mongolian and other ethnic minorities accounted for 31.6%) and is located at the border of Gansu Province in the East and Mongolia in the North, which is the throat of the Silk Road and the gateway to inland China. It was founded by a people known in Han Chinese source during the 1st millennium BCE, and the Uygur people and other minority ethnicities gradually settled in this area subsequently^50,51^. Furthermore, structure analysis based on the 23 autosomal STRs manifested that the populations of the same language family had a similar genetic makeup, and this was consistent with the results of population comparison analysis, namely the genetic structure is strongly correlated with linguistic affiliations^13^.

To further understand the genetic background and differentiation of Sino-Tibetan language (Han, Tibetan, Hui and Yi) populations, we are looking for and are validating a large number of new genetic markers with ethnic distinguishing potency. Large-scale population genetic studies using different high-density genetic markers and even whole-genome sequencing (WGS) will be conducted in the future.

## Materials and Methods

### Ethics Statement

Blood samples were collected with the approval of the Ethics Committee at the Institute of Forensic Medicine, Sichuan University. Written informed consent was obtained from all participants. All methods were conducted according to the guidelines approved by the Institute of Forensic Medicine, Sichuan University, and this study was ratified by the Ethics Committee of Sichuan University (Approval Number: K2015008).

### Sample collection, DNA isolation and quantification

Blood samples were collected from 493 unrelated individuals belonging to three Chinese ethnic minorities. These individuals included 183 Hui individuals living in Wuzhong City in Ningxia Hui Autonomous Administrative Subdivision, 200 Tibetan individuals residing in Chengdu City in Sichuan Province and 110 Uygur individuals located in Kumul City in the Xinjiang Uyghur Autonomous Region of Northwest China. During the sample collection process, we ensured that each individual’s family has been living in the respective region for at least three generations and did not intermarry with other ethnic groups and that any two individuals had no kinship within a minimum of three generations.

We isolated the genomic DNA with the PureLink^®^ Genomic DNA Mini Kit (Thermo Fisher Scientific, USA) according to the manufacturer’s instructions. The 7500 Real-time PCR System (Thermo Fisher Scientific, USA) was used to quantify the DNA concentration with the Quantifiler Human DNA quantification kit (Thermo Fisher Scientific, USA). Then, the DNA samples were normalized to 1 ng/μL and stored at −20 °C until amplification.

### PCR amplification and DNA typing

A total of 25 forensically related genetic makers (CSF1PO, FGA, TH01, TPOX, VWA, D1S1656, D2S1338, D2S441, D3S1358, D5S818, D7S820, D8S1179, D10S1248, D12S391, D13S317, D16S539, D18S51, D19S433, D21S11, D22S1045, D6S1043, Penta D, Penta E, Amelogenin and one Y-InDel of rs2032678) were coamplified using the Huaxia Platinum PCR amplification system. Multiplex amplification was conducted on a ProFlex^TM^ PCR system (Thermo Fisher Scientific, USA) following the manufacturer’s protocol. The 25 μL PCR volume contained 10 μL of master mix, 10 μL of primer set, 4 μL of deionized water and 1 μL of template DNA. Thermal cycler conditions were as described below: predenaturation at 95 °C for 1 min, followed by 26 cycles of 94 °C for 3 s, 59 °C for 16 s, and 65 °C for 29 s, and a final extension at 60 °C for 5 min. Separation and analysis of PCR amplified products was performed on the Applied Biosystems 3500 Genetic Analyzer (Thermo Fisher Scientific, USA) using POP-4 polymer (Life Technologies, USA), and injections were conducted at 1.2 kV for 16 s. Allele identification was conducted using the Huaxia Platinum panels, bin sets, stutter files and a 175 relative fluorescence units (RFU) threshold, unless otherwise stated, and were compared with the allele ladder provided by the corresponding kit via Applied Biosystems GeneMapper ID-X version 1.2 software.

### Statistical analysis

Allele frequencies, forensic statistical parameters (containing the match probability (MP), power of exclusion (PE), polymorphism information content (PIC) and typical paternity index (TPI)) and p values of the Hardy-Weinberg equilibrium test (HWE-p) of 23 autosomal STR loci were calculated in the modified PowerStat spreadsheet (Promega, Madison, WI, USA). Subsequently, the observed heterozygosity (Ho), expected heterozygosity (He) and p values of the linkage disequilibrium test (LD-p) were assessed in Arlequin v3.5 software^52^. Interpopulation differentiation was performed in Arlequin v3.5 software to compute pairwise F_st_ using locus-by-locus pairwise population comparisons. Nei’s genetic standard genetic distance (R_st_) was calculated using the Phylip3.695 package. Multidimensional scaling analysis (MDS) was conducted by SPSS software (IBM SPSS, version 19.0, Chicago), and principal component analysis (PCA) was carried out based on allele frequencies using MVSP v3.22 software^43^. The phylogenetic tree based on the neighbor-joining method was delineated in the Molecular Evolutionary Genetics Analysis v7.0 (MEGA v7.0) software^44^. The genetic distribution of 11 populations was conducted using STRUCTURE v.2.3.4 software^46^, for which the parameters were a100,000 lenth of burnin period after 100,000 steps for the Markov Chain Monte Carlo (MCMC) using the standard admixture model, five independent runs for each K value and K values from 2 to 7.

### Quality controls

Our laboratory is accredited by ISO 17025 and the China National Accreditation Service for Conformity Assessment (CNAS). The recommendations published by the DNA Commission of the International Society for Forensic Genetics (ISFG) were followed in the overall experimental procedure. The positive control of Control DNA 007 and negative control of ddH_2_O in each batch of genotyping was conducted.

## Supplementary information

Supplementary materials
