# Supplementary material for: Genetic polymorphism and phylogenetic differentiation of the Huaxia Platinum System in three Chinese minority ethnicities
Source: Sci Rep. 2019 Mar 4;9:3371. doi: 10.1038/s41598-019-39794-y (PMC6399324; doi:10.1038/s41598-019-39794-y)
Supplement: Supplementary file 1 — Supplementary materials [file 41598_2019_39794_MOESM1_ESM.pdf]

Supplementary Figures S1-S2 and Supplementary Tables S1-S15

Genetic polymorphism and phylogenetic differentiation of the Huaxia Platinum System in three Chinese minority ethnicities

Jing Liu[#], Zheng Wang[#], Guanglin He, Mengge Wang, Yiping Hou*

Institute of Forensic Medicine, West China School of Basic Medical Sciences & Forensic Medicine, Sichuan University, Chengdu 610041, China

[#] These authors contributed equally to this work

* Corresponding author:

Yiping Hou

Institution: Institute of Forensic Medicine, West China School of Basic Medical Sciences & Forensic Medicine, Sichuan University, Chengdu 610041, China

Phone: +86-28-85501550; Fax: +86-28-85501549.

Email address: profhou@yahoo.com, forensic@scu.edu.cn

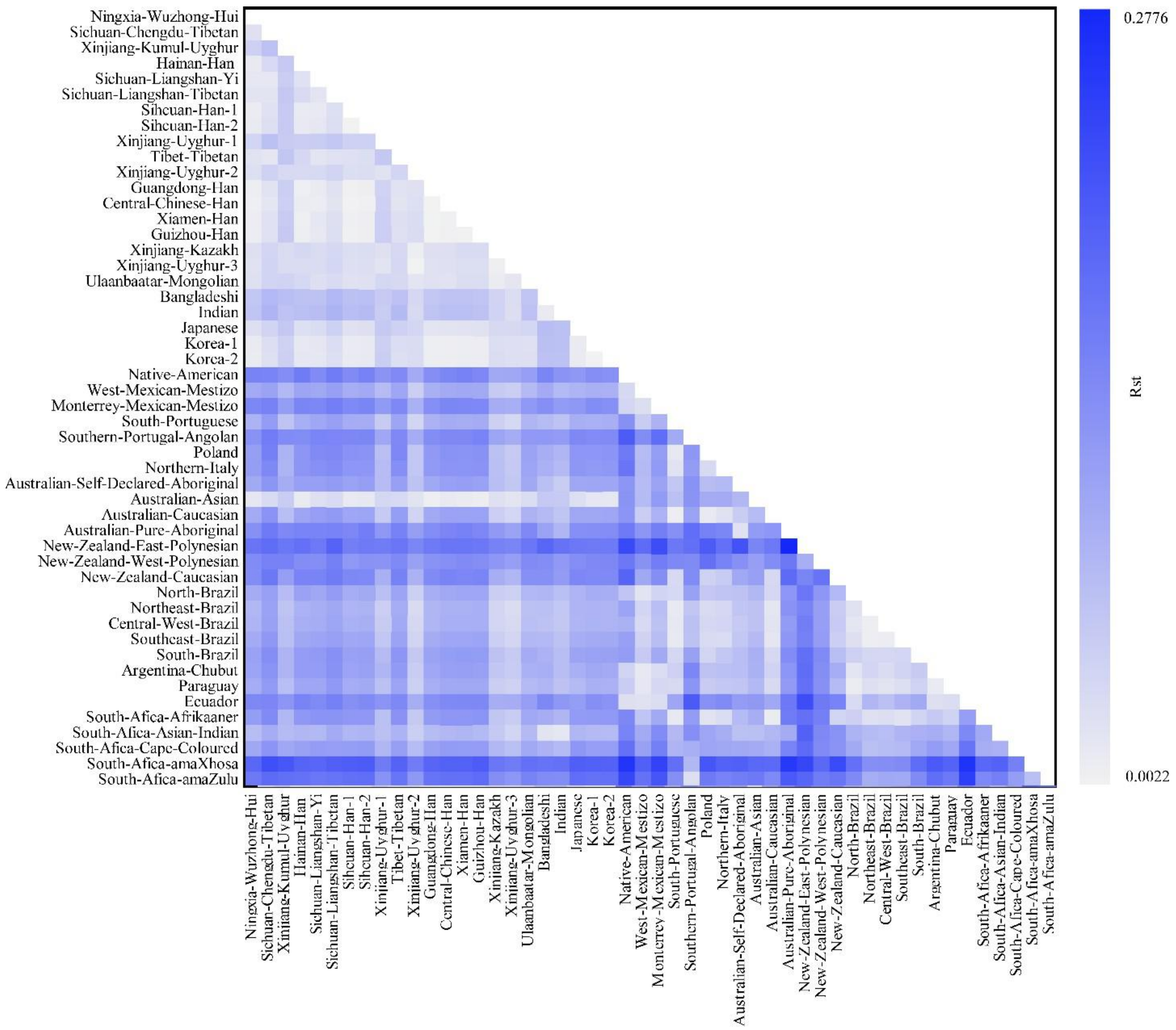

Supplementary Fig. S1. Plots of Nei's standard genetic distances (R_{st}) of our three studied populations and 47 previously published worldwide populations.

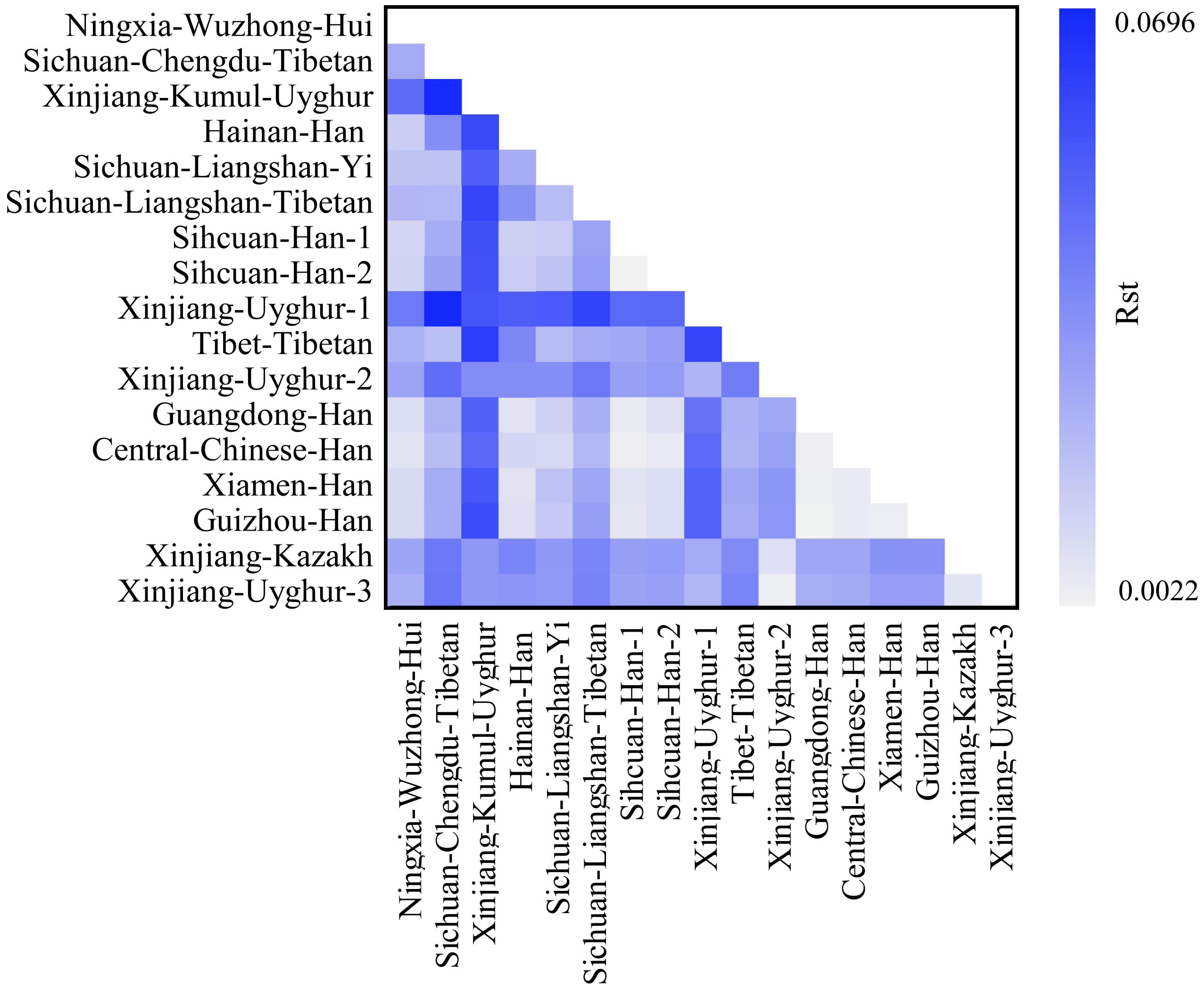

Supplementary Fig. S2. Plots of Nei's standard genetic distances (R_{st}) of our three studied populations and 14 previously reported populations of China.

Supplementary Table S1. The loci included in the Huaxia Platinum system and two other PCR amplification kit.

Loci	Expanded CODIS loci	Huaxia Platinum autosomal STR genotyping kit	GlobalFiler PCR Amplification Kit	Powerplex Fusion System
AMEL		√	√	√
CSF1PO	√	√	√	√
D10S1248	√	√	√	√
D12S391	√	√	√	√
D13S317	√	√	√	√
D16S539	√	√	√	√
D18S51	√	√	√	√
D19S433	√	√	√	√
D1S1656	√	√	√	√
D21S11	√	√	√	√
D22S1045	√	√	√	√
D2S1338	√	√	√	√
D2S441	√	√	√	√
D3S1358	√	√	√	√
D5S818	√	√	√	√
D6S1043		√		
D7S820	√	√	√	√
D8S1179	√	√	√	√
FGA	√	√	√	√
Penta D		√		√
Penta E		√		√
TH01	√	√	√	√
TPOX	√	√	√	√
vWA	√	√	√	√
SE33			√	
Yindel		√	√	
DYS391			√	√

Supplementary Table S2. The details of relevant populations.

Population	Full name	Population size	Continent	Abbreviation
P1	Ningxia-Wuzhong-Hui	183	Asia	NWH
P2	Sichuan-Chengdu-Tibetan	200	Asia	SCT
P3	Xinjiang-Kumul-Uygur	110	Asia	XKU
P4	Hainan-Han	193	Asia	HNH
P5	Sichuan-Liangshan-Yi	177	Asia	SLY
P6	Sichuan-Liangshan-Tibetan	198	Asia	SLT
P7	Sihcuan-Han-1	309	Asia	SCH-1
P8	Sihcuan-Han-2	202	Asia	SCH-2
P9	Xinjiang-Uyghur-1	100	Asia	XJU-1
P10	Tibet-Tibetan	100	Asia	TT
P11	Xinjiang-Uyghur-2	1218	Asia	XJU-2
P12	Guangdong-Han	1533	Asia	GDH
P13	Central-Chinese-Han	1130	Asia	CCH
P14	Xiamen-Han	1006	Asia	XMH
P15	Guizhou-Han	2018	Asia	GZH
P16	Xinjiang-Kazakh	748	Asia	XJK
P17	Xinjiang-Uyghur-3	1962	Asia	XJU-3
P18	Ulaanbaatar-Mongolian	267	Asia	UM
P19	Bangladeshi	188	Asia	BGD
P20	Indian	357	Asia	IN
P21	Japanese	1501	Asia	JPN
P22	Korea-1	526	Asia	KR-1
P23	Korea-2	1000	Asia	KR-2
P24	Native-American	533	North America	NA
P25	West-Mexican-Mestizo	238	North America	WMM
P26	Monterrey-Mexican-Mestizo	1008	North America	MMM
P27	South-Portuguese	502	Europe	SPT
P28	Southern-Portugal-Angolan	152	Europe	SPTA
P29	Poland	600	Europe	PL
P30	Northern-Italy	303	Europe	NIT
P31	Australian-Self-Declared-	318	Oceania	ASDA
P32	Australian-Asian	489	Oceania	AUA
P33	Australian-Caucasian	528	Oceania	AUC
P34	Australian-Pure-Aboriginal	588	Oceania	AUPA
P35	New-Zealand-East-	170	Oceania	NZEP
P36	New-Zealand-West-	122	Oceania	NZWP
P37	New-Zealand-Caucasian	159	Oceania	NZC
P38	North-Brazil	171	South America	NBR
P39	Northeast-Brazil	235	South America	NEBR
P40	Central-West-Brazil	228	South America	CWBR

P41	Southeast-Brazil	208	South America	SEBR
P42	South-Brazil	265	South America	SBR
P43	Argentina-Chubut	770	South America	ARC
P44	Paraguay	546	South America	PY
P45	Ecuador	1800	South America	EC
P46	South-Africa-Afrikaaner	106	South Africa	SAA
P47	South-Africa-Asian-Indian	102	South Africa	SAAI
P48	South-Africa-Cape-Coloured	113	South Africa	SACC
P49	South-Africa-amaXhosa	120	South Africa	SAAX
P50	South-Africa-amaZulu	100	South Africa	SAAZ

Supplementary Table S3. Genotypes of 23 autosomal STR loci included in the Huaxia Platinum system in three Chinese populations (n_{Hui} = 183, $n_{Tibetan}$ = 200, n_{Uygur} = 110).

Sample ID	CSF1PO	CSF1PO	D10S1248	D10S1248	D12S391	D12S391	D13S317	D13S317	D16S539	D16S539	D18S51	D18S51	D19S433	D19S433	D1S1656	D1S1656
Hui-001	11	11	13	13	17	22	9	11	9	9	12	13	13	15	12	17
Hui-002	11	12	15	16	20	20	8	11	9	9	13	20	13	13.2	14	15
Hui-003	10	10	12	13	17	21	11	11	11	12	13	16	13	14	16	16
Hui-004	12	12	13	15	18	21	10	11	11	13	14	14	13	16.2	15	18.3
Hui-005	10	12	15	16	18	22	10	13	9	12	13	16	12	14	15	17
Hui-006	11	12	14	15	18	19	8	11	9	10	13	17	14.2	14.2	15	16
Hui-007	12	13	13	15	18	22	8	11	9	10	14	16	13	14	11	17
Hui-008	11	12	15	16	15	17	8	12	9	9	16	20	13	13	16	16
Hui-009	11	12	14	14	19	24	9	11	9	12	16	16	14	14.2	15	16
Hui-010	11	14	15	16	17	18	9	10	9	11	12	15	14	14.2	13	14
Hui-011	10	10	14	15	20	20	8	11	9	12	13	21	13.2	14	11	15
Hui-012	9	12	14	15	18	18	10	11	10	11	14	16	14	14	16	18.3
Hui-013	10	12	13	14	18	22	8	11	11	11	13	16	14.2	14.2	15	17.3
Hui-014	12	12	13	13	17	19	8	13	11	11	15	20	14	15.2	12	15
Hui-015	10	13	12	12	18	19	11	11	8	10	12	14	13	14	11	15
Hui-016	11	12	14	15	19	19	8	11	11	11	13	19	13	15.2	13	17
Hui-017	11	11	14	15	18	21	9	10	12	12	13	16	14	15	14	15
Hui-018	12	12	13	15	18	21	8	12	9	11	14	16	12	15.2	15	16
Hui-019	12	13	13	14	19	23	9	11	11	12	13	14	14	15	12	16
Hui-020	11	12	14	14	17	18	9	11	11	11	18	19	13	14	13	15
Hui-021	11	12	13	14	19	20	12	12	11	11	19	20	14	15.2	14	16
Hui-022	10	10	13	13	20	22	8	13	11	11	14	20	13	15.2	14	16
Hui-023	10	13	12	13	18	19	9	10	9	11	15	20	14	15.2	13	16
Hui-024	9	11	12	13	19	21	8	11	10	11	14	25	13	13	14	17.3
Hui-025	10	12	14	15	20	21	10	10	9	13	12	17	14.2	16.2	14	14
Hui-026	10	11	13	14	20	20	8	11	9	12	15	15	13	13.2	16	17
Hui-027	12	13	13	13	20	20	9	12	9	11	11	20	14.2	15	16	17.3
Hui-028	10	12	13	13	19	22	8	12	11	13	14	15	13	13	16	16
Hui-029	13	13	15	17	18	18	11	11	11	12	14	15	11	15.2	15	18.3
Hui-030	12	12	13	14	18	18	9	11	9	10	13	16	14	15.2	16	16
Hui-031	12	12	13	15	17	18	8	8	11	12	16	17	15	15.2	11	12
Hui-032	10	13	13	17	17	20	11	12	11	11	14	16	12	14	15	18.3
Hui-033	11	11	15	15	17	19	11	12	11	11	15	16	13	14	15	16
Hui-034	12	12	13	14	20	24	8	11	10	10	15	16	12	14	16	17
Hui-035	12	12	14	14	19	24	9	12	9	13	13	16	14	15.2	14	15
Hui-036	11	11	15	15	17	20	8	11	9	14	13	15	13	14.2	11	16
Hui-037	10	10	14	15	18	19	8	8	9	10	13	13	14	14.2	13	15
Hui-038	9	12	12	13	18	20	11	11	8	9	14	19	14	15.2	11	17
Hui-039	11	12	13	16	15	20	8	9	11	11	14	15	13	16	15	18
Hui-040	10	11	13	14	18	21	8	12	11	12	13	15	12	13	15	16
Hui-041	10	12	13	14	17	18	11	12	8	11	14	16	12	14.2	14	16
Hui-042	10	11	13	15	16	17	8	11	9	9	16	17	13	13	12	15
Hui-043	11	12	13	14	18	21	8	12	9	11	18	20	14	14	15	18.3
Hui-044	12	12	14	15	17	20	8	12	11	12	21	22	12	13	15	16
Hui-045	9	11	13	15	18	20	8	11	11	11	13	17	14	16.2	15	16.3
Hui-046	11	11	14	15	18	20	8	8	11	13	12	16	13	16	17	17.3

Hui-047	10	11	14	14	18	21	8	10	12	13	15	19	13	16.2	13	15
Hui-048	9	12	13	15	20	20	8	10	9	10	13	13	13	15	13	16
Hui-049	11	11	14	15	17	19	10	11	11	11	18	19	13	15	16	16
Hui-050	10	12	13	15	17	17	11	11	11	12	12	16	12	14	11	14
Hui-051	10	13	14	14	19	21	11	13	12	13	13	15	13	15	13	14
Hui-052	12	12	12	13	18	20	10	13	9	10	16	19	14	14	15	17
Hui-053	10	12	13	16	18	18	10	11	11	11	13	17	14	15.2	16	17.3
Hui-054	12	12	13	14	21	22	8	12	9	9	17	18	15	15.2	15	17
Hui-055	11	11	12	15	18	20	8	11	11	12	13	18	13	13	16	17
Hui-056	10	12	13	14	20	20	10	12	9	12	13	16	14	15.2	14	19.3
Hui-057	10	12	12	15	18	20	8	10	11	11	13	19	13	13	13	13
Hui-058	10	10	13	15	16	19	12	12	10	12	14	15	12	15	12	16
Hui-059	12	12	13	15	18	19	8	9	10	12	14	14	13	14.2	15	16
Hui-060	10	12	13	15	17	23	10	11	10	12	14	16	14	15.2	12	16
Hui-061	11	12	13	14	17	18	11	11	11	11	13	16	14	16	11	18.3
Hui-062	12	13	11	15	19	21	10	10	9	12	14	15	14	14.2	13	16
Hui-063	11	12	14	15	18	20	10	10	9	12	13	16	12	14	16	17
Hui-064	10	11	15	17	18	23	8	11	9	13	17	22	15.2	16.2	17	18.3
Hui-065	10	13	12	13	22	23	11	14	9	13	13	14	13	13	15	17.3
Hui-066	10	12	13	14	18	18	8	8	10	13	14	16	14	15	15	18.3
Hui-067	11	12	13	15	19	22	8	9	11	13	13	13	13	15.2	16	16
Hui-068	10	11	14	16	17	21	8	11	11	12	14	14	13	13	11	15
Hui-069	10	11	14	14	19	22	10	10	9	12	14	14	13.2	15.2	13	14
Hui-070	10	12	13	15	17	19	8	11	11	11	13	14	13	15.2	11	14
Hui-071	12	12	8	15	20	22	8	11	10	11	14	23	14.2	16	16	17
Hui-072	12	12	13	15	21	22	8	8	9	11	13	14	12	13	15	16
Hui-073	10	11	13	14	19	21	8	12	11	12	13	16	12	15.2	16	16
Hui-074	10	11	12	17	17	20	9	9	9	11	13	18	13	14.2	15	15
Hui-075	12	13	13	14	19	22	8	11	12	12	14	14	14	14	13	16
Hui-076	11	12	13	14	19	20	8	12	12	13	14	17	14.2	15	14	15
Hui-077	12	13	14	14	22	23	8	9	9	9	14	20	13	16.2	14	16
Hui-078	12	12	13	13	17	22	9	11	9	12	16	20	13	14	15	15
Hui-079	9	11	13	13	20	20	9	11	11	12	13	19	13	14	16	16
Hui-080	10	12	15	15	18	18	11	14	11	12	13	16	12.2	15.2	14	17.3
Hui-081	10	12	12	13	18	20	8	11	9	10	13	15	12	13	13	19.3
Hui-082	11	13	12	13	19	21	11	12	11	12	15	17	13	14	13	16
Hui-083	12	12	12	15	19	20	8	11	9	12	12	18	13	14.2	14	16
Hui-084	10	11	13	14	17	21	8	9	9	9	15	17	14	15	15	15
Hui-085	10	11	13	14	22	23	11	13	11	13	13	13	14	15.2	15	15
Hui-086	9	14	13	16	19	23	9	11	9	13	13	14	14	16.2	15	17.3
Hui-087	11	12	14	16	17	18	10	14	11	11	16	19	14	14	15	16
Hui-088	10	11	13	14	18	22	11	11	9	10	15	18	13.2	14.2	15	16
Hui-089	12	13	13	15	18	21	8	12	11	11	13	16	13.2	15.2	17.3	17.3
Hui-090	9	12	13	15	19	22	8	11	10	13	14	16	13	14	15	18
Hui-091	10	13	13	16	20	21	10	11	9	11	14	22	14	14	12	16
Hui-092	10	11	13	14	19	19	8	11	11	11	13	13	13	14.2	11	14
Hui-093	10	12	13	15	22	23	11	13	8	9	12	13	14.2	14.2	14	14
Hui-094	12	12	14	14	17	20	9	10	9	11	13	16	14	14	15	16
Hui-095	10	12	13	15	18	23	11	11	11	12	13	17	13	14.2	17	17

Hui-096	11	12	13	15	22	23	10	10	9	12	13	13	13	15	12	15
Hui-097	10	11	12	13	17	20	12	12	9	11	15	18	13	16.2	11	16
Hui-098	10	12	13	13	18	26	9	11	9	10	15	18	15	16.2	14	18.3
Hui-099	10	12	14	14	19	19	9	11	9	11	13	15	13	14.2	13	14
Hui-100	11	13	14	16	19	21	9	11	10	11	11	18	14.2	15.2	15	17.3
Hui-101	10	12	13	14	20	22	8	10	9	11	15	17	13	14.2	15	16
Hui-102	10	12	13	16	18	18	9	12	12	13	13	14	13	13.2	16	16
Hui-103	10	12	13	14	18	19	11	12	11	12	14	19	13.2	15.2	15	17.3
Hui-104	12	12	13	14	19	20	11	12	11	11	17	19	13	13	15	15
Hui-105	11	11	12	15	18	19	11	11	9	9	14	15	13.2	14.2	11	15
Hui-106	11	12	15	16	18	23	11	12	9	9	14	15	14	15	16	16
Hui-107	10	11	14	15	19	19	11	13	11	13	14	16	13	14	13	16
Hui-108	10	11	14	16	18	19	11	12	11	12	17	22	14	15.2	16	16
Hui-109	10	12	12	15	17	18	8	9	11	11	15	17	12	15	13	15
Hui-110	11	12	14	16	18	21	8	10	9	9	13	13	11	13	12	17
Hui-111	10	11	13	14	19	20	11	12	9	10	14	16	13	14	16	17
Hui-112	12	12	16	17	21	21	11	11	9	11	13	14	12	14	13	16
Hui-113	12	13	13	14	18	22	8	11	9	10	13	18	13	14	14	16
Hui-114	10	10	13	14	17.3	21	9	13	9	11	14	16	14.2	14.2	16	18
Hui-115	10	11	15	15	17	22	9	11	9	14	16	18	14	14.2	16	17
Hui-116	11	12	13	14	19	21	8	10	10	11	14	17	12	13	15	17
Hui-117	11	12	12	14	19	20	10	12	9	9	14	14	14	14	15	17.3
Hui-118	10	12	12	13	17	22	8	8	9	10	16	16	13	15	15	16
Hui-119	10	12	16	16	18	22	8	14	9	14	13	14	13	15.2	15	15
Hui-120	7	11	14	15	18	19	8	10	9	13	15	22	14.2	16.2	12	14
Hui-121	12	14	13	14	19	20	8	12	9	9	13	14	13	13	12	13
Hui-122	11	12	14	16	18	20	8	10	9	10	16	16	14	16	13	16
Hui-123	10	11	14	14	19	22	10	11	10	11	15	15	13	14	14	17
Hui-124	9	12	12	14	19	19	8	11	9	9	13	22	13	15	15	15
Hui-125	10	11	14	15	19	20	8	11	9	11	15	17	14	14.2	15	16
Hui-126	12	12	13	13	18	22	12	12	11	12	13	18	14.2	14.2	15	17
Hui-127	10	11	13	14	18	20	11	11	9	13	13	14	13	14.2	13	17
Hui-128	12	12	13	14	17	23	8	9	11	12	13	18	13	14.2	15	15
Hui-129	12	15	13	15	18	20	9	12	12	12	18	19	14.2	16.2	15	15
Hui-130	12	12	13	13	18	26	11	11	9	9	14	14	14	15.2	12	15
Hui-131	10	12	13	15	17	20	11	13	9	11	13	17	14.2	15.2	14	17
Hui-132	11	11	12	15	18	20	8	11	9	11	15	21	14.2	15	16	16
Hui-133	12	12	12	12	18	19	8	9	12	12	14	14	13	14.2	13	15
Hui-134	8	11	14	15	20	21	8	8	9	13	13	18	13	14.2	14	16
Hui-135	10	11	13	16	15	19	11	12	12	12	17	17	14	14.2	15	17
Hui-136	12	12	13	15	18	18	10	13	13	13	15	18	14	14.2	15	17.3
Hui-137	9	13	13	15	18	19	9	11	11	11	13	15	14.2	15.2	16	16
Hui-138	10	12	13	14	22	23	8	12	9	11	16	22	14	15	15	15
Hui-139	10	10	12	12	18	20	9	11	10	12	13	22	13	13	15	15
Hui-140	10	12	15	15	18	18	9	12	9	11	13	17	13	15	12	15
Hui-141	12	12	12	14	19	19	8	10	9	9	13	13	14	15	14	17.3
Hui-142	10	10	14	17	18	19	9	10	12	13	12	13	13.2	14.2	11	17
Hui-143	11	12	12	13	18	22	10	10	10	11	12	17	13	15	11	15
Hui-144	12	13	15	16	18	20	8	8	9	11	14	16	13	15	11	15

Hui-145	12	12	14	15	17	21	11	13	11	12	15	15	13	15.2	16	18.3
Hui-146	9	10	14	15	19	19	9	11	10	11	11	15	13	15.2	15	16
Hui-147	12	13	13	15	20	20	8	11	11	12	14	18	13	15.2	11	11
Hui-148	12	12	14	15	19	21	8	14	11	12	11	15	13	13	15	16
Hui-149	10	12	13	15	20	20	8	8	9	10	15	18	12.2	15.2	15	17.3
Hui-150	10	11	14	15	17	19	10	12	10	14	16	19	14	15	13	15
Hui-151	8	11	14	14	18	19	8	9	11	11	15	18	13	15.2	11	15
Hui-152	11	12	13	14	18	20	10	11	9	12	12	16	12	15.2	15	16
Hui-153	11	12	15	16	18	23	8	9	10	12	14	15	14	14.2	11	14
Hui-154	11	12	13	14	20	21	8	13	11	12	13	14	13	14.2	15	16
Hui-155	10	11	13	14	18	19	8	10	9	9	13	14	14	14	15	16
Hui-156	10	11	13	15	18	19	9	12	11	12	13	16	14	16.2	11	13
Hui-157	11	12	13	15	18	18	11	12	9	11	17	24	13	14	15	15
Hui-158	10	13	13	13	17	23	8	10	9	12	14	20	13	13	15	15
Hui-159	10	12	13	15	19	20	8	10	9	11	13	18	14	15.2	15	15.3
Hui-160	10	13	13	15	18	20	10	10	11	13	15	16	13	14.2	11	13
Hui-161	10	12	13	16	18	20	8	11	11	11	15	15	13.2	14	16	16
Hui-162	10	11	14	16	16	19	9	12	9	12	13	13	13.2	15.2	11	11
Hui-163	12	13	14	14	17	18	8	10	9	11	13	13	13	14.2	15	16
Hui-164	10	10	13	14	19	22	10	12	9	12	13	14	14	15.2	13	15
Hui-165	10	12	13	15	19	21	9	10	10	12	13	16	13	14.2	15	16
Hui-166	10	11	13	14	19	24	8	10	11	13	16	18	14	15	15	16
Hui-167	11	12	13	14	18	20	8	8	9	12	14	15	14.2	14.2	14	15
Hui-168	10	10	12	15	17	23	10	11	11	11	14	17	13	15.2	13	15
Hui-169	10	11	15	16	17	20	12	12	12	12	15	15	13.2	14	16	16
Hui-170	10	10	13	15	20	23	11	12	11	13	14	15	14	15.2	15	16
Hui-171	9	11	15	17	19	22	8	9	8	13	12	16	14.2	15.2	15	16
Hui-172	10	11	14	15	17	20	8	12	11	12	14	14	13	14.2	16.3	17
Hui-173	12	12	16	16	18	19	8	10	9	10	12	18	13.2	14	16	16
Hui-174	10	12	13	15	17	18	11	12	9	13	13	15	13	14	12	17.3
Hui-175	12	12	14	15	17	19	8	11	11	13	12	18	14	14	13	14
Hui-176	11	12	13	15	18	20	9	11	9	9	14	16	14	14	11	16
Hui-177	11	12	14	15	20	22	8	11	9	13	14	18	14	15.2	15	16
Hui-178	11	11	13	13	18	18	8	11	11	11	14	14	14	16	16	17.3
Hui-179	12	13	13	13	20	20	10	12	9	11	13	22	13	15.2	15	16
Hui-180	12	12	15	17	18	24	9	12	12	12	13	15	13	15.2	15	16
Hui-181	11	12	14	15	22	23	9	11	9	12	13	15	14	14.2	15	18.3
Hui-182	10	12	14	16	19	21	8	11	9	12	14	16	16.2	16.2	15	16
Hui-183	10	12	12	13	18	24	11	12	9	11	13	20	14.2	15.2	12	16
Tibetan-001	12	13	13	16	18	19	8	11	11	14	13	15	13	15.2	13	15
Tibetan-002	11	12	13	14	18	18	9	13	9	10	13	13	13	14	11	17
Tibetan-003	11	12	15	15	18	18	8	11	11	12	13	17	13	14	16	17
Tibetan-004	7	12	14	14	18	18	8	9	10	10	13	17	13	14	13	13
Tibetan-005	9	11	12	14	19	21	10	12	11	12	14	21	13.2	14.2	12	17.3
Tibetan-006	11	12	13	13	18	21	10	12	9	11	13	13	13.2	16	15	16
Tibetan-007	12	13	13	13	19	22	11	13	12	12	14	23	14.2	16.2	13	15
Tibetan-008	9	10	15	15	20	20	8	12	11	12	13	20	14	14	17	17
Tibetan-009	11	12	15	15	18	19	11	11	11	11	15	16	14.2	15.2	13	17
Tibetan-010	10	12	13	15	18	20	10	12	9	9	13	15	14	14.2	11	16

Tibetan-011	9	12	12	15	17	23	8	12	9	11	13	18	13	14.2	15	16
Tibetan-012	11	12	13	16	18	22	9	13	11	11	13	14	14	14	13	15
Tibetan-013	12	12	14	15	17	22	9	10	10	13	14	15	14	14	12	12
Tibetan-014	9	11	14	17	20	20	8	11	11	11	13	22	14.2	15	13	15
Tibetan-015	11	12	12	14	18	19	12	12	10	12	13	14	13	13	15	16
Tibetan-016	11	12	13	14	18	19	12	12	9	11	13	13	13	14.2	16	17
Tibetan-017	12	12	14	14	18	19	8	12	9	11	13	14	14	14.2	15	17
Tibetan-018	11	11	13	15	19	20	8	10	11	12	13	14	15	15	16	17
Tibetan-019	12	12	12	14	21	22	10	14	11	11	14	14	13.2	15.2	15	15
Tibetan-020	11	12	16	16	17	18	8	9	9	12	13	15	13	13	13	19.3
Tibetan-021	11	12	13	14	18	20	8	11	10	11	17	19	13	16.2	13	16
Tibetan-022	11	11	12	13	18	22	11	13	9	10	17	19	13	13	15	17
Tibetan-023	10	10	13	15	17	21	8	11	13	13	14	18	13	13	11	14
Tibetan-024	10	13	12	15	18	22	8	10	10	11	14	15	14	14	14	16
Tibetan-025	10	12	14	15	17	17	8	12	13	13	18	23	13	14.2	11	16
Tibetan-026	12	12	13	16	17	18	9	12	12	12	14	14	14	14.2	12	15
Tibetan-027	12	14	15	16	18	19	12	13	11	12	16	17	13	14.2	12	15
Tibetan-028	11	12	12	16	17	19	8	10	12	13	14	16	13	14	15.3	16
Tibetan-029	11	12	14	15	19	20	12	13	10	11	13	13	14	15.2	14	16
Tibetan-030	10	12	14	15	18	18	8	12	11	11	15	22	14	14.2	13	14
Tibetan-031	12	12	16	17	21	23	8	12	10	10	13	17	14	15.2	16	17.3
Tibetan-032	11	12	13	15	19	23	8	12	10	11	17	20	12.2	13.2	13	18
Tibetan-033	12	12	14	14	18	19	9	12	9	11	13	13	13	13.2	13	14
Tibetan-034	12	12	13	15	18	23	8	11	10	12	14	16	12	13	12	16
Tibetan-035	10	12	13	13	17	18	10	11	10	11	13	14	13	15	12	15
Tibetan-036	11	12	13	16	19	21	12	12	9	10	13	13	13	14	15	15
Tibetan-037	10	12	13	15	17	19	9	13	11	12	13	14	14	14.2	13	16
Tibetan-038	10	12	13	15	19	21	11	12	11	11	19	20	13	15.2	12	12
Tibetan-039	11	12	13	17	20	20	8	12	12	13	13	15	14	14.2	13	17
Tibetan-040	10	12	14	15	17	22	8	12	9	10	13	15	14.2	15.2	12	13
Tibetan-041	11	12	14	15	19	21	8	11	9	11	13	14	13	15	11	16
Tibetan-042	10	10	13	14	20	20	10	10	11	11	13	16	13	14.2	12	17
Tibetan-043	11	11	13	13	17	21	9	11	10	11	14	17	13	15.2	15	15
Tibetan-044	10	12	13	16	18	18	9	11	11	11	12	21	13	14	13	17
Tibetan-045	10	12	13	15	19	21	8	12	9	11	14	17	13	14	15	17
Tibetan-046	10	12	15	16	18	18	10	12	8	11	15	17	14.2	15	15	17
Tibetan-047	12	12	12	13	18	23	10	12	11	12	13	16	13	15	13	13
Tibetan-048	12	12	12	13	18	20	9	10	9	11	13	16	13	15.2	12	13
Tibetan-049	10	11	13	15	17	20	10	12	10	12	13	15	14	14	17.3	17.3
Tibetan-050	12	13	13	14	19	20	11	13	12	12	14	15	14	15.2	14	15
Tibetan-051	10	12	13	14	17	21	11	12	11	13	14	14	13	13	14	17.3
Tibetan-052	10	12	13	15	17	19	11	14	11	11	13	15	14.2	15.2	13	14
Tibetan-053	12	12	15	16	18	19	9	10	11	11	17	20	13	14	13	15
Tibetan-054	12	13	13	15	19	20	12	12	12	12	13	13	13	15.2	16	17
Tibetan-055	12	12	13	13	22	23	9	11	9	13	13	15	14	15.2	15	17
Tibetan-056	10	12	13	16	18	18	11	12	10	12	14	14	14.2	15.2	11	15
Tibetan-057	10	11	12	13	21	24	9	10	12	13	13	18	13	15	17	17.3
Tibetan-058	10	12	13	14	17	21	12	12	13	13	14	23	13	14.2	16	17.3
Tibetan-059	10	10	13	14	18	19	11	12	9	10	13	17	14.2	15.2	15	16

Tibetan-060	10	12	16	16	18	18	10	11	9	10	13	21	15	15.2	15	15
Tibetan-061	11	13	13	15	20	20	9	12	11	12	14	17	14	14	13	16
Tibetan-062	12	12	15	15	18	18	8	13	10	11	14	14	14	16.2	11	15
Tibetan-063	10	12	13	14	18	19	11	12	9	12	13	17	13	14.2	13	17
Tibetan-064	10	11	15	16	18	21	8	10	11	11	15	24	13	15	16	17
Tibetan-065	11	12	13	14	17	18	11	12	11	12	13	16	12.2	15.2	11	12
Tibetan-066	11	12	15	16	18	19	9	12	12	13	14	15	13	13	12	16
Tibetan-067	9	13	13	14	18	19	11	14	8	10	14	18	13	13.2	11	16
Tibetan-068	9	12	13	14	18	18	8	11	10	12	13	15	13	14	13	18
Tibetan-069	10	12	14	15	17	18	8	10	9	9	14	16	14.2	15	16	16
Tibetan-070	11	12	12	16	19	19	8	10	10	10	12	15	13	15.2	11	18.3
Tibetan-071	12	13	13	15	17	19	9	13	12	13	13	16	13	14	11	15
Tibetan-072	11	12	13	13	18	22	9	9	9	13	12	19	14	14	13	16
Tibetan-073	10	12	14	14	18	18	9	11	12	12	12	16	13	14	12	15
Tibetan-074	10	10	13	15	18	19	8	9	9	13	14	22	16.2	16.2	12	15
Tibetan-075	11	11	14	16	19	19	8	13	9	9	13	14	13.2	14.2	12	16
Tibetan-076	12	12	13	15	19	22	12	12	10	11	13	16	14.2	14.2	13	13
Tibetan-077	7	11	13	16	19	23	8	12	9	9	14	16	13	14.2	16	16
Tibetan-078	11	12	13	13	18	18	9	11	11	12	13	16	13	13	15	16
Tibetan-079	10	12	13	13	19	20	10	11	9	13	14	16	14.2	15.2	13	13
Tibetan-080	11	12	13	13	18	20	9	11	11	12	17	21	15	15.2	15	16
Tibetan-081	10	10	15	15	18	20	11	12	10	13	13	16	14	15.2	11	16
Tibetan-082	10	10	13	14	18	19	8	12	10	10	13	18	14.2	15.2	16	16
Tibetan-083	12	13	13	15	20	20	8	8	10	11	14	17	14	15	16	17
Tibetan-084	12	12	13	15	15	17	8	11	10	11	16	17	13.2	14.2	12	15
Tibetan-085	10	12	13	14	19	19	7	12	9	13	16	22	13	14.2	11	15
Tibetan-086	11	12	13	13	23	24	10	12	11	12	16	16	14.2	15	13	16
Tibetan-087	12	12	13	13	18	21	11	12	11	11	13	22	13	14.2	12	17
Tibetan-088	12	12	14	15	18	19	8	8	9	11	13	16	13	14.2	15	19.3
Tibetan-089	12	13	14	17	19	19	11	12	11	11	21	22	13.2	15	12	13
Tibetan-090	11	12	13	16	19	20	10	11	11	11	13	14	13	13	15	17.3
Tibetan-091	9	12	12	15	18	19	10	13	11	11	15	16	13	15	15	17
Tibetan-092	9	12	13	14	17	20	8	9	9	11	14	15	13	15.2	13	15
Tibetan-093	12	13	13	14	18	20	8	13	9	12	13	13	15.2	16.2	16	16
Tibetan-094	12	13	13	14	19	20	12	12	11	12	13	20	13	15.2	12	15
Tibetan-095	12	13	13	13	17	18	8	12	11	13	15	15	13	16.2	15	16
Tibetan-096	10	11	15	15	19	23	10	12	11	15	13	14	13	14	15	17
Tibetan-097	10	12	13	15	18	18	8	8	12	13	15	19	14	15	17	18.3
Tibetan-098	12	12	13	14	18	21	9	10	10	11	20	21	13	13.2	15	16
Tibetan-099	10	10	13	14	18	20	11	12	10	10	13	13	14.2	15.2	15	16
Tibetan-100	9	11	15	15	18	20	10	12	9	11	14	14	13	16.2	17	17.3
Tibetan-101	11	12	13	13	17	19	11	11	9	11	13	13	13	14	13	13
Tibetan-102	12	12	13	13	18	18	8	12	9	11	13	14	14	14.2	15	16
Tibetan-103	10	12	13	13	17	18	10	12	10	12	14	17	13	15.2	16	16
Tibetan-104	11	12	13	13	17	22	11	13	9	11	13	19	14	14	15	17
Tibetan-105	11	12	13	15	19	19	11	12	11	12	16	16	14	15.2	13	15
Tibetan-106	9	12	12	13	19	20	10	11	10	12	15	15	15	15	14	16
Tibetan-107	10	12	13	16	19	23	9	10	9	11	13	13	13.2	14	15	17
Tibetan-108	10	10	13	14	18	19	9	14	11	12	13	21	14	15.2	15	16

Tibetan-109	11	11	15	16	17	18	8	12	9	12	13	13	14	14.2	15	16
Tibetan-110	11	12	13	15	17	18	8	12	12	13	14	19	13	15.2	13	15
Tibetan-111	13	14	13	14	18	18	8	14	11	12	13	19	13	14.2	16	17
Tibetan-112	10	12	14	15	18	18	10	11	9	9	13	14	13	15.2	15	17
Tibetan-113	10	12	12	12	18	20	8	11	9	10	12	13	13	14	15	17.3
Tibetan-114	12	12	13	13	18	20	12	14	9	14	14	14	13	13	13	17
Tibetan-115	10	11	16	17	18	19	8	11	9	14	14	21	13	15.2	15	16
Tibetan-116	10	12	13	13	17	20	9	11	9	12	13	15	14	14.2	15	15
Tibetan-117	10	12	13	14	18	18	11	11	11	12	12	17	14.2	15.2	12	16
Tibetan-118	11	11	13	13	20	20	10	12	9	9	13	15	13	15	16	17
Tibetan-119	11	12	13	15	22	23	8	13	10	11	14	15	14.2	15.2	15	16
Tibetan-120	10	12	14	14	19	20	8	11	12	12	13	14	15	15.2	14	16
Tibetan-121	11	11	13	17	18	21	11	12	10	12	14	15	14	14.2	16	16.3
Tibetan-122	11	12	13	15	19	21	10	11	13	13	13	14	13	13	14	15
Tibetan-123	12	12	13	15	18	22	10	14	9	14	17	19	13	15.2	16	16
Tibetan-124	12	12	13	15	18	20	11	13	10	12	21	22	13	15.2	11	12
Tibetan-125	10	11	12	15	18	18	9	10	10	11	13	15	14	14	15	15
Tibetan-126	12	12	13	15	19	19	11	12	10	11	22	22	14.2	16	14	17
Tibetan-127	11	12	13	16	19	21	8	12	9	12	13	14	14	14.2	16	18.3
Tibetan-128	11	13	13	15	19	20	8	12	9	11	13	13	13	13.2	12	15
Tibetan-129	10	12	13	15	19	24	9	9	9	11	14	14	14.2	16.2	12	15
Tibetan-130	10	11	16	18	20	21	8	12	10	11	13	16	13	13	12	17
Tibetan-131	11	12	12	13	18	18	12	12	9	11	19	19	13	14.2	14	18
Tibetan-132	12	13	13	15	18	19	10	12	9	12	13	14	15	15.2	15	15
Tibetan-133	10	11	12	17	17	19	8	9	10	11	13	14	13	16.2	12	13
Tibetan-134	12	13	13	17	17	19	9	11	10	11	15	15	13	15	14	16
Tibetan-135	12	12	13	13	18	18	11	11	9	10	13	15	14	14	14	17
Tibetan-136	10	11	13	13	18	19	11	11	9	10	14	15	13	14	15	16
Tibetan-137	12	12	12	15	16	19	8	11	9	11	16	17	13	15	15	16
Tibetan-138	12	14	14	15	18	19	12	12	10	11	13	16	13.2	15	15	16
Tibetan-139	10	10	13	16	20	21	12	13	9	11	13	13	14.2	14.2	16	16
Tibetan-140	11	12	13	14	18	19	10	12	10	11	13	13	13.2	16	13	17
Tibetan-141	10	12	14	15	18	19	13	13	11	11	13	19	14	14	12	16
Tibetan-142	11	12	13	15	17	21	9	10	11	12	14	15	13	16.2	16	17
Tibetan-143	11	13.1	13	14	20	24	8	9	10	12	15	19	14.2	14.2	12	16
Tibetan-144	12	13	12	15	18	18	8	9	11	14	15	20	14	14	11	12
Tibetan-145	11	11	14	15	19	20	11	12	11	11	13	14	13	13	12	16
Tibetan-146	12	12	15	15	18	19	9	13	11	12	13	15	14	14.2	11	12
Tibetan-147	11	12	14	14	18	20	8	8	11	12	15	19	13.2	15.2	17.3	17.3
Tibetan-148	12	12	12	14	19	21	11	11	11	12	14	15	12	14.2	13	15
Tibetan-149	10	11	12	13	17	22	12	12	11	13	16	17	13	14	12	16
Tibetan-150	12	12	13	13	19	19	11	12	11	12	13	13	14	14.2	12	16
Tibetan-151	12	12	12	15	19	21	11	12	9	11	15	15	13	14	13	14
Tibetan-152	11	11	12	15	20	24	9	10	9	13	13	14	14	14	17	17
Tibetan-153	11	12	13	13	18	19	8	12	9	12	16	21	13	13.2	16	17
Tibetan-154	10	12	13	14	17	23	11	12	9	9	13	17	14	15	14	15
Tibetan-155	12	12	13	16	17	24	12	13	11	13	14	16	13.2	14.2	11	16
Tibetan-156	12	12	13	13	20	20	10	11	10	13	13	17	15	15.2	13	15
Tibetan-157	11	12	13	15	17	21	8	10	9	11	13	20	12	14	16	16

Tibetan-158	9	12	13	16	17	19	9	12	10	11	13	20	13	13.2	13	17
Tibetan-159	10	12	13	16	17	21	8	12	12	14	16	21	13	14.2	17	17.3
Tibetan-160	12	12.1	14	14	17	24	12	12	11	13	13	15	14.2	15.2	13	14
Tibetan-161	10	12	14	15	18	19	10	11	11	13	13	16	12	13	11	15
Tibetan-162	7	11	13	14	19	19	11	12	11	13	13	14	13.2	14.2	14	16
Tibetan-163	10	11	12	15	19	23	8	13	11	12	13	17	15.2	15.2	16	16
Tibetan-164	11	12	13	13	19	20	8	12	9	12	15	17	14.2	15	12	17
Tibetan-165	10	12	12	14	17	18	12	12	11	11	14	16	13	14.2	11	15
Tibetan-166	11	11	13	15	17	18	9	12	9	9	13	13	13	14	16	17
Tibetan-167	10	12	13	14	19	19	8	9	9	11	14	14	13	15.2	14	16
Tibetan-168	10	12	13	14	18	20	11	12	11	14	17	21	14.2	14.2	11	12
Tibetan-169	10	10	13	16	19	22	11	12	11	13	13	16	13	14	12	17
Tibetan-170	12	12	13	13	18	19	8	9	11	12	13	17	12.2	14	15	16
Tibetan-171	11	12	13	13	18	19	8	11	10	11	16	21	13.2	14	16	18
Tibetan-172	11	12	13	16	19	21	11	11	11	13	13	22	14.2	15	15	17
Tibetan-173	12	12	13	13	19	21	9	12	9	11	15	17	14.2	15.2	15	17
Tibetan-174	11	12	13	15	19	23	8	9	9	12	12	17	13	14.2	13	13
Tibetan-175	12	15	13	14	18	22	10	12	11	13	13	15	13.2	15	15	16
Tibetan-176	7	12	13	14	17	23	11	12	10	12	13	16	15	16.2	14	16
Tibetan-177	12	13	12	14	18	19	9	11	13	13	14	16	13	14	15	17
Tibetan-178	10	12	13	15	17	22	13	14	12	12	16	23	13	15	14	15
Tibetan-179	11	12	13	14	18	18	8	10	11	11	12	13	14	14.2	12	19.3
Tibetan-180	10	12	12	16	18	19	8	13	8	8	13	19	14	16.2	15	17
Tibetan-181	10	12	13	15	18	19	12	14	10	13	14	16	14	14.2	15	17
Tibetan-182	11	12	13	16	23	24	10	11	11	11	17	19	14	14.2	13	17
Tibetan-183	10	12	13	14	18	19	11	12	11	12	16	16	13	13.2	14	16
Tibetan-184	12	13	13	13	18	19	8	12	11	12	16	21	13	14.2	15	17
Tibetan-185	12	13	13	15	19	19	9	11	9	14	13	14	15.2	15.2	15	15
Tibetan-186	12	12	15	16	19	21	8	12	9	9	15	17	14	14.2	15	16
Tibetan-187	10	12	14	15	18	21	10	11	11	12	13	21	13	14	13	16
Tibetan-188	12	12	13	16	19	20	10	11	11	12	19	22	14	15	12	14
Tibetan-189	10	12	14	14	19	24	8	8	9	10	13	20	13	14	12	17
Tibetan-190	10	11	12	15	18	22	8	8	9	12	13	15	15.2	16	12	16.3
Tibetan-191	10	12	13	15	18	22	11	11	9	14	12	13	14.2	14.2	13	17
Tibetan-192	11	12	12	14	18	20	8	13	11	13	19	25	13.2	13.2	14	17.3
Tibetan-193	12	12	13	14	17	18	10	11	9	10	16	20	13	15.2	16	17
Tibetan-194	11	12	14	15	21	22	8	8	11	12	14	19	13	14	11	17
Tibetan-195	10	13	14	14	18	21	8	9	9	12	17	18	14	14	15	16
Tibetan-196	10	11	12	15	19	19	11	12	11	11	14	17	14.2	16	14	15
Tibetan-197	9	10	13	14	20	25	10	11	8	11	13	14	13	13	9	16
Tibetan-198	11	12	13	14	18	23	12	13	9	12	13	14	14.2	16.2	13	15
Tibetan-199	11	12	12	12	18	20	8	13	10	13	18	19	13	15.2	15	16
Tibetan-200	10	12	13	14	19	20	8	8	11	11	14	16	14.2	15.2	12	17
Uygur-001	10	11	13	13	17	22	8	11	11	11	13	13	12	13	12	18.3
Uygur-002	9	12	13	17	18	19	11	11	10	12	12	13	13	14	15	15
Uygur-003	11	13	14	15	17	21	12	12	11	11	12	14	13	14	11	11
Uygur-004	11	12	13	17	20	22	11	12	11	12	15	16	13	15.2	11	16
Uygur-005	11	12	13	15	17	23	11	12	12	12	15	16	15	15.2	13	16
Uygur-006	11	12	13	17	18	23	11	12	11	12	13	17	15.2	16.2	11	13

Uygur-007	11	12	13	14	20	20	11	11	11	14	15	17	14.2	15	15	17
Uygur-008	10	12	13	13	17	21	11	11	12	12	14	17	13	15.2	13	16
Uygur-009	10	12	14	15	19	22	9	14	9	11	14	19	12	14	16	16
Uygur-010	10	10	14	15	17	20	10	12	12	12	13	13	14	14.2	11	13
Uygur-011	10	10	14	14	20	21	11	11	11	12	13	17	13	13	12	18.3
Uygur-012	10	11	13	17	20	20	12	12	9	11	12	16	13	16.2	15	15
Uygur-013	11	12	11	13	19	22	9	11	9	14	13	14	13	14	14	16
Uygur-014	12	12	12	13	15	19	10	11	11	13	15	17	13	15.2	11	16
Uygur-015	9	12	16	17	19	23	11	13	12	12	12	22	15	15.2	13	16
Uygur-016	10	10	13	13	22	23	9	11	10	11	14	21	13	15	15	16
Uygur-017	12	12	11	15	19	23	8	12	9	13	14	15	13	14.2	11	15
Uygur-018	10	12	15	15	21	23	13	14	11	12	13	14	13	13	16	16
Uygur-019	11	12	14	17	19	19	13	14	9	9	13	15	13	13.2	15.3	16
Uygur-020	10	11	13	16	18	19	10	12	9	12	12	16	13	14	14	16
Uygur-021	11	12	13	15	16	22	12	13	10	14	14	14	13	13.2	14	16
Uygur-022	10	11	11	13	19	19	11	12	12	13	15	21	13	16	14	16
Uygur-023	11	11	13	16	19	22	12	12	12	12	15	17	14	16	15.3	16
Uygur-024	10	10	15	16	19	21	12	12	9	12	15	18	14	15.2	11	18.3
Uygur-025	11	12	14	14	15	18	12	13	11	12	12	15	14	15.2	16	16
Uygur-026	10	11	13	14	20	22	8	11	11	12	15	16	15.2	15.2	16	16
Uygur-027	12	13	13	14	21	22	8	11	12	12	14	14	13	13	15	17
Uygur-028	11	12	13	14	20	21	11	14	11	11	15	15	14	14	14	17.3
Uygur-029	10	10	14	14	20	21	12	13	9	11	13	15	13	15	14	16
Uygur-030	10	10	13	15	17	19	11	12	9	11	13	15	15.2	16.2	12	13
Uygur-031	10	12	13	15	17	19	9	12	11	14	16	16	14	15	11	15
Uygur-032	10	11	13	18	19	21	11	11	9	9	14	16	13	14	14	15
Uygur-033	10	12	13	13	15	15	9	11	9	14	14	15	13	14	11	15
Uygur-034	10	14	16	16	18	19	11	13	12	12	15	20	12	14	17	17.3
Uygur-035	10	11	16	17	17	20	9	14	12	12	14	14	13	15.2	16	17.3
Uygur-036	10	12	14	14	19	22	9	11	13	13	14	14	13	16	12	15
Uygur-037	11	12	16	17	18	19	9	9	12	13	15	20	14	15	11	17.3
Uygur-038	11	12	13	15	18	18	10	11	11	13	17	17	13	13	15	15.3
Uygur-039	11	11	13	15	19	22	11	13	9	14	16	18	13	14	14	14
Uygur-040	10	11	15	16	17	18	11	14	9	13	13	15	15	15.2	15	18.3
Uygur-041	12	12	13	13	21	23	9	14	12	13	15	15	13	15.2	12	17.3
Uygur-042	10	11	13	14	17	18	8	11	9	11	15	16	14	16	11	16
Uygur-043	10	12	13	14	20	20	11	14	12	13	13	17	13	16	15	16
Uygur-044	10	12	13	15	19	20	11	13	11	13	14	16	13	13	15	16
Uygur-045	10	11	12	14	20	20	11	11	11	12	13	15	13	13	12	16
Uygur-046	10	12	13	15	18	19	11	13	9	12	13	14	14	15	11	16
Uygur-047	10	12	16	18	17	22	11	11	13	14	16	17	14	16.2	16	18.3
Uygur-048	12	12	13	14	17	17	10	12	9	12	17	20	14.2	14.2	13	19.3
Uygur-049	11	11	14	17	20	21	10	13	8	11	14	18	14	16.2	12	15
Uygur-050	11	11	15	17	17.3	18	11	14	11	12	13	14	13	14	15	17.3
Uygur-051	10	13	17	18	15	22	11	14	8	12	14	16	13	16.2	12	15
Uygur-052	10	12	13	16	18	19	9	10	9	11	15	16	14	15	13	17
Uygur-053	12	12	13	14	17	20	10	13	11	12	15	17	13	15.2	17.3	17.3
Uygur-054	10	12	16	17	18	19	11	13	9	12	12	13	14	14	15	16
Uygur-055	12	13	14	14	17	19	11	12	9	11	13	17	15.2	16.2	11	13

Uygur-056	11	12	14	17	17	18	9	13	11	12	13	18	13	15.2	11	15
Uygur-057	10	10	14	16	19	22	10	12	11	12	13	21	14.2	14.2	16	16
Uygur-058	9	10	13	13	17	18	9	10	10	12	13	15	13	14	12	16
Uygur-059	11	12	14	14	19	20	8	9	9	12	14	18	13	14	12	16
Uygur-060	10	12	13	15	19	19	11	14	9	11	14	16	13	14.2	16	16
Uygur-061	10	10	14	14	17	20	11	13	11	12	12	15	15	16	15	16
Uygur-062	12	12	14	15	19	20	11	11	11	13	12	16	13	15	15	17
Uygur-063	10	11	13	16	19	22	8	11	11	11	14	15	14	14.2	16	17.3
Uygur-064	10	11	13	16	17	21	11	12	11	13	14	15	12	15	11	16
Uygur-065	10	13	13	13	19	20	9	12	8	11	13	14	14	16	11	16
Uygur-066	12	12	15	16	17	18	10	12	9	11	15	20	12	14	16.3	17
Uygur-067	10	11	13	17	19	19	9	9	9	13	12	14	16	16.2	11	16
Uygur-068	10	12	13	13	19	22	9	10	9	14	15	17	15	15	11	14
Uygur-069	10	11	12	13	17	22	9	9	9	12	13	15	14	16	12	15
Uygur-070	12	12	13	16	17	17	8	11	11	13	12	14	14	16	15	17
Uygur-071	10	12	14	16	17	21	8	11	9	12	15	15	13	16	15	16
Uygur-072	10	12	13	13	18	22	9	9	11	12	13	15	12	15.2	11	16
Uygur-073	10	11	12	13	20	21	8	10	9	14	13	13	14	15.2	15	16
Uygur-074	11	12	13	14	17	19	10	13	12	14	15	17	15	15.2	15	16
Uygur-075	11	12	12	13	19	20	12	13	9	12	13	13	13	14	12	18
Uygur-076	10	11	12	13	15	19	10	11	9	10	12	17	13	13	15	16
Uygur-077	10	11	13	14	18	19	8	10	12	12	14	19	13	13.2	16	16
Uygur-078	10	12	13	17	19	20	9	10	12	12	13	17	13.2	14	11	15
Uygur-079	10	11	13	15	17	20	11	14	9	14	12	15	13	15.2	16	17.3
Uygur-080	10	10	15	16	17	18	9	12	9	11	13	15	12	14	15	15.3
Uygur-081	11	12	14	18	20	23	8	9	12	12	13	15	12	14	14	15
Uygur-082	10	11	12	13	18	20	9	11	12	12	14	14	14.2	15	13	13
Uygur-083	11	12	14	14	19	19	8	10	9	12	13	18	12	15.2	15	15
Uygur-084	10	12	13	14	17	18	12	13	13	13	13	18	13	13	15	16
Uygur-085	12	12	14	15	19	19	11	13	8	12	12	13	14	16.2	13	16
Uygur-086	10	12	13	15	17	22	9	11	11	13	12	16	13	15.2	13	16
Uygur-087	10	14	13	15	19	21	11	11	8	12	14	15	13	15.2	13	17
Uygur-088	11	12	13	14	18	20	8	14	12	13	13	13	14	14	11	14
Uygur-089	11	14	13	15	17	19	8	11	10	12	15	16	13	15.2	16	17
Uygur-090	12	13	13	16	18	23	10	10	12	13	12	13	13	14	8	16
Uygur-091	10	12	13	14	17	23	12	13	9	12	12	18	13	14.2	13	17.3
Uygur-092	11	12	15	16	20	22	10	11	9	12	13	15	13	16.2	11	13
Uygur-093	12	12	14	15	18	19	11	12	11	12	13	16	14	16	16	16
Uygur-094	10	11	13	14	19	21	8	14	11	14	12	15	13	15	12	16
Uygur-095	11	12	14	16	19	23	11	11	9	11	14	17	13	14	11	15
Uygur-096	10	12	13	14	17.3	22	12	12	9	12	13	17	14	15.2	11	16
Uygur-097	10	12	13	15	18	19	8	9	8	12	13	13	14	16.2	12	13
Uygur-098	10	11	13	16	17	19	9	10	11	13	17	17	14	14.2	12	16
Uygur-099	10	11	12	13	18	20	9	11	12	12	12	14	14.2	15	13	14
Uygur-100	10	10	15	16	18	19	11	12	11	12	13	19	13	14	11	15
Uygur-101	10	11	13	13	19	24	12	12	9	13	13	16	13	14	13	14
Uygur-102	10	12	13	13	17	19	8	12	9	11	13	15	13	14	11	16
Uygur-103	11	12	13	14	18	20	10	13	9	12	13	15	14	14.2	11	17
Uygur-104	11	11	17	17	19	22	9	12	11	11	14	15	15	15.2	15	16

Uygur-105	11	13	14	14	15	17.3	11	13	12	12	16	17	13	13.2	11	15
Uygur-106	11	11	14	16	17	17	8	10	9	13	13	14	15.2	16	16	16
Uygur-107	12	12	13	13	15	19	9	13	11	13	12	13	14	16.2	11	16
Uygur-108	10	12	13	16	18	20	8	8	12	12	15	18	14	14.2	11	15
Uygur-109	12	12	13	15	18	22	8	10	13	13	12	16	13	14	16	17
Uygur-110	10	11	14	14	17	21	12	13	9	9	14	14	12	13	16	18.3

Continue Supplementary Table S3

Sample ID	D21S11	D21S11	D22S1045	D22S1045	D2S1338	D2S1338	D2S441	D2S441	D3S1358	D3S1358	D5S818	D5S818	D6S1043	D6S1043	D7S820	D7S820
Hui-001	32.2	35.2	15	17	23	23	11	11	15	18	10	12	19	20	8	13
Hui-002	28	31	11	16	20	20	10	12	16	18	7	9	18	18	9	10
Hui-003	29	30	15	16	17	17	13	14	16	17	10	11	14	19	8	9.1
Hui-004	29	29	15	15	16	24	11	12	17	19	7	10	18	20	11	12
Hui-005	28	29	16	16	20	24	10	13	16	18	10	11	18	20	11	11
Hui-006	29	29	11	16	19	23	12	12	16	16	10	11	14	20	11	11
Hui-007	30	30	11	16	20	25	10	12	15	16	11	13	11	12	10	11
Hui-008	30	30	11	17	23	24	11	14	15	15	10	10	19	19	10	11
Hui-009	30	31	14	15	20	25	11	14	15	16	11	13	13	19	10	11
Hui-010	29	32.2	11	16	19	24	11	12	15	16	12	12	11	19	11	11
Hui-011	29	32.2	11	17	23	24	11.3	12	15	15	12	12	12	18	9	11
Hui-012	30	30	11	12	25	26	10	11	15	15	10	12	19	20	10	11
Hui-013	29	32.2	11	17	24	27	11	13	17	18	11	12	17	18	11	12
Hui-014	29	31	12	16	20	25	10	11	16	18	12	13	18	19	10	11
Hui-015	30	31	15	15	19	19	11	14	17	18	10	13	17	17	10	10
Hui-016	30	33.2	14	16	20	22	12	12	17	17	11	11	14	15	8	12
Hui-017	29	33.2	11	17	18	20	9.1	12	15	17	10	13	14	18	10	11
Hui-018	30	32.2	17	17	19	25	10	12	14	17	10	10	18	19	10	12
Hui-019	30	32	11	11	17	19	10	11	15	16	11	12	10	11	11	11
Hui-020	29	32.2	17	17	18	23	10	11	14	15	10	10	13	19	8	11
Hui-021	30	31	15	17	23	24	11	11	16	16	10	11	13	18	10	13
Hui-022	32	33.2	15	15	17	23	11	12	15	16	10	11	12	18	10	12
Hui-023	28	29	11	15	20	24	11	11	17	17	9	13	11	19	8	8
Hui-024	30	33.2	11	16	24	24	11	14	15	16	11	13	19	20	10	11
Hui-025	31.2	32	15	16	18	20	11	14	16	18	10	12	12	14	11	11
Hui-026	30	30	11	16	18	24	10	10	14	17	11	13	19	19	11	11
Hui-027	30	31	15	16	19	24	10	14	16	16	11	11	18	19	8	10
Hui-028	30	31	17	18	20	24	10	11	15	16	10	13	11	13	10	13
Hui-029	29	33.2	16	18	18	19	10	14	14	17	10	13	11	12	10	12
Hui-030	29	30	11	15	19	19	10	13	15	17	10	13	11	12	10	12
Hui-031	29	30	15	17	17	24	10	11.3	15	18	9	12	14	19	8	11
Hui-032	29	30	15	17	18	25	10	11	15	15	11	12	18	19	12	12
Hui-033	29	32.2	15	17	19	23	10	12	16	18	11	12	18	19	10	12
Hui-034	28.2	32.2	11	15	16	19	11	11	14	17	12	12	18	19	11	12
Hui-035	29	31	15	16	19	23	12	14	15	16	10	11	19	20	11	12
Hui-036	28	29	11	15	18	19	11	12	15	17	7	10	16	18	8	11
Hui-037	29	30	16	17	17	23	14	14	15	17	10	13	10	21	10	12
Hui-038	30	32.2	15	16	20	25	14	14	15	17	9	10	11	18	11	11
Hui-039	30	32.2	17	17	17	18	11	13	17	17	13	14	13	19	12	12
Hui-040	30	30	11	16	20	23	11	12	18	18	10	12	10	13	8	9

Hui-041	29	30	11	16	20	23	9	11.3	15	15	12	13	18	18	10	11
Hui-042	29	33.2	11	17	23	25	10	14	15	16	9	12	12	19	11	11
Hui-043	29	32.2	16	18	17	23	10	12	15	17	9	11	11	13	11	11
Hui-044	29	33.2	11	15	16	20	9.1	14	16	17	9	12	11	14	11	12
Hui-045	32.2	33.2	11	16	19	24	10	12	16	17	12	13	14	14	10	11
Hui-046	33.2	33.2	15	16	17	19	10	10	17	17	12	13	12	18	8	10
Hui-047	29	33.2	15	15	18	23	11	12	15	16	11	12	13	17	8	12
Hui-048	29	29	16	17	17	19	10	12	15	16	10	13	13	13	10	11
Hui-049	30	32	11	17	17	23	11	13	16	18	11	12	11	13	8	11
Hui-050	29	30	11	17	24	24	10	14	15	17	11	12	14	19	10	11
Hui-051	28	29	17	17	18	26	11	12	15	17	7	12	11	12	8	11
Hui-052	30	32.2	16	17	23	25	10	14	15	15	11	12	12	12	10	11
Hui-053	30	31	11	17	20	24	10	11	15	18	11	11	13	19	8	9
Hui-054	32.2	32.2	11	17	21	24	10	12	16	17	13	13	18	20	11	12
Hui-055	28	29	11	16	18	23	9.1	10	16	18	11	12	12	14	8	11
Hui-056	30	30	15	15	19	22	10	11	15	16	9	12	10	14	10	14
Hui-057	30	30	15	17	20	24	12	12	15	16	10	11	11	18	11	11
Hui-058	30	30	15	16	18	23	10	11	15	16	12	13	13	20	11	12
Hui-059	29	30	16	16	17	19	10	14	17	18	9	12	12	19	11	12
Hui-060	29	30	13	15	20	24	10	11.3	15	15	11	12	13	19	10	12
Hui-061	30	30	11	16	18	20	10	11	14	15	11	12	12	17	8	9
Hui-062	29	30	15	17	20	21	11	12	16	17	6	12	14	18	10	12
Hui-063	30	30	15	15	19	24	10	12	15	16	11	12	12	16	8	11
Hui-064	29	30	15	16	18	23	11	11	16	17	10	13	13	13	11	12
Hui-065	28	30	15	16	18	24	11	11	15	15	10	11	20	21	9	11
Hui-066	32.2	33.2	11	16	20	23	10	11	16	17	11	11	10	13	11	12
Hui-067	29	30	11	16	22	26	10	14	15	17	11	13	12	14	10	11
Hui-068	30	30	16	17	16	25	10	11	14	16	7	11	13	18	12	12
Hui-069	29	33.2	15	17	18	23	11	14	16	18	11	12	14	14	9	11
Hui-070	29	29	16	17	20	20	11	12	14	15	9	11	18	19	11	11
Hui-071	31	35.2	11	15	18	23	10	14	15	15	9	11	13	14	10	10
Hui-072	29	30	17	17	19	26	10	11.3	17	17	9	12	13	20	11	11
Hui-073	30.2	30.2	11	17	24	26	10	12	15	16	11	12	19	22.3	11	12
Hui-074	29	30	15	16	23	26	11	12	15	17	12	13	13	14	11	11
Hui-075	31	33.2	11	15	17	22	11	11	15	18	11	13	11	13	11	11
Hui-076	30	32.2	11	17	19	20	11	11	15	15	12	12	13	19	8	11
Hui-077	28	32.2	11	16	17	23	11	12	16	17	12	12	12	13	9	11
Hui-078	29	32.2	11	11	18	23	10	14	15	18	11	12	13	14	8	8
Hui-079	30	32.2	15	16	18	23	11	12	16	17	9	11	18	18	11	11
Hui-080	30	32.2	11	17	18	20	12	14	15	17	12	12	10	18	11	12
Hui-081	29	30	15	18	19	20	11.3	12	14	17	10	11	18	18	10	12
Hui-082	32.2	32.2	11	16	18	24	10	14	15	17	9	9	11	18	11	12
Hui-083	29	29	11	15	18	22	10	12	16	16	11	12	12	17	11	12
Hui-084	30	32.2	15	15	17	20	11	12	15	16	12	13	14	19	10	11
Hui-085	30	31.2	15	16	19	23	10	11	16	17	11	12	11	18	10	11
Hui-086	29	29	11	17	17	24	10	12	15	18	11	12	10	13	7	12
Hui-087	29	30	11	17	20	25	10	12	15	17	10	13	10	13	8	12
Hui-088	29	30	11	15	18	19	9.1	12	15	15	10	12	10	12	8	11
Hui-089	30	32	11	16	18	25	14	14	15	15	10	13	11	12	8	11

Hui-090	29	30	16	17	24	25	10	14	17	18	7	11	10	14	11	11
Hui-091	30	30	15	17	19	24	11	11.3	16	17	10	11	12	18	11	11
Hui-092	30	32.2	16	16	24	24	10	15	15	17	10	13	11	14	10	11
Hui-093	30	31	15	17	23	23	10	10	14	15	11	13	11	12	8	8
Hui-094	30	31	15	15	19	25	10	11	15	16	11	12	13	19	8	11
Hui-095	30	30.3	11	15	23	24	10	11	16	16	11	11	13	18	8	12
Hui-096	28.2	31.2	11	15	18	24	11	12	15	15	11	11	11	14	10	10
Hui-097	29	32.2	17	17	21	23	11	13	15	17	11	13	19	20	8	12
Hui-098	28	28	11	11	19	23	10	12	16	16	10	13	13	18	11	12
Hui-099	32.2	34.2	11	15	19	23	11	11	15	17	12	13	11	14	10	11
Hui-100	30	30	11	16	22	23	11	12	17	18	12	13	9	19	10	11
Hui-101	30.2	31.2	16	17	23	24	9.1	11	16	17	9	10	11	17	11	11
Hui-102	29	30	16	16	23	24	11	11.3	16	16	11	13	18	20	10	12
Hui-103	29	30	16	17	19	24	10	14	16	18	7	14	13	18	8	12
Hui-104	29	31.2	11	17	24	25	11	11	18	18	7	13	13	18	11	11
Hui-105	29	29	15	18	24	24	12	14	15	16	10	10	12	13	10	12
Hui-106	30	31	11	15	23	23	10	12	14	15	13	13	11	13	10	11
Hui-107	30	33.2	11	11	18	26	10	12	15	16	12	13	13	20	11	12
Hui-108	29	30	11	15	19	23	10	12	15	16	10	11	12	14	10	11
Hui-109	32.2	32.2	15	16	19	24	11	14	14	15	11	12	13	19	10	11
Hui-110	29	32.2	16	17	19	23	12	15	16	17	11	12	12	20	8	12
Hui-111	32.2	33.2	17	17	18	23	11	11	14	18	11	13	13	19	10	11
Hui-112	30	30.2	15	17	22	24	10	11	16	18	9	12	17.3	18	10	12
Hui-113	29	31	16	16	19	23	10	11.3	17	17	11	11	14	20	11	12
Hui-114	30	31	11	16	19	24	10	11	15	16	11	13	17	19	8	11
Hui-115	30	31	11	11	23	23	14	14	15	17	13	13	13	14	9.1	12
Hui-116	31.2	33.2	15	16	19	23	11	11	16	17	11	12	12	14	10	11
Hui-117	31	31.2	16	16	19	24	10	11	15	17	11	11	14	14	9	12
Hui-118	30	32.2	11	15	23	25	11	15	16	17	10	11	12	18	12	12
Hui-119	29	31	15	17	19	23	11	12	15	18	10	12	18	20	10	11
Hui-120	29	30	11	15	17	18	12	12	16	18	9	10	11	14	8	12
Hui-121	30.3	31.2	11	14	18	24	10	11	15	16	11	12	12	19	9	12
Hui-122	29	29.2	11	15	23	23	11	14	14	15	10	12	10	18	8	8
Hui-123	30	30	15	15	20	25	11	14	15	15	13	13	18	19	10	12
Hui-124	28	29	15	15	23	27	11	11.3	15	18	11	12	11	14	8	10
Hui-125	29	32.2	16	16	23	24	11	11	17	18	12	12	11	14	11	12
Hui-126	27	29	15	16	20	21	10	14	16	17	12	13	14	19	8	8
Hui-127	30	31.2	16	17	18	24	10	11	14	16	11	13	12	13	8	11
Hui-128	29	31.2	11	16	17	18	10	12	15	16	10	12	14	19	8	9.1
Hui-129	28	29	16	17	17	25	12	12	16	16	10	11	18	21	8	13
Hui-130	29	30	16	16	17	24	10	10	16	16	10	13	13	18.2	10	11
Hui-131	30	31	11	15	19	19	10	11	16	17	12	12	18	18	10	11
Hui-132	31	31.2	11	11	19	25	11.3	13	16	17	9	11	17	18	10	12
Hui-133	29	32.2	11	16	17	23	10	11	16	17	12	13	11	20	8	12
Hui-134	30	31	16	17	17	24	11	12	16	16	11	12	15	18	10	12
Hui-135	30	30.2	11	15	20	23	11	11	15	16	12	12	11	12	10	12
Hui-136	32.2	33.2	11	11	19	25	11	11	15	16	10	12	12	13	10	11
Hui-137	29	32.2	16	17	18	24	11	12	15	18	11	14	13	18	8	10
Hui-138	28.2	31	11	16	19	20	10	11	18	18	10	12	11	17	11	12

Hui-139	31	31.2	11	15	17	18	11	11	15	15	11	13	14	19	8	10
Hui-140	29	30.2	11	17	18	26	9	12	14	17	10	12	19	19	8	9
Hui-141	30	31	17	17	18	23	10	11	15	16	9	10	18	19	11	12
Hui-142	30	31.2	11	15	23	23	11	12	16	16	10	12	13	14	11	11
Hui-143	31	32	11	15	19	19	10	11	16	16	12	14	18	19	8	12
Hui-144	31.2	32.2	15	15	17	24	10	12	15	16	9	13	13	18	10	11
Hui-145	29	30	15	16	23	23	11.3	12	15	15	10	11	18	19	11	12
Hui-146	30	30	15	16	22	25	10	11	15	15	11	11	14	18	8	11
Hui-147	30	31.2	11	15	18	20	10	11	16	17	10	13	13	18	8	12
Hui-148	30	32.2	16	17	23	24	10	14	15	16	12	13	11	14	9	9
Hui-149	28	32.2	16	17	20	24	10	12	15	18	11	13	14	19	9	12
Hui-150	31.2	31.2	16	16	18	20	11	12	15	16	12	12	14	18	8	11
Hui-151	31	31.2	16	17	19	20	11	11	15	18	10	11	19	20	8	11
Hui-152	30	33.2	11	18	20	25	11	11	15	17	11	11	13	18	12	12
Hui-153	29	33.2	15	15	17	20	10	11	16	16	11	12	11	12	11	12
Hui-154	30	33.2	15	16	18	23	10	10	15	16	10	13	11	18	12	12
Hui-155	29	33.2	11	15	18	19	12	12	15	18	12	13	11	18	8	10
Hui-156	28	31	11	16	18	24	10	10	16	17	13	14	18	18	8	10
Hui-157	30	31	16	16	24	25	10	14	16	17	11	13	11	19	8	11
Hui-158	31	33.2	15	17	19	20	10	10	15	15	10	12	12	14	12	13
Hui-159	30.2	32.2	15	17	22	24	11	14	14	15	12	12	18	19	11	11
Hui-160	32	32.2	16	17	18	19	11	11.3	16	16	11	13	18	20	8	10
Hui-161	29	30	16	17	20	24	9.1	11	16	16	9	12	19	19	8	8
Hui-162	30	31.2	15	15	19	24	10	12	14	15	11	13	14	18	11	12
Hui-163	29	33	15	15	17	23	10	12	15	17	11	12	18	19	11	11
Hui-164	29	30	11	17	23	24	14	14	16	18	10	12	12	13	9	11
Hui-165	29	30	16	16	24	24	11	12	15	16	11	12	11	18	8	8
Hui-166	30	32.2	11	15	23	24	11	12	15	17	11	12	12	18	12	13
Hui-167	29	30	15	15	17	19	11	13	16	16	11	12	13	13	11	11
Hui-168	32.2	32.2	17	17	23	23	10	12	16	16	7	10	12	18	12	12
Hui-169	28	31.2	17	17	22	23	11	14	16	16	11	13	19	20	10	11
Hui-170	30.2	31.2	11	15	17	26	11	11	14	15	10	12	12	12	9	10
Hui-171	29	30	16	16	20	23	10	14	15	16	10	11	9	12	8	11
Hui-172	29	30.2	16	17	23	24	10	14	14	16	10	11	19	19	9	9
Hui-173	29	29	11	15	23	23	11	14	17	19	11	13	11	18	8	11
Hui-174	28	30	15	18	22	25	10	11	15	15	11	12	18	18.2	8	11
Hui-175	31.2	32.2	15	17	17	18	11	12	15	17	11	12	13	17	9	11
Hui-176	29	30	11	11	20	23	10	11	15	16	9	11	11	19	8	11
Hui-177	29	31.2	11	15	21	24	11	12	15	18	12	13	13	18	8	9
Hui-178	31.2	33.2	11	16	25	26	9.1	10	18	18	11	11	11	13	11	11
Hui-179	28	29	11	16	20	20	11	11	16	17	11	12	12	18	11	12
Hui-180	30	32.2	15	15	19	23	10	11	15	15	10	12	12	14	8	11
Hui-181	32.2	32.2	15	16	17	24	14	14	15	17	10	12	12	18	10	11
Hui-182	29	30	11	17	18	24	11	11	15	16	10	10	12	19	10	10
Hui-183	28	30	11	15	19	23	12	13	15	17	9	11	18	20	8	10
Tibetan-001	29	29	11	19	18	19	10	11	15	15	10	12	13	19	11	12
Tibetan-002	30	32	17	19	19	23	10	11	16	17	10	10	18	19	12	12
Tibetan-003	29	31	11	17	20	20	10	10	15	15	10	10	12	17	11	12
Tibetan-004	29	30	11	11	20	23	10	10	17	17	10	11	14	18	11	11

Tibetan-005	31.2	33.2	11	17	23	23	12	14	15	16	11	11	18	19	11	14
Tibetan-006	30	31	11	16	23	24	10	10	15	17	11	13	18	19	12	12
Tibetan-007	31	32.2	15	17	17	19	10	12	15	16	10	11	18	18	10	11
Tibetan-008	30	30	11	11	19	24	10	11	17	19	9	12	11	18	11	11
Tibetan-009	29	30	16	17	20	25	11	15	16	17	9	9	13	19	12	13
Tibetan-010	30	30	11	17	20	25	10	12	17	18	10	10	12	18	10	11
Tibetan-011	29	30	11	11	23	23	10	12	16	17	11	11	12	13	12	12
Tibetan-012	29	31	11	16	19	20	10	11	16	17	11	13	11	16	8	12
Tibetan-013	29	33.2	15	17	19	24	10	10	15	18	10	10	15	20.3	8	11
Tibetan-014	31	32.2	11	16	23	25	11	11	15	18	10	10	12	20	12	13
Tibetan-015	30	30	15	17	23	23	10	11	17	17	11	12	10	17	9	12
Tibetan-016	29	30	11	17	19	20	11	12	15	15	9	12	11	11	10	11
Tibetan-017	29	29	17	17	18	20	11	12	15	15	9	12	10	11	8	10
Tibetan-018	29	31	15	16	21	24	11.3	13	16	17	11	11	10	18	11	11
Tibetan-019	31	32.2	16	17	21	21	10	10	15	18	10	10	18	19	8	11
Tibetan-020	31	31	15	17	18	19	11	14	13	16	12	13	12	20	10	12
Tibetan-021	29	32.2	11	15	19	19	12	14	17	17	10	12	19	21.3	11	11
Tibetan-022	31	32	11	16	23	26	12	14	15	16	8	10	14	16	11	13
Tibetan-023	27	32.2	11	18	21	23	11	12	15	15	11	11	13	20.3	8	11
Tibetan-024	29	33.2	15	17	19	25	10	14	15	16	11	13	11	14	11	12
Tibetan-025	27	29	15	18	21	24	12	12	15	15	11	11	13	19	8	13
Tibetan-026	30	30	15	18	20	23	10	10	16	17	11	12	17	20.3	10	11
Tibetan-027	29	32.2	11	15	19	19	12	14	16	17	11	12	12	13	8	11
Tibetan-028	29	30	16	16	22	22	11	11	15	17	11	11	17	18	8	11
Tibetan-029	29	29	16	17	23	25	11	12	15	17	9	11	12	18	12	13
Tibetan-030	28	30	16	16	19	19	11	11	15	15	10	10	12	19	10	11
Tibetan-031	31.2	32.2	16	16	20	23	10	10	15	16	12	12	12	19	11	12
Tibetan-032	29	31.2	11	15	19	23	10	14	15	16	12	13	18	19	12	12
Tibetan-033	30.2	31.2	17	17	23	25	10	10	15	16	11	11	15	19	10	12
Tibetan-034	30	31	14	15	19	24	10	14	16	18	12	13	12	21.3	10	10
Tibetan-035	30	32.2	11	16	19	24	11	14	16	16	10	13	11	20.3	11	12
Tibetan-036	29	31.2	11	16	20	22	11.3	14	16	16	10	11	12	19	8	10
Tibetan-037	30	30	11	11	17	19	10	12	15	16	10	12	13	14	11	13
Tibetan-038	30	30	15	15	19	23	10	13	16	16	10	12	13	19	11	11
Tibetan-039	30	31.2	11	15	20	21	11	12	17	17	10	12	13	20	11	11
Tibetan-040	31.2	32.2	11	11	19	23	10	11	16	16	11	13	11	19	10	12
Tibetan-041	28	32.2	11	17	20	23	10	11	15	17	11	12	12	19	11	12
Tibetan-042	33.2	33.2	16	17	20	23	11	12	15	16	10	12	18	18	8	12
Tibetan-043	31	31.2	15	15	19	23	10	12	16	17	11	12	11	11	12	12
Tibetan-044	30	31	11	17	19	23	11	14	16	17	11	13	20	20	12	12
Tibetan-045	29	31.2	11	17	19	23	10	10	16	16	10	10	18	21.3	8	14
Tibetan-046	30	31	15	18	20	23	10	14	16	17	11	12	11	12	11	12
Tibetan-047	29	31	11	16	21	21	12	13	15	16	13	13	13	18	11	12
Tibetan-048	29	32.2	16	17	18	23	11	11	16	17	10	12	11	19	9	10
Tibetan-049	30	32	11	15	18	24	11	12	17	18	11	12	13	17	10	11
Tibetan-050	29	29	11	11	18	20	10	11	15	17	11	11	13	18	8	12
Tibetan-051	31	32.2	11	11	19	23	11	14	15	16	10	11	18	20.3	11	13
Tibetan-052	29	31.2	11	16	19	23	11	12	15	17	11	11	10	11	8	12
Tibetan-053	30	31	11	16	17	24	10	10	15	17	10	11	14	18	8	11

Tibetan-054	29	32.2	11	17	19	23	10	11	16	17	11	12	11	18	8	10
Tibetan-055	29	32	15	17	20	20	11	14	15	16	11	11	18	21.3	10	11
Tibetan-056	29	29	11	11	20	23	11	12	15	16	11	12	12	20	11	13
Tibetan-057	30	31	15	16	18	24	10	14	15	15	12	12	19	19	9	13
Tibetan-058	29	31	11	18	19	24	11	12	15	15	10	11	13	20.3	8	13
Tibetan-059	31	32.2	11	15	20	20	10	11	15	17	10	10	18	18	9	12
Tibetan-060	29	32.2	15	17	23	23	10	12	15	16	10	11	11	11	12	13
Tibetan-061	28	31.2	14	17	19	20	10	11	15	17	10	10	13	17	11	12
Tibetan-062	29	32.2	11	11	19	20	10	14	15	17	11	13	18	20	11	12
Tibetan-063	29	31.2	15	16	20	25	10	10	16	16	12	12	18	18	8	12
Tibetan-064	30	33.2	11	15	20	23	11	14	16	17	12	12	11	18	11	11
Tibetan-065	30	30	15	15	20	23	10	11	15	16	10	12	17	18	8	11
Tibetan-066	30	33.2	16	16	23	24	10	14	15	18	10	11	12	12	11	12
Tibetan-067	30	30.2	11	18	18	20	11	12	17	18	11	11	18	18	8	11
Tibetan-068	29	29	17	18	24	25	11	11	16	17	11	11	13	15	10	10
Tibetan-069	29	33.2	15	17	21	24	11	11.3	16	17	10	12	12	18	8	9
Tibetan-070	31	32.2	11	11	22	24	10	11	15	16	10	13	10	14	8	11
Tibetan-071	31	31	11	16	18	20	11	13	15	15	12	13	14	21	12	12
Tibetan-072	30.2	31	15	17	17	20	10	11	15	17	11	13	11	14	10	11
Tibetan-073	30	30	11	15	16	23	10	11	17	17	10	11	12	20	8	13
Tibetan-074	29	29	15	17	18	25	11	11	15	16	11	13	18	18	11	12
Tibetan-075	29	30	11	17	19	20	10	14	15	15	10	12	10	18	10	12
Tibetan-076	30	33.2	11	17	18	21	12	12	16	17	10	12	12	14	9	11
Tibetan-077	29	30	17	17	19	24	10	14	15	16	11	12	18	19	10	11
Tibetan-078	30	32	16	16	23	23	10	11	15	17	10	11	11	18	10	12
Tibetan-079	29	30	11	15	20	22	10	14	16	16	11	11	13	18	10	11
Tibetan-080	30	31	16	17	20	21	10	11	15	17	11	13	11	17	8	12
Tibetan-081	29	30	15	15	19	23	10	11	15	17	12	12	12	19	10	11
Tibetan-082	29	31	17	17	23	24	10	11	15	17	10	12	18	20	8	12
Tibetan-083	30.2	32.2	17	17	18	23	11	11	14	15	10	11	11	13	12	12
Tibetan-084	30.2	32.2	15	17	20	24	10	11	15	17	8	11	10	18	8	10
Tibetan-085	30	31	11	15	23	24	10	14	16	16	12	13	11	12	11	12
Tibetan-086	29	29	16	16	21	24	12	13	15	15	12	13	11	13	11	13
Tibetan-087	29	30	15	18	19	24	10	14	15	16	9	12	14	14	11	12
Tibetan-088	30	33.2	15	18	23	24	11	14	17	17	11	12	17	20	8	9
Tibetan-089	30	31	11	11	19	20	11	15	15	16	12	12	14	18	11	11
Tibetan-090	29	30	16	17	23	24	10	14	15	17	11	13	18	19	10	11
Tibetan-091	29	31.2	11	16	18	23	11	12	15	16	10	11	13	19	11	12
Tibetan-092	30	30	15	17	19	20	10	11	15	15	10	10	19	20	8	11
Tibetan-093	28.2	32.2	11	18	20	24	11	12	15	15	10	13	18	18	12	12
Tibetan-094	30	32	16	17	20	23	10	11	15	17	9	11	11	14	8	12
Tibetan-095	29	29	11	11	21	22	10	10	15	15	11	11	14	17	11	11
Tibetan-096	33.2	33.2	11	11	18	20	11	11	15	16	9	11	18	18	11	13
Tibetan-097	29	30	11	17	20	20	12	14	15	16	10	13	12	21.3	11	12
Tibetan-098	29	32.2	11	16	20	24	10	11	17	17	12	13	10	17	8	12
Tibetan-099	31	31.2	16	17	20	25	10	12	16	16	11	13	11	14	10.1	12
Tibetan-100	29	30	11	15	24	25	10	10	16	16	11	11	12	13	11	11
Tibetan-101	31	32.2	15	15	19	24	12	14	15	16	11	11	12	18	8	12
Tibetan-102	29	31	15	17	22	25	10	11	16	17	10	13	18	18	10	11

Tibetan-103	29	30	16	16	19	23	10	14	16	17	11	11	10	15	8	10
Tibetan-104	30	30.2	11	15	23	23	11	13	16	17	11	12	13	14	9	12
Tibetan-105	29	30	11	16	19	24	12	13	16	17	11	11	14	21	10	11
Tibetan-106	29	29	11	11	18	20	11	14	16	17	11	11	13	18	8	10
Tibetan-107	29	30	16	16	23	25	10	12	16	17	10	11	12	21.3	9	11
Tibetan-108	29	31.2	11	16	17	19	10	12	16	17	10	10	18	18	11	12
Tibetan-109	30	31	11	17	18	20	10	14	15	16	10	10	15	19	8	12
Tibetan-110	29	29	15	15	18	25	12	12	15	15	11	11	10	15	8	11
Tibetan-111	29	30	11	15	20	20	10	13	16	18	11	13	12	18	11	11
Tibetan-112	30	31	11	16	23	23	12	14	15	16	11	13	10	18	10	11
Tibetan-113	29	31	11	16	19	23	11	11	16	17	10	11	13	18	11	11
Tibetan-114	29	31.2	11	11	23	23	12	12	18	18	10	11	13	19	9	9
Tibetan-115	30	33.2	11	17	20	20	10	11	16	16	10	14	9	12	8	11
Tibetan-116	30	32.2	11	11	17	22	10	10	15	15	11	11	11	19	10	12
Tibetan-117	29	33.2	15	16	18	24	10	15	17	17	10	12	14	20	11	11
Tibetan-118	28	29	15	17	23	23	11.3	13	15	15	9	10	12	19	11	12
Tibetan-119	29	33.2	15	16	16	23	14	14	15	15	10	11	10	11	10	11
Tibetan-120	30	33.2	11	16	20	23	10	14	16	16	11	11	9	18	8	8
Tibetan-121	30	31	11	17	19	20	11	11	16	18	9	13	11	17	11	11
Tibetan-122	32.2	33.2	11	16	18	20	11	11	15	19	12	12	13	19	12	12
Tibetan-123	30	30	16	17	19	23	10	13	17	17	10	12	11	12	9	12
Tibetan-124	29	29	15	17	18	23	11	12	15	17	10	12	14	19	11	11
Tibetan-125	30	31.2	11	17	21	25	10	10	15	16	11	13	12	13	11	12
Tibetan-126	30	32.2	16	16	20	20	11	12	15	16	11	13	12	12	8	11
Tibetan-127	29	31	11	11	20	23	11	11	15	18	10	13	11	14	12	12
Tibetan-128	30	31	15	15	17	20	11	14	14	15	11	11	19	19	10	12
Tibetan-129	29	31.2	15	16	19	23	10	10	16	18	11	13	13	19	10	12
Tibetan-130	30	31	11	14	19	20	10	15	15	16	11	15	18	20	8	8
Tibetan-131	29	29	16	16	19	24	10	11	15	17	11	11	17	19	11	12
Tibetan-132	32.2	32.2	11	17	23	25	10	10	16	18	10	12	18	19	8	12
Tibetan-133	30	31	11	15	19	23	11	11	15	16	11	12	11	12	10.1	11
Tibetan-134	29	29	15	15	20	25	10	13	15	16	11	11	18	19	10	11
Tibetan-135	29	29	11	15	19	19	11	12	15	16	11	12	12	12	8	12
Tibetan-136	29	32	16	17	19	24	11	11	16	18	11	12	13	20	10	11
Tibetan-137	29	32.2	11	18	20	22	11	12	17	17	10	11	10	11	8	8
Tibetan-138	28	30	15	16	20	20	10	13	16	17	9	11	13	20	11	12
Tibetan-139	31.2	33.2	11	11	19	20	10	14	16	18	11	12	13	18	8	11
Tibetan-140	30	31.2	11	11	20	25	11	12	16	16	12	12	12	13	11	11
Tibetan-141	29	31.2	16	16	18	18	10	13	15	16	10	11	11	13	10	11
Tibetan-142	29	31	11	11	18	25	10	11	16	17	11	12	13	13	11	11
Tibetan-143	29	30	11	11	18	23	10	10	17	17	9	11	10	18	9	11
Tibetan-144	30	31.2	15	17	20	25	10	14	15	15	12	12	8	11	8	10
Tibetan-145	30	32.2	15	17	22	23	11	13	15	15	10	11	19	19	8	9
Tibetan-146	31	32.2	11	15	20	20	10	11	15	15	9	12	18	20.3	12	12
Tibetan-147	28	30	15	16	19	20	10	15	17	17	11	12	12	13	11	11
Tibetan-148	28.2	29	16	16	19	23	10	14	17	17	10	11	13	19	8	8
Tibetan-149	29	32.2	11	19	19	24	11	12	15	15	10	12	19	20	11	11
Tibetan-150	31	31.2	16	17	17	22	10	11	15	16	11	13	11	20	8	11
Tibetan-151	32.2	33.2	16	17	20	24	12	14	13.2	17	12	12	13	19	10	11

Tibetan-152	29	30	15	16	20	23	12	14	15	17	10	13	12	14	11	12
Tibetan-153	32.2	33.2	11	16	20	23	10	10	15	16	10	10	17	19	8	10
Tibetan-154	30	32.2	11	14	19	23	11	14	16	18	12	12	10	18	11	12
Tibetan-155	29	30	11	11	18	24	11	11	15	15	11	11	10	18	9	11
Tibetan-156	28.2	29	15	17	23	24	10	14	15	16	11	13	11	19	8	11
Tibetan-157	29	32.2	16	17	18	24	12	12	15	16	10	11	12	14	11	12
Tibetan-158	29	29	11	16	20	24	12	14	16	17	9	13	13	18	11	12
Tibetan-159	29	32	17	17	17	23	10	14	16	16	11	11	14	19.3	11	12
Tibetan-160	29	30	11	16	20	23	12	14	16	17	10	11	13	18	9	11
Tibetan-161	28.2	29	11	16	23	24	11	11	15	16	11	13	8	14	10	12
Tibetan-162	30	31	11	15	19	19	11	13	16	16	11	12	18	20	8	10
Tibetan-163	30	32.2	15	17	19	19	11	13	15	15	12	12	11	13	8	12
Tibetan-164	31	31	11	15	20	23	10	10	16	17	11	12	11	19	11	13
Tibetan-165	30	31.2	11	17	19	23	10	11.3	15	19	10	11	14	18	9	10
Tibetan-166	29	29.2	11	15	17	20	10	14	16	18	13	13	11	13	11	11
Tibetan-167	29	29	15	17	19	25	11	14	15	16	11	12	14	20	10	12
Tibetan-168	29	32	17	17	17	23	11	12	15	16	11	11	12	14	8	12
Tibetan-169	31	31.2	17	17	19	21	11	12	16	17	11	13	17	19	8	13
Tibetan-170	29.2	33.2	11	15	19	24	10	12	15	15	11	11	18	18	8	10
Tibetan-171	30	32.2	11	17	19	19	11	11	15	16	10	12	12	18	11	12
Tibetan-172	28	32	15	16	19	24	12	12	14	16	11	12	11	16	11	11
Tibetan-173	29	32.2	16	16	19	25	10	12	16	16	10	11	11	19	12	12
Tibetan-174	30	31.2	14	16	18	19	14	15	15	17	10	11	17	19	12	13
Tibetan-175	30	32.2	11	11	19	23	10	14	15	15	10	10	18	21.3	8	12
Tibetan-176	30	31	17	17	19	19	11	14	16	18	9	13	15	18	8	11
Tibetan-177	30	33	16	16	19	25	11	12	16	17	8	9	14	14	11	11
Tibetan-178	29	31.2	11	17	19	20	11	12	16	18	11	11	14	20	9	12
Tibetan-179	29	29	15	16	19	23	12	12	15	17	11	13	11	11	12	12
Tibetan-180	30	32	17	17	20	24	10	10	16	16	12	13	13	14	10	13
Tibetan-181	30	32.2	16	16	17	24	10	11	16	16	11	11	11	18	8	8
Tibetan-182	30	31.2	17	19	19	19	10	11	17	18	11	14	12	21	8	13
Tibetan-183	29	32.2	11	17	23	23	11	11	16	16	10	10	17	18	11	12
Tibetan-184	29	31.2	16	16	18	23	10	11	15	18	11	14	18	19	11	12
Tibetan-185	29	30	16	17	20	20	11	12	15	17	9	11	11	18	10	11
Tibetan-186	29	31	15	17	20	23	10	12	15	15	11	11	11	14	9	11
Tibetan-187	30.2	33.2	16	16	17	20	10	14	14	16	13	13	13	13	11	12
Tibetan-188	30	32.2	11	15	20	23	10	10	15	16	12	15	14	14	12	12
Tibetan-189	29	31	11	15	20	23	11	12	15	17	10	10	11	13	10	12
Tibetan-190	31	31	16	17	20	23	10	11	16	17	11	13	11	13	8	11
Tibetan-191	29	30	16	16	17	20	10	11	15	17	10	10	13	14	10	11
Tibetan-192	28	32.2	16	16	19	20	11.3	15	16	17	11	12	12	19	11	11
Tibetan-193	28	30	11	16	20	22	12	12	15	16	10	11	12	13	12	13
Tibetan-194	31	31	11	18	23	24	11	15	15	17	11	13	14	15	11	12
Tibetan-195	31	33.2	15	16	23	24	10	14	16	17	9	13	11	19	9	12
Tibetan-196	29	29	15	15	17	23	9.1	10	14	16	11	11	18	19	12	13
Tibetan-197	29	30	11	15	19	21	12	12	16	17	10	13	10	19	9	12
Tibetan-198	30	32.2	11	15	23	23	10	12	16	17	10	15	12	19	10	11
Tibetan-199	29	33.2	15	15	21	24	11	12	17	17	9	11	12	21.3	11	12
Tibetan-200	29	29	16	16	18	24	10	14	16	17	12	13	14	18	11	11

Uygur-001	28.2	30.2	11	14	21	23	10	10	16	17	12	12	10	18	10	12
Uygur-002	30	30	11	15	17	17	10	12	16	17	10	11	18	19	9	13
Uygur-003	29	30	11	15	19	23	10	11	17	18	10	11	13	18	9	10
Uygur-004	30	30	15	19	23	24	11	11	17	18	11	12	11	13	8	8
Uygur-005	30	30	11	15	18	18	10	11	16	17	10	12	11	17	10	10
Uygur-006	29	30	17	19	19	23	11	11	15	17	11	12	11	19	8	11
Uygur-007	30	30	11	15	18	19	10	10	15	16	11	11	11	18	11	11
Uygur-008	30	33.2	11	15	18	18	9.1	10	16	17	10	11	19	19	8	13
Uygur-009	30	32.2	11	17	17	23	11	14	16	17	11	12	10	12	8	13
Uygur-010	29	30	16	16	17	17	10	11	15	15	12	13	18	21	11	11
Uygur-011	28	28	16	17	18	23	11	11	16	17	11	11	11	12	10	10
Uygur-012	30	31.2	16	16	17	19	9.1	11	15	18	10	12	11	11	10	10
Uygur-013	28	33.2	16	17	17	24	11	11	15	17	10	11	18	20	8	12
Uygur-014	29	33.2	11	11	19	19	15	15	15	17	11	11	11	20	8	9
Uygur-015	30	31.2	11	11	20	24	11	14	15	16	11	13	11	14	9	11
Uygur-016	29	30	11	16	17	19	11	13	15	15	12	13	11	12	8	13
Uygur-017	28	29	14	17	22	24	11	14	16	17	11	11	12	17	10	11
Uygur-018	30	30	16	16	19	20	11	14	16	17	10	11	18	18	9	12
Uygur-019	32.2	33.2	16	17	17	23	10	11	15	18	10	11	14	20	10	11
Uygur-020	30	30	15	16	19	23	14	15	15	17	11	12	12	14	11	12
Uygur-021	29	30	11	17	18	23	11	11	17	17	11	11	11	18	10	11
Uygur-022	30	33.2	11	16	23	24	11	14	17	18	11	12	11	12	8	11
Uygur-023	28	28	15	16	20	25	11	11	15	16	9	11	12	13	9	12
Uygur-024	30	31.2	11	16	18	23	11	14	16	16	12	12	11	17	10	11
Uygur-025	30	31	17	17	17	21	11	15	15	17	11	13	12	13	9	13
Uygur-026	30	31.2	11	16	17	18	10	13	15	17	10	11	15	17	11	13
Uygur-027	29	35.2	11	16	18	23	14	14	16	18	13	13	18	19	10	11
Uygur-028	30	30	16	17	18	18	10	14	15	18	13	13	11	18	8	10
Uygur-029	30	30	17	17	23	25	9.1	11	18	18	10	12	12	12	8	11
Uygur-030	29	30	11	16	19	25	11	14	16	18	11	13	18	20	8	8
Uygur-031	29	29	11	14	18	19	10	15	15	17	12	13	11	17	8	13
Uygur-032	30	32.2	11	15	19	23	11	11	17	17	11	12	17	18	8	11
Uygur-033	28	29	11	16	18	19	11	11	16	18	13	13	10	13	8	8
Uygur-034	29	31	11	16	19	22	11	12	16	17	10	10	11	20	8	12
Uygur-035	28	30	11	15	18	22	10	12	15	17	9	13	11	11	10	11
Uygur-036	29	29	16	16	17	18	11	11	15	16	11	13	11	18	10	12
Uygur-037	30	33.2	16	17	18	20	11	12	15	18	11	12	11	20	10	10
Uygur-038	31	32.2	11	11	18	18	11	14	15	15	12	13	13	20	9	11
Uygur-039	30	30.2	11	17	19	25	10	12	15	18	9	12	12	19	8	12
Uygur-040	29	31.2	14	15	18	23	10	11	18	18	10	11	18	21	9	11
Uygur-041	29	30	11	15	19	23	9	11	15	18	11	13	12	15	9	9
Uygur-042	29	30	15	17	18	25	10	12	18	18	13	13	12	19	8	10
Uygur-043	30	33.2	11	16	17	20	10	13	17	17	11	12	11	18	12	12
Uygur-044	29	32.2	16	17	17	19	9.1	15	15	16	11	12	10	20	12	12
Uygur-045	30	31.2	11	16	18	23	11	11	15	16	11	13	11	17	11	11
Uygur-046	30	30	15	16	22	24	11	11.3	16	17	10	11	11	18	10	12
Uygur-047	28.2	32.2	15	16	20	25	11	11	15	15	11	13	11	12	10	10
Uygur-048	30	31.2	15	15	19	20	11	11	14	18	9	11	11	11	9	9
Uygur-049	31.2	32.2	14	16	23	24	10	11	16	16	10	13	18	18	8	11

Uygur-050	29	31.2	16	17	20	23	11	11.3	15	15	11	12	18	18	10	10
Uygur-051	29	30	16	17	19	25	10	10	15	16	9	10	11	12	8	11
Uygur-052	28	28.2	16	17	20	24	14	15	16	17	13	13	11	18	8	12
Uygur-053	29	29	15	16	18	23	14	15	16	17	13	13	11	11	8	8
Uygur-054	31	31.2	11	17	17	18	12	12	16	17	12	13	11	20	10	12
Uygur-055	31.2	32.2	11	17	20	25	11.3	12	15	16	10	11	10	14	8	8
Uygur-056	29	32.2	11	17	23	24	11	11.3	15	16	12	12	18	18	10	11
Uygur-057	29	30.2	15	16	18	18	10	10	15	18	10	11	12	19	10	13
Uygur-058	29	29	11	11	18	24	10	11	15	17	11	12	18	20	8	13
Uygur-059	30	30	16	17	17	19	10	14	15	16	12	13	11	15	10	11
Uygur-060	30	32.2	15	16	20	20	11	11	15	17	12	14	17	20	11	11
Uygur-061	29	30	11	16	18	20	9.1	11	16	17	11	12	11	11	10	11
Uygur-062	30	32.2	16	17	19	21	10	10	17	18	9	11	11	20	8	11
Uygur-063	30	31	15	15	17	17	10	11	15	16	11	13	14	18	8	11
Uygur-064	31	32.2	15	15	17	17	11	12	15	16	11	13	14	14	11	12
Uygur-065	32.2	32.2	15	16	19	21	11	11	16	17	11	12	11	17	12	12
Uygur-066	32.2	32.2	15	16	23	24	10	14	16	17	9	12	11	18	9	11
Uygur-067	32.2	33.2	11	16	17	23	10	12	15	15	12	12	12	18	11	11
Uygur-068	29	31.2	15	16	18	20	10	10	15	17	11	12	13	18	11	13
Uygur-069	29	30	16	16	18	23	11	11	15	18	13	13	10	19	11	11
Uygur-070	30	35.2	15	16	19	24	11	11	15	15	11	11	12	20	12	12
Uygur-071	30	33.2	15	15	17	23	10	15	16	18	12	13	18	18	11	11
Uygur-072	30.2	33.2	11	16	20	20	10	15	15	18	10	13	11	13	11	12
Uygur-073	30.2	31.2	11	17	20	25	11	12	16	17	12	13	11	18	8	12
Uygur-074	29	30	15	15	19	28	11	11	18	18	13	13	20	21	8	10
Uygur-075	30	33.2	16	17	19	23	10	11	15	15	10	13	18	19	12	13
Uygur-076	29	33.2	14	17	19	19	10	15	14	16	11	12	11	11	8	10
Uygur-077	29	30	11	14	20	23	10	12	15	17	12	12	10	12	10	11
Uygur-078	29	31	16	16	20	28	10	10	15	17	11	12	14	19	8	9
Uygur-079	31.2	33.2	16	17	20	24	10	11	15	17	11	13	11	18	8	8
Uygur-080	28	31	11	15	19	21	11	11	16	18	10	11	10	19	12	12
Uygur-081	29	30	16	16	17	21	11	11	16	16	10	11	11	11	12	12
Uygur-082	29	30	16	16	18	19	10	13	15	18	9	12	12	18	8	11
Uygur-083	29	32.2	15	15	18	25	10	11	15	16	12	13	11	20	9	12
Uygur-084	29	30	14	16	18	24	10	10	16	17	11	11	12	14	10	11
Uygur-085	28	30	16	16	19	23	10	11	14	17	11	12	17	18	8	11
Uygur-086	30	32.2	11	15	20	23	10	10	15	18	11	12	18	20	10	13
Uygur-087	30	32.2	11	11	19	21	9.1	11	15	18	10	11	12	18	8	10
Uygur-088	29	29	15	16	19	23	11	15	17	18	10	13	13	18	9	11
Uygur-089	29	31.2	11	16	19	21	10	10	15	17	10	13	14	19	11	11
Uygur-090	30	30	11	15	24	25	10	11	15	15	12	12	14	14	11	11
Uygur-091	30	31.2	11	15	18	19	11	12	17	17	9	12	14	14	10	10
Uygur-092	30	31	17	17	20	21	10	11	15	15	12	13	13	20	8	12
Uygur-093	31	31.2	14	16	19	25	10	11	16	16	11	12	14	18	9	11
Uygur-094	29	30	16	16	18	18	11	15	15	16	9	11	12	12	8	13
Uygur-095	31.2	33.2	14	16	22	22	11	11	16	17	10	11	11	14	10	11
Uygur-096	29	30	11	15	17	23	11	14	17	18	11	12	10	20	10	13
Uygur-097	28	28	16	17	17	24	9.1	14	16	17	10	12	12	18	8	10
Uygur-098	28	29	16	16	17	20	11	14	15	17	9	13	10	13	11	11

Uygur-099	29	30	16	16	17	18	10	14	15	16	9	12	12	12	8	8
Uygur-100	29	32.2	15	16	18	23	10	11.3	16	18	10	11	11	12	9	11
Uygur-101	30	30	16	17	17	19	10	11	15	16	11	11	20	20	8	11
Uygur-102	30	31	11	15	19	24	11	12	15	16	12	12	12	18	9	11
Uygur-103	28.2	32.2	16	17	18	23	11	12	15	18	11	11	12	12	9	10
Uygur-104	29	30	11	17	20	23	10	11	16	16	11	13	13	21	12	13
Uygur-105	29	31.2	16	17	17	19	10	11	15	15	12	13	12	14	8	10
Uygur-106	30	32.2	16	17	18	25	11	12	15	15	10	11	12	13	8	10
Uygur-107	29	32.2	11	11	24	24	10	10	15	17	11	12	18	18	8	11
Uygur-108	29	32.2	16	19	19	23	10	11	15	18	9	11	12	20	9	10
Uygur-109	29	30	14	15	19	25	10	11	15	17	12	12	14	14	11	11
Uygur-110	29	30	15	16	18	19	10	15	15	16	11	12	11	18	9	11

Continue Supplementary Table S3

Sample ID	D8S1179	D8S1179	FGA	FGA	Penta D	Penta D	Penta E	Penta E	TH01	TH01	TPOX	TPOX	vWA	vWA
Hui-001	14	17	23	24	9	12	17	18	9	10	11	11	16	16
Hui-002	13	14	21	23	9	9	5	18	6	7	8	9	14	18
Hui-003	14	16	22	23	10	12	12	14	8	9	8	8	14	17
Hui-004	13	13	23	25	9	14	15	23	9	9	8	10	17	17
Hui-005	13	14	25	26	9	11	17	21	6	9	8	11	17	19
Hui-006	15	15	22	22	8	11	7	18	6	9	8	9	14	17
Hui-007	11	15	21	23	11	13	15	17	9	9	8	11	17	18
Hui-008	8	13	18	27	9	11	11	16	6	9	8	8	16	17
Hui-009	12	15	23	24	9	12	17	18	7	9	8	9	15	17
Hui-010	11	13	22	24	9	9	14	15	6	9.3	8	11	14	14
Hui-011	11	15	19	23.2	6	12	5	5	9	9	9	11	14	17
Hui-012	13	13	19	21	12	13	15	15	7	9	8	8	17	17
Hui-013	14	15	24	25	9	13	5	11	9	9	8	8	14	16
Hui-014	12	12	18	22	10	12	16	17	6	9.3	8	9	17	18
Hui-015	12	14	21	22	9	9	18	20	9	9	8	8	16	21
Hui-016	11	12	22	22	10	13	12	14	7	9.3	8	11	14	18
Hui-017	14	16	22	23	12	12	15	16	7	9	9	11	14	17
Hui-018	10	14	23	24	10	12	14	22	9	9	8	11	14	16
Hui-019	13	13	20	25	9	9	17	18	9	9	8	11	14	19
Hui-020	12	14	22	24	9	10	19	21	7	10	8	11	14	19
Hui-021	13	14	25	26	9	10	15	19	6	9	11	12	14	19
Hui-022	12	15	22	25	11	12	12	12	8	9	8	8	15	18
Hui-023	11	12	22	23	10	12	11	16	6	7	8	11	18	18
Hui-024	12	15	25	27	8	11	12	14	7	8	8	8	18	18
Hui-025	15	15	23	26	9	9	5	5	9	9.3	8	11	17	19
Hui-026	15	17	18	23	10	12	11	14	7	9	8	9	17	19
Hui-027	13	14	21	25	8	12	10	18	7	9	11	11	14	16
Hui-028	12	15	21	22	8	11	12	13	9	9	9	9	18	19
Hui-029	14	16	18	24	9	13	11	17	6	7	8	12	17	18
Hui-030	11	13	22	23	12	12	12	18	7	9	8	11	16	18
Hui-031	12	13	19	23	8	10	11	13	6	7	8	11	15	16
Hui-032	14	16	24	24	7	9	12	12	7	9.3	8	8	17	17
Hui-033	15	15	18	23	10	12	18	18	9	9	8	11	14	14
Hui-034	13	14	22	23	10	15	11	17	6	8	8	11	17	19

Hui-035	10	14	21	22	9	11	19	24	7	9	8	9	14	18
Hui-036	13	14	19	23	9	9	10	20	9	9	8	8	14	16
Hui-037	13	16	23	24	9	9	12	15	9	9	8	11	16	17
Hui-038	14	16	22	23	9	13	17	21	9	9	8	11	14	16
Hui-039	13	14	22	25	9	9	11	20	9	9	8	11	14	16
Hui-040	11	13	24	26	9	11	13	18	6	7	8	8	17	18
Hui-041	13	14	21	21	9	9	18	19	9	9	8	8	16	18
Hui-042	10	14	18	21	12	13	10	18	9	9.3	8	11	14	14
Hui-043	12	12	23	26	8	12	12	17	9	9	8	8	17	17
Hui-044	10	14	24	25	9	10	17	18.4	7	9.3	8	11	14	20
Hui-045	12	13	19	20	9	12	14	15	6	6	8	8	18	18
Hui-046	13	14	19	22	9	10	5	17	7	9	8	8	16	17
Hui-047	12	15	25	27	9	12	11	18	6	7	8	8	14	17
Hui-048	12	13	25	25	9	9	13	16	9	9	8	11	17	18
Hui-049	13	16	24	24	10	13	11	12	8	9	8	11	14	17
Hui-050	13	14	20	22	9	11	8	11	9	9	11	11	14	19
Hui-051	10	13	22	23	9	11	13	14	6	9	8	11	14	17
Hui-052	10	10	20	24	10	10	17	19	7	7	11	12	16	17
Hui-053	14	14	24	25	9	13	11	17	6	9	8	10	14	18
Hui-054	13	15	24	24	9	9	10	12	7	7	8	9	16	18
Hui-055	14	14	20	23	9	14	11	20	8	9	9	9	16	17
Hui-056	13	13	20	23	10	13	13	20	7	9	8	8	17	17
Hui-057	13	16	18	26	10	11	11	16	9	9	8	9	17	18
Hui-058	13	14	22	24	11	13	16	20	9	9	9	11	15	16
Hui-059	13	18	21	26.2	9	13	14	16	9	9	9	9	14	16
Hui-060	13	14	21	22	11	13	12	15	7	9	8	11	16	20
Hui-061	15	16	19	24	9	10	11	11	7	11	8	11	14	18
Hui-062	12	15	22	24	11	12	14	14	7	9	11	11	16	18
Hui-063	10	13	21	23	9	11	11	11	7	9.3	8	11	18	18
Hui-064	13	14	20	21	10	10	11	14	6	6	8	9	18	19
Hui-065	10	16	22	24	9	11	13	18	9	9	11	12	14	17
Hui-066	14	14	22	23	9	10	16	17	6	9	8	12	14	17
Hui-067	14	15	21	26	14	14	16	17	7	9	8	11	17	18
Hui-068	10	10	24	24	10	13	12	17	7	8	8	8	14	16
Hui-069	13	15	24	25	8	10	12	20	8	9.3	8	11	14	14
Hui-070	12	13	22	23	9	14	8	14	6	7	8	9	17	18
Hui-071	14	14	24	25	9	10	14	18	9	9	11	12	16	17
Hui-072	12	15	23	23	10	13	10	15	9	9	8	11	17	18
Hui-073	10	10	21	21	10	13	14	14	9	9	8	11	14	18
Hui-074	11	14	21	28	12	14	15	16	7	9	9	12	14	16
Hui-075	15	16	21	25	9	13	10	15	7	9	8	9	14	17
Hui-076	12	13	21	23	9	12	5	12	6	7	11	11	17	17
Hui-077	12	15	22	23	11	12	18	20	6	9.3	8	11	14	17
Hui-078	13	15	20	22	9	10	7	21	6	8	8	9	15	19
Hui-079	14	16	19	25	10	11	13	14	7	7	8	9	17	18
Hui-080	13	14	22	26	12	13	12	17	6	7	9	11	16	18
Hui-081	12	13	19	21	12	14	12	12	6	7	9	11	19	19
Hui-082	13	15	18	24	10	13	5	16	9	9.3	8	11	16	17
Hui-083	14	16	21	21	8	12	10	15	8	9	8	8	15	16

Hui-084	13	15	24	24	9	12	17	21	7	9	8	11	16	17
Hui-085	11	14	22	25	10	10	19	21	8	9	8	9	16	17
Hui-086	12	13	22	24	9	11	11	15	9	9	8	9	17	18
Hui-087	10	15	24	24	9	14	15	16	9	9	8	9	17	17
Hui-088	12	13	24	24	11	13	14	16	7	9	8	11	17	18
Hui-089	11	12	22	26	9	11	5	21	7	9	8	11	14	17
Hui-090	13	15	22	22	13	13	11	15	6	6	8	11	17	18
Hui-091	13	14	24	25	11	13	17	20	9	10	11	11	14	16
Hui-092	14	14	23	23.2	7	13	14	21	7	9	8	9	14	16
Hui-093	12	15	23	25	12	13	20	22	9	9	8	8	14	15
Hui-094	15	16	20	21	10	11	17	20	6	9	8	8	18	18
Hui-095	13	14	23	23	11	11	9	17	9	9.3	8	11	18	18
Hui-096	10	17	23	23	9	13	5	15	9	9	8	9	14	20
Hui-097	14	15	23	24	10	12	11	22	6	9	8	9	17	19
Hui-098	13	14	23	25	7	10	11	14	6	12	9	11	14	18
Hui-099	12	16	20	22	12	13	17	20	9	9	8	9	18	19
Hui-100	13	15	23	24	12	12	7	21	9	10	8	11	15	16
Hui-101	11	15	20	21	9	12	18	19	9	9	8	11	16	17
Hui-102	11	15	21	23	12	12	12	16	7	9	8	8	16	16
Hui-103	12	13	22.2	24	9	12	5	19	6	7	11	12	16	18
Hui-104	13	16	21	23	9	10	14	16	6	6	8	11	14	17
Hui-105	13	13	21	25	11	11	14	17	9	9	8	10	18	19
Hui-106	10	13	22	24	9	9	11	20	9	9.3	11	11	14	17
Hui-107	14	17	23	26	9	14	16	19	7	9	11	11	15	17
Hui-108	12	13	22	22	11	11	12	12	9	9	8	11	16	17
Hui-109	13	13	20	23	10	11	12	16	9	9	8	11	17	17
Hui-110	13	14	23	23	14	15	11	12	7	9	8	9	16	18
Hui-111	15	15	23	23	10	13	12	18	9	9.3	9	12	14	16
Hui-112	13	14	19	20	9	14	18	20	7	7	8	8	14	18
Hui-113	13	15	23	24	12	12	13	13	6	7	8	8	16	17
Hui-114	13	15	23	24	8	12	13	16	7	9.3	8	8	14	14
Hui-115	10	13	21	26.2	6	10	11	17	6	9	8	8	18	18
Hui-116	10	13	23	25	11	13	10	11	7	9	8	8	14	17
Hui-117	10	13	22	25	8	9	19.4	24	7	9	8	11	14	18
Hui-118	13	15	21	21	10	13	13	14	9	9.3	9	11	16	17
Hui-119	12	15	22	22	9	12	15	20	9	9.3	8	12	17	17
Hui-120	10	14	21	25	10	12	11	17	9	9	8	11	14	18
Hui-121	15	15	20	23	8	13	13	14	6	7	9	9	16	18
Hui-122	14	14	22	22	8	10	16	18	6	6	8	9	15	17
Hui-123	15	15	21	21	8	11	13	13	9	9	9	11	18	18
Hui-124	12	14	17	23	9	13	11	19	7	9	8	11	14	15
Hui-125	13	15	23	25	10	11	10	17	6	9	8	11	16	17
Hui-126	12	13	22	24	9	9	11	12	9	9	8	11	18	18
Hui-127	15	15	24	24	13	14	12	13	7	9	11	12	16	17
Hui-128	12	13	20	22	9	11	14	16	7	9	8	11	16	17
Hui-129	10	15	21	23	11	13	10	14	8	9	8	8	16	19
Hui-130	13	14	24	26	11	11	11	11	9	9	8	9	18	18
Hui-131	14	16	23	26	11	12	13	16	6	9	11	11	14	16
Hui-132	14	15	24	25	9	12	10	20	7	9	9	11	14	17

Hui-133	13	14	22	24	9	12	12	17	7	7	8	11	16	18
Hui-134	13	14	23	23	10	12	5	12	9	9	8	11	16	18
Hui-135	13	15	23	24	9	11	7	15	9	9	8	9	17	18
Hui-136	13	13	24	24	6	12	10	16	9	9	9	11	17	17
Hui-137	15	15	22	23	9	13	10	14	7	9	8	8	14	18
Hui-138	12	16	23	24	9	13	15	17	9	9.3	9	11	14	18
Hui-139	12	15	22	23	11	11	16	19	7	7	9	11	17	19
Hui-140	14	15	22	22	8	12	5	19	9	9.3	8	9	14	15
Hui-141	10	14	23	24	9	9	16	19	9	9	8	8	16	18
Hui-142	12	17	22	25	10	13	11	22	7	8	9	11	14	18
Hui-143	14	15	23	24	9	14	13	16	9	9	8	9	14	14
Hui-144	11	14	20	25	11	14	12	22	7	10	8	11	16	19
Hui-145	12	13	22	24	9	14	11	19	8	9	8	11	14	18
Hui-146	13	14	21	22	9	9	12	17	9	9	8	8	18	18
Hui-147	14	14	23	23	8	13	18	20	7	9	8	8	14	18
Hui-148	14	15	22	22	12	12	14	19	9	10	9	11	16	17
Hui-149	12	16	23	25	11	11	5	12	7	9	8	8	14	16
Hui-150	12	14	22	24	9	10	16	17	9	9	8	10	17	18
Hui-151	12	15	23	24	8	14	11	19	7	9	8	11	14	14
Hui-152	13	13	24	24	8	13	5	18	9	9	8	9	16	16
Hui-153	12	13	23	24	9	13	15	15	9	9	8	10	14	16
Hui-154	10	11	21	26	9	11	5	11	6	9	11	11	14	17
Hui-155	13	13	19	22	9	9	14	19	9	9	8	11	15	17
Hui-156	14	15	21	24	9	10	13	16	7	9	8	11	18	18
Hui-157	13	15	23	26	9	12	11	14	9	9	8	8	14	19
Hui-158	10	11	20	25	9	9	10	16	8	9	8	8	18	19
Hui-159	14	16	20	23	11	13	17	18	7	9	9	11	16	16
Hui-160	12	12	23	26	9	10	13	18	7	9	8	8	16	19
Hui-161	14	16	22	26	11	13	16	16	7	9	11	11	16	17
Hui-162	13	16	20	25	9	11	12	20	9	9.3	8	11	16	18
Hui-163	13	16	18	22.2	9	10	11	12	9	9.3	8	12	14	17
Hui-164	10	13	25	27	8	9	5	21	6	9.3	8	8	13	14
Hui-165	12	16	21	21	9	12	14	15	9	9	8	8	16	19
Hui-166	10	13	23	24	9	9	16	18	9	9.3	9	11	16	17
Hui-167	14	15	22	24	11	12	7	17	9	9	8	11	14	17
Hui-168	14	16	22	26	9	11	12	15	9	9	8	9	18	20
Hui-169	10	11	23	24	9	14	12	13	7	8	8	12	14	17
Hui-170	11	12	24	26	9	12	14	20	7	7	8	9	14	17
Hui-171	12	13	24	24	9	12	14	16	8	9	8	8	16	19
Hui-172	13	13	24	24	12	12	14	24	9	9	9	11	15	16
Hui-173	12	14	20	24	9	12	14	21	9	9	11	11	17	17
Hui-174	13	13	20	22	9	13	13	16	8	9.3	8	8	14	16
Hui-175	11	13	20	21	9	9	5	12	6	7	8	8	16	17
Hui-176	13	16	21	25	10	13	15	19	8	9	8	11	16	18
Hui-177	11	13	23	26	9	11	13	14	6	9	8	11	14	18
Hui-178	14	14	22	23	9	12	5	11	6	7	9	11	14	17
Hui-179	14	15	22	25	9	10	15	19	7	8	8	12	14	17
Hui-180	13	15	21	24	8	13	14	15	9	9	10	11	14	17
Hui-181	11	16	22	24	9	14	7	10	9	9	8	9	14	18

Hui-182	13	14	22	24	12	12	11	15	8	9	8	10	14	19
Hui-183	14	15	21	25	9	11	16	17	7	9	8	11	14	16
Tibetan-001	14	14	22	24	11	11	10	13	7	7	8	11	14	19
Tibetan-002	13	14	22	24	9	12	12	13	7	9	8	12	16	17
Tibetan-003	13	13	22	23	9	11	11	18	8	9.3	8	11	17	19
Tibetan-004	10	10	18	23	10	12	12	17	9	9	8	11	16	17
Tibetan-005	13	14	23	23	9	9	12	21	6	7	8	8	16	17
Tibetan-006	13	14	24	25	9	12	14	18	7	8	8	8	14	16
Tibetan-007	13	14	22	24	9	9	16	16	6	7	9	11	16	19
Tibetan-008	12	14	24	25	12	14	11	15	7	9	8	8	18	18
Tibetan-009	12	14	18	23	11	13	10	21	7	9	8	11	14	17
Tibetan-010	14	15	22	23	10	11	13	17	7	9	8	9	16	20
Tibetan-011	13	14	23	25	9	11	13	14	9	9	8	9	16	18
Tibetan-012	10	15	22	23	11	13	10	15	9	9	11	11	18	19
Tibetan-013	13	14	21	22	7	9	14	21	9	9	8	11	14	15
Tibetan-014	10	14	21	22	9	12	13	19	9	9	8	8	16	17
Tibetan-015	14	14	18	23	9	9	22	24	6	6	9	11	14	17
Tibetan-016	15	16	19	21	10	13	10	16	7	9	8	11	17	19
Tibetan-017	15	16	21	22	10	11	10	14	7	8	8	8	16	17
Tibetan-018	13	15	22	25	9	11	11	12	6	9.3	8	8	16	17
Tibetan-019	13	14	23	25	9	13	13	20	6	7	8	11	16	20
Tibetan-020	10	13	20	23	9	10	12	14	7	9	11	11	18	18
Tibetan-021	10	12	23	24	9	11	11	11	9	9	8	9	17	18
Tibetan-022	10	14	23	23	10	13	10	19	9	9	8	8	16	18
Tibetan-023	12	13	18	22.2	9	11	15	16	9	9	8	11	16	18
Tibetan-024	13	13	21	23	12	13	15	18	9	9	8	11	16	19
Tibetan-025	12	14	22.2	24	11	13	16	22	6	9	8	11	14	16
Tibetan-026	10	13	18	22	9	10	13	18	7	7	8	8	18	19
Tibetan-027	12	13	22	23	9	10	14	19	6	7	8	9	17	18
Tibetan-028	11	15	22	24	10	13	12	15	6	7	8	8	16	17
Tibetan-029	14	16	22	26	9	10	12	15	9	9	8	11	16	19
Tibetan-030	12	15	21	26	9	9	18	21	9	9	9	11	18	19
Tibetan-031	12	16	18	18	10	10	12	13	7	9	11	11	15	17
Tibetan-032	10	14	18	26	11	12	15	16	6	7	8	11	17	19
Tibetan-033	14	15	22	23.2	9	9	16	21	9	9	8	8	17	18
Tibetan-034	11	16	18	22	9	13	11	13	7	9.3	8	8	18	18
Tibetan-035	13	15	22	24	8	10	12	15	7	9	8	11	17	17
Tibetan-036	10	14	21	25	13	13	14	18	9	9	9	11	18	19
Tibetan-037	12	13	22	26	10	11	10	19	7	9	8	8	14	17
Tibetan-038	13	14	25	26.2	9	11	14	18	7	7	8	11	18	19
Tibetan-039	10	14	24	25	9	10	20	24	9	9	8	8	14	16
Tibetan-040	12	14	18	18	10	12	14	17	9	9	8	9	17	19
Tibetan-041	12	15	22	23	6	13	11	14	9	9	8	11	14	14
Tibetan-042	13	14	18	24	9	11	11	12	7	9	8	11	14	14
Tibetan-043	14	15	22	26	10	11	11	16	9	9.3	8	8	14	19
Tibetan-044	12	14	18	22	9	10	15	21	7	7	8	11	17	18
Tibetan-045	15	15	23	26	9	12	16	16	9	9	11	11	18	19
Tibetan-046	13	16	22	23.2	9	10	9	11	8	9	8	11	16	17
Tibetan-047	10	12	18	23	11	12	11	13	8	9	8	9	14	18

Tibetan-048	12	13	23	25	9	12	17	19	6	9	8	8	17	20
Tibetan-049	12	13	22	23	9	12	5	11	9	9	11	11	16	16
Tibetan-050	13	15	23	24	9	10	15	19	9	9	8	11	14	14
Tibetan-051	13	14	18	25	9	13	12	15	7	9	9	11	16	18
Tibetan-052	10	14	21	26	11	12	12	15	7	9	8	8	14	18
Tibetan-053	12	15	21	22	13	14	12	14	9	9	8	9	14	18
Tibetan-054	12	14	24	24	11	13	11	16	7	9	8	11	16	17
Tibetan-055	15	15	19	24	9	9	9	11	7	7	11	11	17	18
Tibetan-056	10	13	18	23	9	13	11	19	9	9.3	8	8	16	17
Tibetan-057	11	15	18	25	11	13	17	18	6	9	8	8	14	17
Tibetan-058	12	13	18	24	9	13	15	22	7	9	11	11	14	18
Tibetan-059	14	15	23.2	25	9	11	16	19	6	9	8	8	14	19
Tibetan-060	10	13	25	26	11	11	15	19	7	9	8	11	14	19
Tibetan-061	10	13	23	23	7	9	10	16	6	7	9	11	17	18
Tibetan-062	13	15	23	25	11	13	14	15	9	9	8	8	18	20
Tibetan-063	14	14	18	24	10	11	12	17	6	9	8	8	14	14
Tibetan-064	13	14	23	25	10	12	11	18	8	9	8	11	14	16
Tibetan-065	14	15	22	26	11	13	18	18	7	7	8	8	17	19
Tibetan-066	14	14	22	24	6	13	16	19	9	9	8	8	17	18
Tibetan-067	10	10	22	23	9	9	13	20	9	9	8	9	17	18
Tibetan-068	13	15	22	23	11	13	15	16	8	9	11	11	14	15
Tibetan-069	12	13	21	23	8	11	17	19	7	9	8	11	16	17
Tibetan-070	12	12	18	22	6	10	15	17	7	9	8	12	17	17
Tibetan-071	14	14	22	22	9	13	10	12	9	9	8	9	14	16
Tibetan-072	13	16	19	23	9	9	5	11	9	9	11	11	17	17
Tibetan-073	12	15	20	23	9	9	12	14	9	9	8	8	14	17
Tibetan-074	12	13	23	25	9	11	15	19	7	9	8	8	14	19
Tibetan-075	10	17	24	24	11	13	5	19	6	9	9	11	17	19
Tibetan-076	13	13	22	24	11	12	12	15	6	7	8	8	19	19
Tibetan-077	14	14	23	26	9	13	13	15	9	9	8	11	15	16
Tibetan-078	14	15	18	22	8	12	14	16	9	9	8	8	16	17
Tibetan-079	12	13	23	24	9	9	14	18	9	9.3	9	11	18	18
Tibetan-080	12	15	22	24	10	15	12	12	6	7	8	11	18	18
Tibetan-081	14	15	18	22	11	11	13	25	7	9	8	8	14	16
Tibetan-082	13	14	23	23.2	10	10	15	28	7	9	8	8	14	20
Tibetan-083	12	15	22	24	10	11	15	19	9	9	8	8	14	18
Tibetan-084	10	15	22	22	9	9	15	19	7	9	8	11	16	17
Tibetan-085	10	15	22	23.2	9	10	15	15	7	9	8	9	14	18
Tibetan-086	13	15	23	23	11	14	11	14	8	9	8	8	18	19
Tibetan-087	12	15	21	23	9	10	12	21	7	7	8	8	16	18
Tibetan-088	13	14	18	24	9	11	15	24	6	7	8	11	14	16
Tibetan-089	11	14	21	24	11	13	12	17	7	9	8	9	16	18
Tibetan-090	13	14	21	22	11	11	16	17	7	7	8	12	18	20
Tibetan-091	14	15	18	23	9	10	9	23	7	9	8	8	16	18
Tibetan-092	14	15	19	20	11	11	16	18	9	9	8	8	16	18
Tibetan-093	11	14	20	22	9	11	10	15	7	9	8	8	17	18
Tibetan-094	13	14	20	22	9	13	16	17	9	9	11	11	14	16
Tibetan-095	12	14	19	26	11	13	16	19	9	9.3	8	9	16	16
Tibetan-096	12	13	22	25	9	9	15	19	9	9.3	9	9	15	18

Tibetan-097	11	12	20	22	9	10	14	15	9	9	9	9	16	19
Tibetan-098	14	15	20	24	10	13	12	20	9	9	8	8	14	16
Tibetan-099	13	13	18	21.2	7	9	14	14	7	9	8	11	14	16
Tibetan-100	12	13	18	24	9	11	12	17	9	9.3	11	11	16	18
Tibetan-101	13	13	23	25	11	13	18	23	6	8	8	11	18	19
Tibetan-102	12	13	24	24	11	12	10	18	7	7	8	8	16	18
Tibetan-103	13	16	24	24	11	12	12	21	9	9	8	11	17	18
Tibetan-104	13	15	23	23	9	9	11	18	9	9	8	9	17	17
Tibetan-105	13	15	22	26	9	9	5	22	9	9	9	11	16	17
Tibetan-106	12	13	18	23	9	13	17	18	6	9	8	11	16	17
Tibetan-107	13	14	18	20	9	12	15	16	9	9	8	8	17	18
Tibetan-108	10	15	22	23	9	12	17	22	6	7	8	11	17	17
Tibetan-109	14	15	22	24	12	13	12	19	9	9.3	8	8	16	17
Tibetan-110	14	15	21	24	9	11	15	20	7	9.3	8	9	17	17
Tibetan-111	11	15	26	26	9	9	14	15	7	9	8	11	16	18
Tibetan-112	13	16	19	23	9	10	20	21	7	7	8	8	16	16
Tibetan-113	10	12	23	23.2	9	11	10	15	9	9	8	8	16	17
Tibetan-114	14	16	18	25	9	9	11	22	9	9	8	9	17	19
Tibetan-115	11	14	18	22	9	11	15	19	7	9	8	9	14	18
Tibetan-116	13	15	21	24	9	9	15	16	9	9.3	8	9	17	18
Tibetan-117	14	15	18	23	9	10	13	22	7	9	8	11	17	19
Tibetan-118	14	16	21	25	9	11	9	12	9	9	8	11	16	16
Tibetan-119	14	15	19	20	11	13	5	11	7	9	8	8	17	17
Tibetan-120	11	15	18	24	9	10	12	19	9	9	9	11	14	17
Tibetan-121	11	15	23	24	10	12	14	20	7	9	8	8	14	15
Tibetan-122	13	15	20	22	9	12	12	16	9	9	8	11	17	19
Tibetan-123	12	15	22	26	9	9	19	20	7	9	8	8	14	14
Tibetan-124	12	14	22	25	10	12	5	16	9	9.3	9	12	18	18
Tibetan-125	12	14	22	24	9	10	14	16	7	8	8	11	14	18
Tibetan-126	10	12	21	23	8	11	14	25	9	9	8	11	16	17
Tibetan-127	10	16	21	23	9	13	11	14	9	9	8	9	17	19
Tibetan-128	10	13	24	24	9	9	19	20	7	9	8	8	14	17
Tibetan-129	13	15	19	24	10	12	15	25	6	7	8	8	16	17
Tibetan-130	14	15	18	26	9	11	22	23	9	9	8	11	14	18
Tibetan-131	10	14	23	24	9	12	12	15	7	9	8	11	16	19
Tibetan-132	10	12	22	22	9	10	19	22	9	9	8	9	16	17
Tibetan-133	10	12	19	25	9	12	11	19	6	7	8	11	16	17
Tibetan-134	10	16	18	23	9	11	11	22	7	9	8	8	17	21
Tibetan-135	13	13	20	24	10	12	10	12	7	7	8	11	18	19
Tibetan-136	13	14	22	23	9	11	21	28	6	9.3	8	11	16	19
Tibetan-137	12	13	22	23	8	10	9	12	7	9	8	8	14	14
Tibetan-138	12	12	21	22.2	9	10	5	16	7	9.3	8	8	16	18
Tibetan-139	10	15	23	23.2	10	11	10	22	7	9	8	8	16	17
Tibetan-140	13	16	18	23	9	10	12	20	7	9	8	8	18	19
Tibetan-141	13	15	22	25	9	10	17	19	7	9	8	11	14	19
Tibetan-142	14	14	23	24	9	11	10	17	9	9	8	11	14	18
Tibetan-143	14	14	18	23	9	10	12	15	6	7	11	11	18	18
Tibetan-144	12	16	25.2	25.2	12	13	15	20	9	9	8	8	14	19
Tibetan-145	13	13	22	23	9	10	12	18	9	9.3	8	11	14	18

Tibetan-146	13	14	22	25	10	10	15	22	7	9	8	8	16	18
Tibetan-147	15	17	23	23	9	11	12	19	9	9	8	8	14	17
Tibetan-148	10	12	18	24	10	12	15	15	7	9	8	8	14	18
Tibetan-149	10	13	23	24	9	10	13	16	7	9	8	11	14	16
Tibetan-150	13	13	20	23	10	13	15	18	9	9	8	8	17	18
Tibetan-151	12	14	22	26	9	11	8	16	7	9	8	8	16	18
Tibetan-152	13	14	21	23	7	14	11	17	9	9.3	8	8	19	19
Tibetan-153	14	16	23	25	9	10	10	23	7	9	9	11	16	17
Tibetan-154	11	14	23	24	9	12	12	20	7	9	8	11	14	17
Tibetan-155	13	13	18	22	13	14	10	19	9.3	9.3	8	11	16	19
Tibetan-156	12	14	21	24	9	9	11	14	9	9	9	11	16	18
Tibetan-157	14	15	18	24	7	12	18	18	6	9	8	8	16	19
Tibetan-158	13	15	24	25	9	9	11	14	7	9.3	8	9	17	17
Tibetan-159	13	15	24	25.2	11	12	9	18	9	9	8	8	18	20
Tibetan-160	12	12	22	23	11	11	11	17	9	9	9	9	16	18
Tibetan-161	12	14	19	23	9	11	12	12	7	9	11	11	16	17
Tibetan-162	12	15	24	25	9	10	12	15	9	9	11	12	14	17
Tibetan-163	15	16	22	26	11	12	10	25	7	9	8	8	18	18
Tibetan-164	10	12	22	22	11	12	12	17	9	9.3	8	11	18	18
Tibetan-165	12	15	22	24	13	14	13	16	7	9	8	8	14	18
Tibetan-166	10	16	22	22	10	11	11	23	7	9	8	11	17	17
Tibetan-167	10	11	24	25	9	10	12	24	7	9	8	8	14	18
Tibetan-168	13	15	23	24	12	12	12	18	9	9	8	9	16	18
Tibetan-169	12	14	18	23	11	12	12	18	6	9	8	9	16	17
Tibetan-170	12	13	24	26	9	12	12	18	6	7	8	8	16	17
Tibetan-171	14	15	23	23	9	12	11	16	7	9	8	8	14	18
Tibetan-172	14	15	22	22	9	11	10	16	7	9	8	8	16	19
Tibetan-173	13	14	19	23	9	11	20	22	6	7	8	9	14	18
Tibetan-174	15	15	21	23	9	11	18	20	9	9	8	8	17	18
Tibetan-175	12	17	22	25	9	11	19	22	6	9.3	8	11	14	17
Tibetan-176	15	16	22	22.2	8	11	14	20	8	9	8	11	18	19
Tibetan-177	14	16	18	27.2	12	13	13	15	9	9	8	11	16	18
Tibetan-178	14	16	22	25	9	11	20	21	7	9	8	11	17	18
Tibetan-179	15	17	21	25	11	14	11	14	7	9.3	9	11	14	16
Tibetan-180	14	15	20	22	9	11	10	17	9	9	8	11	17	18
Tibetan-181	12	16	20	25.2	9	11	11	15	6	9	8	11	17	18
Tibetan-182	14	14	23	23	9	12	12	15	7	9	8	8	16	17
Tibetan-183	12	13	18	25	9	12	12	18	6	9	8	11	16	16
Tibetan-184	12	13	23.2	26	7	13	11	17	9	9	8	8	16	16
Tibetan-185	12	13	24	25	10	12	14	16	7	9	9	11	14	16
Tibetan-186	13	13	20	23	10	11	14	18	7	9	11	11	16	18
Tibetan-187	11	12	23	25	7	11	5	11	7	9	8	8	14	14
Tibetan-188	14	15	23	23	9	10	10	12	9	9	8	10	14	16
Tibetan-189	10	13	18	22	11	13	15	16	7	9	11	11	16	17
Tibetan-190	10	10	18	25	9	9	14	20	7	7	9	11	14	17
Tibetan-191	13	14	19	26	11	15	11	18	7	9	8	11	17	17
Tibetan-192	13	17	23	23	11	13	12	18	9	9	8	12	14	16
Tibetan-193	12	17	23	25	11	14	16	19	7	9	8	12	16	19
Tibetan-194	10	11	24.2	25	12	13	5	20	7	7	8	11	17	18

Tibetan-195	14	15	22	24	11	13	15	19	9	9.3	8	9	16	17
Tibetan-196	10	15	19	25.2	9	9	13	17	7	7	10	12	17	19
Tibetan-197	12	15	20	21	11	11	5	20	7	9	11	11	16	16
Tibetan-198	12	14	24	24	10	14	13	19	7	9	8	11	16	19
Tibetan-199	12	13	22	23	9	10	17	19	9	9.3	9	11	17	17
Tibetan-200	14	14	23	24	12	12	17	20	7	10	8	9	16	16
Uygur-001	14	16	20	25	9	11	5	8	6	7	11	12	17	17
Uygur-002	14	15	23	25	12	12	5	7	6	9.3	11	11	17	19
Uygur-003	12	16	22	22	13	13	7	11	6	9	8	11	14	16
Uygur-004	12	13	23	25	9	12	12	13	7	9	9	11	15	17
Uygur-005	12	13	22.2	23	9	13	5	12	6	7	8	11	16	19
Uygur-006	13	15	21	22	9	12	12	13	9	9	9	11	15	17
Uygur-007	12	15	23	25	9	14	12	14	6	8	8	11	17	19
Uygur-008	11	14	23	24	9	9	5	12	6	7	8	11	17	19
Uygur-009	12	13	22	24	9	11	11	12	6	6	8	8	16	17
Uygur-010	13	13	22	28	11	12	18	18	7	9	8	8	14	14
Uygur-011	11	15	23	24	11	13	7	7	7	8	8	8	14	19
Uygur-012	13	15	22	23	7	10	11	12	9	9.3	11	11	17	18
Uygur-013	13	14	21	22	9	10	7	24	6	7	8	12	16	17
Uygur-014	10	14	21	23	9	11	11	22	9.3	9.3	11	11	17	17
Uygur-015	11	13	23	25	9	14	5	14	9	9.3	8	11	16	17
Uygur-016	11	12	24	26	9	11	10	16	9	9.3	8	11	16	19
Uygur-017	12	13	19	22	9	9	11	15	6	8	8	11	13	16
Uygur-018	10	14	22	24	10	10	13	19	9.3	9.3	8	12	14	19
Uygur-019	13	13	22	22	9	15	10	17	9	10.3	8	8	18	18
Uygur-020	13	15	22	24	9	14	7	13	8	9	11	11	17	17
Uygur-021	13	14	22.2	23	9	11	7	17	6	9.3	8	8	17	20
Uygur-022	13	15	19	23.2	9	13	14	24	6	9	11	13	16	19
Uygur-023	13	15	23	25	11	12	11	11	6	9	11	12	16	17
Uygur-024	10	17	21	25	10	11	11	19	7	9	9	11	16	19
Uygur-025	10	13	22	28	9	10	10	11	6	9	8	9	18	19
Uygur-026	10	11	23	24	9	15	14	17	6	7	8	8	15	17
Uygur-027	13	13	21	22	7	9	12	14	9	9.3	8	8	14	19
Uygur-028	11	15	19	28	11	13	12	18	6	6	8	11	17	17
Uygur-029	14	14	19	25	10	11	7	19	8	9	8	11	17	19
Uygur-030	10	13	19	23	7	10	11	19	9	9.3	8	12	15	19
Uygur-031	11	14	22	24	9	15	5	18	9	9	8	8	16	17
Uygur-032	12	13	23	26	11	13	18	22	9	9	8	11	14	19
Uygur-033	14	14	20	22	9	11	5	10	9	9	9	9	15	18
Uygur-034	13	13	22	22	9	13	5	10	9	9.3	11	12	15	17
Uygur-035	14	15	22	24	12	13	15	15	7	9	8	12	14	19
Uygur-036	10	12	21	28	10	14	11	22	9	9.3	11	12	17	19
Uygur-037	10	12	23	26	13	14	15	16	9	9	9	11	17	20
Uygur-038	12	13	23	26	6	9	14	22	6	9	9	11	17	17
Uygur-039	13	13	19	24	11	14	11	15	9	9.3	8	11	16	18
Uygur-040	12	13	24	26	9	10	14	17	6	9.3	8	11	17	18
Uygur-041	11	13	21	23	9	11	7	11	6	7	8	8	17	19
Uygur-042	11	12	19	23	11	13	12	12	6	9.3	8	11	17	18
Uygur-043	12	13	22	23	9	9	7	13	8	9	11	11	15	17

Uygur-044	13	14	22	25	9	11	5	18	6	9	8	11	17	17
Uygur-045	11	15	23	28	10	11	7	7	6	8	8	8	16	19
Uygur-046	14	14	23	24	9	12	5	5	7	8	8	9	14	18
Uygur-047	12	14	23	23	11	15	11	18	9.3	9.3	11	11	17	19
Uygur-048	12	15	20	25	7	10	5	15	7	9	8	8	14	17
Uygur-049	13	13	23	25	9	13	7	14	6	9	8	11	15	17
Uygur-050	13	15	20	25	10	11	13	15	9	9	8	8	16	16
Uygur-051	12	13	20	24	9	9	14	14	6	9.3	8	11	14	17
Uygur-052	10	11	20	26	9	11	14	15	6	9	8	11	17	17
Uygur-053	12	13	20	26	10	11	15	15	6	9	8	8	17	18
Uygur-054	13	14	20	25	11	12	12	15	9	9	8	11	17	19
Uygur-055	10	13	22	23	7	9	12	19	6	9.3	11	12	15	19
Uygur-056	11	15	20	25	10	11	10	13	9	9	8	8	15	16
Uygur-057	11	13	23	23	9	13	12	17	7	7	8	11	15	16
Uygur-058	10	14	23	24	11	12	11	23	9	9.3	9	11	14	17
Uygur-059	14	15	26	28	9	12	5	5	6	7	10	11	14	18
Uygur-060	12	13	26	26	9	11	11	19	6	9.3	8	11	17	19
Uygur-061	13	14	23	25	9	13	14	16	9	9.3	8	11	16	18
Uygur-062	14	15	24	24	11	12	12	15	6	7	8	8	14	19
Uygur-063	13	14	21	23	9	12	16	18	9	9	8	12	17	18
Uygur-064	13	14	23	23	9	9	13	16	7	9.3	8	11	16	18
Uygur-065	11	12	23	26	9	11	12	15	9	9.3	8	9	19	19
Uygur-066	9	16	20	24	9	11	7	16	6	9	8	11	16	18
Uygur-067	10	13	23	28	9	9	11	15	9	9.3	8	11	17	18
Uygur-068	14	14	21	23	9	11	5	15	9	9.3	8	9	14	14
Uygur-069	12	13	22	25	9	14	11	11	7	9.3	8	8	17	19
Uygur-070	14	15	25	25	9	14	7	11	9	9	8	8	19	19
Uygur-071	10	13	20	22	9	15	11	11	6	9	8	12	16	19
Uygur-072	13	15	23	23	9	11	11	17	9	9	8	11	15	17
Uygur-073	10	14	25	25	10	11	11	14	7	9.3	8	9	16	18
Uygur-074	14	14	24	24	9	11	10	14	9	9	11	11	15	15
Uygur-075	13	15	23	24	10	10	15	15	6	9	8	11	14	19
Uygur-076	10	11	23	25	9	13	11	15	9.3	9.3	11	11	17	17
Uygur-077	11	12	23	25	9	13	10	13	9	9.3	8	12	17	17
Uygur-078	14	15	22	27	9	11	11	22	6	9	8	11	17	19
Uygur-079	13	14	21	22	9	13	13	19	9	9	9	11	17	17
Uygur-080	13	15	23	23	11	14	12	15	9	9	11	12	14	18
Uygur-081	11	15	19	22	9	11	16	18	7	9.3	8	11	16	19
Uygur-082	13	14	20	23	9	11	12	13	6	9	9	11	16	16
Uygur-083	12	13	23	23	10	10	14	19	6	9.3	8	8	14	17
Uygur-084	11	11	22	23	12	13	10	15	7	9	8	11	17	18
Uygur-085	13	13	22.2	25	9	13	11	15	6	9.3	9	11	14	16
Uygur-086	12	14	24.2	26	10	11	14	17	6	6	8	11	17	17
Uygur-087	13	15	21	22	9	9	10	15	6	9	8	9	19	19
Uygur-088	13	14	23	29	10	13	11	11	7	9	8	12	15	18
Uygur-089	14	14	20	23	13	15	5	12	7	10	8	9	16	17
Uygur-090	11	14	20	24	9	10	10	12	7	9	8	12	14	17
Uygur-091	11	14	24	26	9	12	11	14	9	9	8	8	14	16
Uygur-092	12	12	22	24	9	9	11	15	7	8	8	8	16	17

Uygur-093	11	14	24	25	9	10	7	14	9	9	8	8	14	17
Uygur-094	10	12	25	28	12	15	15	18	9	9.3	8	11	16	17
Uygur-095	12	13	25	27	9	13	7	15	7	8	10	12	17	18
Uygur-096	12	13	19	23	11	11	12	15	6	9	8	11	17	17
Uygur-097	13	13	23	28	9	14	11	11	7	9	8	11	17	18
Uygur-098	12	15	19	22	9	9	5	15	9	9	8	11	15	17
Uygur-099	10	13	23	25	9	11	11	15	6	9	9	11	14	16
Uygur-100	13	15	19	25	9	12	18	20	6	9	8	11	14	18
Uygur-101	10	13	21	23	9	14	12	14	6	9	8	11	17	19
Uygur-102	11	15	21	24	10	12	12	22	9	9.3	8	9	15	17
Uygur-103	11	13	24	28	10	12	11	16	9	9	11	12	15	17
Uygur-104	14	16	21	24	10	11	5	8	7	9	8	11	17	17
Uygur-105	13	13	21.2	24	9	13	15	21	8	8	8	9	18	18
Uygur-106	11	15	23	25	13	13	10	12	6	9	8	8	17	18
Uygur-107	13	14	25	28	9	9	10	14	6	9.3	9	12	17	18
Uygur-108	13	13	19	21	9	9	14	21	7	9	11	11	15	15
Uygur-109	12	13	24	25	11	14	10	12	7	9	11	12	17	17
Uygur-110	13	16	23	25	8	10	11	11	7	8	8	8	15	16

Supplementary Table S4. p-values of Linkage Disequilibrium test between all pairs of 23 STR loci in Ningxia Wuzhong Hui population (after Bonferroni correction p > 0.0002).

Locus	L1	L2	L3	L4	L5	L6	L7	L8	L9	L10	L11	L12	L13	L14	L15	L16	L17	L18	L19	L20	L21	L22	L23
[L1]CSF1PO																							
[L2]D10S1248	0.8993																						
[L3]D12S391	0.9997	0.3128																					
[L4]D13S317	0.7534	0.6715	0.9197																				
[L5]D16S539	0.4621	0.5613	0.5651	0.3960																			
[L6]D18S51	0.9631	0.9696	0.9981	0.6959	0.7121																		
[L7]D19S433	0.9898	0.3738	0.9558	0.2808	0.6383	0.9933																	
[L8]D1S1656	0.9920	0.5893	0.7232	0.8944	0.9304	0.9955	0.5757																
[L9]D21S11	0.9962	0.9980	0.9999	0.8525	0.9455	0.9988	0.9129	0.9949															
[L10]D22S1045	0.9622	0.9633	0.7639	0.6981	0.8919	0.6598	0.9965	0.9261	0.9998														
[L11]D2S1338	0.9476	0.8485	0.9573	0.3966	0.6006	0.8346	0.6340	0.5688	0.9420	0.8410													
[L12]D2S441	0.9980	0.4930	0.9376	0.8702	0.6670	0.7637	0.7237	0.9799	0.9365	0.9968	0.8010												
[L13]D3S1358	0.8262	0.9874	0.3670	0.1221	0.5335	0.9682	0.3136	0.6335	0.7665	0.3853	0.6499	0.7932											
[L14]D5S818	0.9897	0.4854	0.8203	0.5431	0.6708	0.9976	0.7375	0.5093	0.9957	0.8854	0.4621	0.4987	0.5939										
[L15]D6S1043	0.9924	0.9507	0.8172	0.8433	0.4015	0.9995	0.9995	1.0000	1.0000	0.9131	0.9777	0.9529	0.8961	0.8821									
[L16]D7S820	0.9980	0.8689	0.9964	0.6172	0.9557	0.8486	0.9991	0.9999	0.9902	0.9519	0.6464	0.9979	0.7815	0.6755	0.9987								
[L17]D8S1179	0.9908	0.9922	0.8183	0.4775	0.5689	0.9493	0.4165	0.9799	0.9728	0.8850	0.8619	0.7694	0.9304	0.8913	0.9772	0.9031							
[L18]FGA	0.9983	0.9457	0.9534	0.8115	0.9078	0.9834	0.9907	0.9966	1.0000	0.9994	0.9872	0.8061	0.6704	0.9050	0.9996	0.9992	0.8718						
[L19]Penta D	0.9830	0.9232	0.8324	0.6360	0.6311	0.7939	0.6435	0.3966	0.9691	0.9798	0.5961	0.9519	0.0594	0.8977	0.9944	0.9309	0.9154	0.8467					
[L20]Penta E	1.0000	0.8966	0.9869	0.4571	0.8470	0.9979	0.9319	0.8669	0.9999	0.9798	0.9626	0.9872	0.9118	0.9712	1.0000	0.9994	0.9962	1.0000	0.9824				
[L21]TH01	0.9979	0.9636	0.5994	0.8900	0.5277	0.9888	0.8098	0.9658	0.9557	0.9576	0.9362	0.9977	0.2032	0.3103	0.9860	0.7949	0.9617	0.9546	0.5967	0.9967			
[L22]TPOX	0.4060	0.3318	0.6185	0.7379	0.8124	0.9581	0.2820	0.9201	0.9322	0.2467	0.7455	0.5969	0.7267	0.7180	0.9868	0.9434	0.4583	0.4165	0.5137	0.9343	0.2040		
[L23]vWA	0.9983	0.9356	0.9930	0.5955	0.6560	0.9935	0.7949	0.5452	0.9006	0.7969	0.7435	0.6864	0.1435	0.7512	0.9976	0.3679	0.9231	0.9794	0.7252	0.8452	0.6053	0.8845	

Supplementary Table S5. p-values of Linkage Disequilibrium test between all pairs of 23 STR loci in Sichuan Chengdu Tibetan population (after Bonferroni correction p > 0.0002).

Locus	L1	L2	L3	L4	L5	L6	L7	L8	L9	L10	L11	L12	L13	L14	L15	L16	L17	L18	L19	L20	L21	L22	L23
[L1]CSF1PO																							
[L2]D10S1248	0.7618																						
[L3]D12S391	0.9875	0.9288																					
[L4]D13S317	0.9921	0.4327	0.6707																				
[L5]D16S539	0.8124	0.8687	0.9281	0.8382																			
[L6]D18S51	0.9955	0.8532	0.7270	0.5681	0.5286																		
[L7]D19S433	0.9464	0.6338	0.8737	0.0184	0.9787	0.6637																	
[L8]D1S1656	0.9833	0.6749	0.9589	0.4584	0.8444	0.9894	0.9758																
[L9]D21S11	0.9957	0.9569	0.9876	0.3839	0.8547	0.9955	0.5230	0.9954															
[L10]D22S1045	0.8395	0.8302	0.2690	0.8236	0.3798	0.5623	0.5344	0.8021	0.5795														
[L11]D2S1338	0.9991	0.6172	0.8336	0.1237	0.6564	0.8352	0.8689	0.9870	0.6009	0.7086													
[L12]D2S441	0.8734	0.4023	0.9665	0.0810	0.6462	0.2332	0.7310	0.8356	0.3510	0.0684	0.5237												
[L13]D3S1358	0.9604	0.7416	0.7987	0.4517	0.7360	0.9505	0.9998	0.9841	0.9380	0.6216	0.9782	0.7729											
[L14]D5S818	0.9809	0.5862	0.5746	0.7562	0.9487	0.7010	0.9463	0.8387	0.0342	0.2508	0.3339	0.7387	0.8810										
[L15]D6S1043	0.9640	0.8268	0.8159	0.9937	0.6127	0.9624	0.8695	0.9776	0.9831	0.5563	0.9955	0.8856	0.9994	0.9918									
[L16]D7S820	0.8352	0.8491	0.5297	0.7647	0.5349	0.5083	0.9483	0.8932	0.7821	0.7592	0.4307	0.7176	0.7011	0.7884	0.9286								
[L17]D8S1179	0.8036	0.0092	0.9891	0.0980	0.3204	0.8236	0.4398	0.2285	0.2844	0.2536	0.1432	0.3210	0.4497	0.1676	0.5865	0.9530							
[L18]FGA	0.9818	0.7284	0.9998	0.8589	0.2368	0.6102	0.2041	0.9981	0.9843	0.9137	0.9968	0.8492	0.9796	0.9943	0.9988	0.9066	0.6154						
[L19]Penta D	0.9676	0.9478	0.6136	0.5786	0.9474	0.5223	0.3048	0.9205	0.9283	0.4180	0.5878	0.2867	0.7896	0.5120	0.8787	0.8047	0.8593	0.9992					
[L20]Penta E	0.9993	0.3753	0.9584	0.5496	0.9834	0.9992	0.8020	0.9805	0.9992	0.1437	0.8295	0.6273	0.8892	0.1474	0.4476	0.8412	0.0373	0.9989	0.8875				
[L21]TH01	0.8533	0.9920	0.2457	0.9601	0.4013	0.9717	0.3808	0.8874	0.7765	0.0795	0.4868	0.9912	0.9995	0.7343	0.8637	0.5383	0.2762	0.6617	0.4048	0.4686			
[L22]TPOX	0.8250	0.9352	0.9911	0.8452	0.2995	0.8101	0.8587	0.2571	0.9884	0.6805	0.9644	0.3193	0.6937	0.5400	0.9925	0.9372	0.2041	0.9719	0.5617	0.8734	0.3827		
[L23]vWA	0.8446	0.5851	0.5754	0.7427	0.5839	0.9700	0.9380	0.9253	0.9779	0.6423	0.6663	0.5737	0.7782	0.9131	0.8791	0.5800	0.0540	0.9974	0.7524	0.7548	0.9538	0.8592	

Supplementary Table S6. p-values of Linkage Disequilibrium test between all pairs of 23 STR loci in Xinjiang Kumul Uygur population (after Bonferroni correction p > 0.0002).

Locus	L1	L2	L3	L4	L5	L6	L7	L8	L9	L10	L11	L12	L13	L14	L15	L16	L17	L18	L19	L20	L21	L22	L23
[L1]CSF1PO																							
[L2]D10S1248	0.2858																						
[L3]D12S391	0.9144	0.3211																					
[L4]D13S317	0.8636	0.2206	0.9350																				
[L5]D16S539	0.1822	0.3626	0.3529	0.9824																			
[L6]D18S51	0.5990	0.7477	0.9694	0.3354	0.9956																		
[L7]D19S433	0.9538	0.4970	0.2910	0.1634	0.1057	0.1844																	
[L8]D1S1656	0.1354	0.8634	0.9919	0.1238	0.9053	0.9698	0.4185																
[L9]D21S11	0.9864	0.5215	0.7412	0.2038	0.0324	0.7609	0.5342	0.3089															
[L10]D22S1045	0.1736	0.0748	0.3901	0.1039	0.0768	0.8967	0.0311	0.8033	0.5141														
[L11]D2S1338	0.2843	0.2305	0.6788	0.2426	0.4271	0.3286	0.1585	0.9659	0.6625	0.4601													
[L12]D2S441	0.7370	0.8153	0.4621	0.8070	0.5731	0.9248	0.9592	0.9988	0.7214	0.2169	0.7678												
[L13]D3S1358	0.5834	0.6911	0.6181	0.3680	0.3513	0.1111	0.3089	0.8649	0.8924	0.3756	0.0698	0.8837											
[L14]D5S818	0.5488	0.6207	0.9848	0.2761	0.2373	0.0937	0.4087	0.9465	0.7245	0.7781	0.9101	0.2083	0.3500										
[L15]D6S1043	0.3827	0.4328	0.4242	0.3453	0.5886	0.5297	0.3686	0.9328	0.3925	0.4893	0.0323	0.8969	0.0223	0.4778									
[L16]D7S820	0.6036	0.7145	0.1771	0.2696	0.0881	0.7415	0.0063	0.1002	0.1619	0.1293	0.0020	0.0883	0.4716	0.6472	0.2117								
[L17]D8S1179	0.8943	0.6715	0.7345	0.2628	0.1531	0.8931	0.1714	0.9225	0.9246	0.7632	0.5840	0.6342	0.2484	0.9484	0.2763	0.7013							
[L18]FGA	0.9651	0.7369	0.9875	0.3617	0.9969	0.9398	0.8528	0.9471	0.9989	0.8254	0.8009	0.9998	0.0853	0.2539	0.8405	0.9812	0.9911						
[L19]Penta D	0.3574	0.6425	0.8436	0.5481	0.6469	0.8625	0.2214	0.5727	0.5982	0.9576	0.7113	0.7500	0.2882	0.8407	0.9663	0.0182	0.7373	0.9918					
[L20]Penta E	0.8951	0.7617	0.9589	0.2637	0.5983	0.9924	0.0568	0.9998	0.8781	0.5893	0.3147	0.9633	0.3417	0.8771	0.8619	0.2131	0.6076	0.9991	0.8149				
[L21]TH01	0.6490	0.1451	0.6250	0.3004	0.4145	0.9987	0.2043	0.9419	0.5553	0.6596	0.7476	0.4815	0.9393	0.9355	0.6081	0.9042	0.7360	0.9585	0.9499	0.7337			
[L22]TPOX	0.8550	0.0370	0.6124	0.9555	0.7300	0.6674	0.7209	0.9398	0.9339	0.4801	0.2145	0.9921	0.9815	0.8087	0.6836	0.4931	0.8594	0.7260	0.9238	0.4705	0.7232		
[L23]vWA	0.4425	0.1581	0.6403	0.2876	0.1384	0.4999	0.1851	0.9796	0.7742	0.0393	0.7968	0.6143	0.7982	0.0944	0.5250	0.9604	0.3229	0.9834	0.8421	0.4615	0.5792	0.4485	

Supplementary Table S7. Allele frequencies of 23 autosomal STR loci included in the Huaxia Platinum system in Ningxia Wuzhong Hui population (n = 183).

Allele	CSF1PO	D10S1248	D12S391	D13S317	D16S539	D18S51	D19S433	D1S1656	D21S11	D22S1045	D2S1338	D2S441	D3S1358	D5S818	D6S1043	D7S820	D8S1179	FGA	Penta D	Penta E	TH01	TPOX	vWA
5																				0.0546			
6														0.0027					0.0082		0.1257		
7	0.0027													0.0246		0.0027			0.0082	0.0164	0.2131		
8	0.0055	0.0027		0.2623	0.0137											0.1776	0.0027		0.0519	0.0055	0.0574	0.4918	
9	0.0328			0.1230	0.2923							0.0055		0.0656	0.0055	0.0546			0.3033	0.0027	0.5164	0.1585	
9.1												0.0191				0.0082							
9.3																					0.0656		
10	0.2568			0.1448	0.0956							0.2568		0.1803	0.0301	0.1858	0.0738		0.1393	0.0410	0.0164	0.0191	
11	0.2404	0.0027		0.2842	0.3224	0.0109	0.0055	0.0683		0.2459		0.3306		0.2760	0.1038	0.3552	0.0546		0.1448	0.1093	0.0027	0.2923	
11.3												0.0355											
12	0.3825	0.0792		0.1366	0.1831	0.0383	0.0464	0.0437		0.0055		0.1913		0.2678	0.1175	0.1967	0.1284		0.1667	0.1038	0.0027	0.0383	
12.2							0.0055																
13	0.0683	0.3169		0.0355	0.0820	0.2240	0.2596	0.0792		0.0027		0.0273		0.1694	0.1421	0.0164	0.2650		0.1230	0.0628			0.0027
13.2							0.0383																
14	0.0082	0.2623		0.0137	0.0109	0.1940	0.2404	0.0902		0.0082		0.1257	0.0546	0.0137	0.1202	0.0027	0.2131		0.0492	0.1011			0.2295
14.2							0.1503																
15	0.0027	0.2377	0.0082			0.1393	0.0738	0.2814		0.2732		0.0082	0.3333		0.0055		0.1721		0.0055	0.0792			0.0383
15.2							0.1257																
15.3								0.0027															
16		0.0765	0.0082			0.1421	0.0164	0.2623		0.2459	0.0109		0.2978		0.0055		0.0738			0.0956			0.1913
16.2							0.0383																
16.3								0.0055															
17		0.0219	0.1093			0.0683		0.0738		0.1995	0.0820		0.1995		0.0301		0.0137	0.0027		0.0929			0.2486
17.3			0.0027					0.0492							0.0027								
18			0.2459			0.0710		0.0082		0.0191	0.1175		0.1093		0.2022		0.0027	0.0246		0.0710			0.2077
18.2															0.0055								
18.3								0.0301															
18.4																				0.0027			
19			0.1940			0.0383					0.1448		0.0055		0.1585			0.0301		0.0519			0.0683
19.3								0.0055															
19.4																				0.0027			
20			0.1858			0.0328					0.1202				0.0601			0.0601		0.0519			0.0109

21	0.0847	0.0082		0.0137	0.0082	0.1202	0.0301	0.0027
22	0.0902	0.0246		0.0301		0.1803	0.0137	
22.2						0.0055		
22.3					0.0027			
23	0.0492	0.0027		0.2049		0.2022	0.0027	
23.2						0.0055		
24	0.0164	0.0027		0.1749		0.1967	0.0082	
25		0.0027		0.0683		0.1011		
26	0.0055			0.0273		0.0519		
26.2						0.0055		
27			0.0027	0.0055		0.0109		
28			0.0492			0.0027		
28.2			0.0082					
29			0.2377					
29.2			0.0027					
30			0.2951					
30.2			0.0246					
30.3			0.0055					
31			0.0820					
31.2			0.0656					
32			0.0191					
32.2			0.1311					
33			0.0027					
33.2			0.0656					
34.2			0.0027					
35.2			0.0055					

Supplementary Table S8. Allele frequencies of 23 autosomal STR loci included in the Huaxia Platinum system in Sichuan Chengdu Tibetan population (n = 200).

Allele	CSF1PO	D10S1248	D12S391	D13S317	D16S539	D18S51	D19S433	D1S1656	D21S11	D22S1045	D2S1338	D2S441	D3S1358	D5S818	D6S1043	D7S820	D8S1179	FGA	Penta D	Penta E	TH01	TPOX	vWA
5																				0.0250			
6																			0.0075		0.0825		
7	0.0100			0.0025															0.0175		0.2875		
8				0.2050	0.0125									0.0075	0.0050	0.1575			0.0150	0.0025	0.0275	0.5800	
9	0.0300			0.1175	0.1925			0.0025						0.0500	0.0050	0.0525			0.3325	0.0150	0.5375	0.1200	
9.1												0.0025											
9.3																					0.0625		
10	0.2025			0.1250	0.1475							0.3200		0.2300	0.0425	0.1275	0.1025		0.1550	0.0525	0.0025	0.0050	
10.1																0.0050							
11	0.2175			0.2100	0.3375			0.0525		0.3075		0.2925		0.3725	0.1225	0.3400	0.0350		0.2175	0.0875		0.2750	
11.3												0.0150											
12	0.4700	0.0825		0.2500	0.1875	0.0225	0.0100	0.1050				0.1775		0.2000	0.1150	0.2600	0.1625		0.1175	0.1200		0.0200	
12.1	0.0025																						
12.2							0.0075																
13	0.0550	0.3950		0.0675	0.0975	0.2950	0.2675	0.1250				0.0400	0.0025	0.1250	0.1200	0.0525	0.2225		0.1100	0.0475			
13.1	0.0025																						
13.2							0.0600						0.0025										
14	0.0075	0.1975		0.0225	0.0225	0.1900	0.2125	0.0650		0.0125		0.1325	0.0125	0.0075	0.0925	0.0050	0.2325		0.0225	0.0775			0.1700
14.2							0.1850																
15	0.0025	0.2125	0.0025		0.0025	0.1200	0.0825	0.2200		0.2075		0.0200	0.3400	0.0075	0.0200		0.1750		0.0050	0.1225			0.0150
15.2							0.1275																
15.3								0.0025															
16		0.0900	0.0025			0.1100	0.0125	0.2225		0.2300	0.0050		0.3325		0.0075		0.0550			0.0850			0.2325
16.2							0.0350																
16.3								0.0050															
17		0.0200	0.1075			0.0850		0.1400		0.2025	0.0375		0.2450		0.0425		0.0150			0.0600			0.2375
17.3								0.0350															
18		0.0025	0.3050			0.0200		0.0100		0.0300	0.0725		0.0575		0.1975			0.1100		0.0750			0.2150
18.3								0.0075															
19			0.2525			0.0500				0.0100	0.1950		0.0075		0.1325			0.0325		0.0800			0.1100
19.3								0.0075							0.0025								
20			0.1325			0.0275					0.2125				0.0500			0.0400		0.0500			0.0175

20.3					0.0175			
21	0.0800	0.0375		0.0425	0.0075	0.0600	0.0275	0.0025
21.2						0.0025		
21.3					0.0200			
22	0.0500	0.0275		0.0300		0.1925	0.0350	
22.2						0.0100		
23	0.0425	0.0100		0.2300		0.2150	0.0125	
23.2						0.0200		
24	0.0225	0.0025		0.1150		0.1525	0.0100	
24.2						0.0025		
25	0.0025	0.0025		0.0575		0.0925	0.0100	
25.2						0.0125		
26				0.0025		0.0525		
26.2						0.0025		
27			0.0050					
27.2						0.0025		
28			0.0225				0.0050	
28.2			0.0100					
29			0.2900					
29.2			0.0050					
30			0.2425					
30.2			0.0175					
31			0.1350					
31.2			0.0725					
32			0.0275					
32.2			0.1100					
33			0.0025					
33.2			0.0600					

Supplementary Table S9. Allele frequencies of 23 autosomal STR loci included in the Huaxia Platinum system in Xinjiang Kumul Uygur population (n = 110).

Allele	CSF1PO	D10S1248	D12S391	D13S317	D16S539	D18S51	D19S433	D1S1656	D21S11	D22S1045	D2S1338	D2S441	D3S1358	D5S818	D6S1043	D7S820	D8S1179	FGA	Penta D	Penta E	TH01	TPOX	vWA
5																				0.0818			
6																			0.0045		0.2136		
7																			0.0227	0.0773	0.1455		
8				0.1000	0.0273			0.0045								0.2182			0.0045	0.0091	0.0591	0.4591	
9	0.0136			0.1455	0.2000							0.0045		0.0591		0.1045	0.0045		0.3591		0.4000	0.1000	
9.1												0.0318											
9.3																					0.1727		
10	0.3318			0.1136	0.0273							0.2818		0.1318	0.0455	0.2000	0.0818		0.1273	0.0636	0.0045	0.0091	
10.3																					0.0045		
11	0.2727	0.0136		0.2909	0.2364			0.1409		0.2318		0.4091		0.3318	0.2182	0.2727	0.1136		0.1955	0.1682		0.3409	
11.3												0.0227											
12	0.3364	0.0364		0.1773	0.3318	0.0955	0.0455	0.0682				0.0773		0.2682	0.1545	0.1364	0.1409		0.0864	0.1136		0.0864	
13	0.0318	0.3636		0.1091	0.1227	0.2227	0.2909	0.0864				0.0182		0.2045	0.0591	0.0682	0.3182		0.1136	0.0500		0.0045	0.0045
13.2							0.0227																
14	0.0136	0.2318		0.0636	0.0545	0.1773	0.2409	0.0636		0.0500		0.0909	0.0136	0.0045	0.0864		0.1909		0.0545	0.0955			0.1136
14.2							0.0682																
15		0.1409	0.0364			0.2136	0.0909	0.1818		0.2000		0.0636	0.3364		0.0136		0.1227		0.0318	0.1318			0.0955
15.2							0.1273																
15.3								0.0182															
16		0.1182	0.0045			0.1000	0.0591	0.2955		0.3409			0.2500				0.0227			0.0364			0.1455
16.2							0.0545																
16.3								0.0045															
17		0.0773	0.1636			0.1000		0.0500		0.1636	0.1318		0.2409		0.0455		0.0045			0.0318			0.3500
17.3			0.0136					0.0500															
18		0.0182	0.1409			0.0409		0.0045			0.1864		0.1591		0.2045					0.0500			0.1227
18.3								0.0273															
19			0.2545			0.0136				0.0136	0.1864				0.0591			0.0545		0.0364			0.1591
19.3								0.0045															
20			0.1500			0.0182					0.1091				0.0955			0.0636		0.0045			0.0091
21			0.0773			0.0136					0.0409				0.0182			0.0682		0.0091			
21.2																		0.0045					
22			0.1045			0.0045					0.0273							0.1409		0.0273			

22.2				0.0136	
23	0.0500		0.1591	0.2455	0.0045
23.2				0.0045	
24	0.0045		0.0864	0.1318	0.0091
24.2				0.0045	
25			0.0636	0.1455	
26				0.0591	
27				0.0091	
28		0.0636	0.0091	0.0500	
28.2		0.0182			
29		0.2364		0.0045	
30		0.3364			
30.2		0.0227			
31		0.0500			
31.2		0.0864			
32.2		0.1136			
33.2		0.0636			
35.2		0.0091			

Supplementary Table S10. The Fst and corresponding p values of locus-by-locus pairwise comparisons between the Ningxia Wuzhong Hui population and the other two studied populations and 47 reference populations (after Bonferroni correction $p < 0.00003$).

Locus		Sichuan-Chengdu-Tibetan	Xinjiang-Kumul-Uyghur	Hannan-Han	Sichuan-Liangshan-Yi	Sichuan-Liangshan-Tibetan	Sichuan-Han-1	Sihcuan-Han-2	Xinjiang-Uyghur-1	Tibet-Tibetan	Xinjiang-Uyghur-2	Guangdong-Han	Central-Chinese-Han	Xiamen-Han	Guizhou-Han	Xinjiang-Kazakh	Xinjiang-Uyghur-3	Ulaanbaatar-Mongolian
CSF1PO	Fst	0.0020	0.0001	-0.0004	0.0002	-0.0020	-0.0032	-0.0046	-0.0053	-0.0051	0.0006	-0.0022	-0.0020	-0.0028	-0.0021	-0.0016	0.0007	-0.0022
	p	0.2432	0.3604	0.4775	0.4054	0.6126	0.9189	0.9730	0.8198	0.8829	0.2342	0.9009	0.7478	0.9550	0.8559	0.6487	0.2793	0.7928
D1S1656	Fst	0.0048	0.0040	0.0007	-0.0014	0.0046	-0.0020	-0.0006	-0.0032	-0.0029	0.0029	0.0000	-0.0009	0.0001	-0.0015	-0.0004	0.0030	-0.0030
	p	0.0811	0.1441	0.2973	0.4955	0.0991	0.7658	0.5496	0.7207	0.7387	0.0631	0.4414	0.6036	0.3784	0.8919	0.4595	0.0270	0.9189
D2S441	Fst	-0.0015	0.0085	-0.0014	-0.0024	-0.0039	-0.0029	-0.0027	0.0010	0.0031	0.0067	-0.0023	-0.0027	-0.0005	-0.0019	0.0054	0.0071	0.0060
	p	0.4865	0.0901	0.5766	0.7027	0.8829	0.8559	0.7297	0.3153	0.2252	0.0360	0.9369	0.9640	0.4955	0.8739	0.0541	**0.0000**	0.1171
D2S1338	Fst	0.0072	0.0038	-0.0007	-0.0022	0.0027	-0.0014	-0.0021	-0.0016	0.0058	0.0023	-0.0008	-0.0019	0.0000	-0.0004	0.0021	0.0011	-0.0038
	p	**0.0000**	0.1441	0.5315	0.7297	0.2072	0.6036	0.8198	0.5676	0.1081	0.0451	0.6036	0.9189	0.4775	0.4865	0.1261	0.1712	0.9910
D3S1358	Fst	-0.0005	-0.0020	0.0017	0.0039	0.0068	-0.0014	-0.0011	-0.0046	-0.0044	-0.0020	0.0005	0.0001	-0.0016	-0.0004	-0.0029	-0.0028	0.0019
	p	0.3964	0.5225	0.2793	0.1351	0.0631	0.5225	0.4685	0.7297	0.7748	0.7387	0.3514	0.3694	0.6216	0.4324	0.9640	0.9730	0.2523
D5S818	Fst	0.0071	-0.0024	-0.0044	-0.0042	-0.0029	-0.0028	-0.0034	-0.0022	0.0028	0.0047	0.0000	-0.0008	0.0000	-0.0002	0.0093	0.0041	0.0028
	p	0.0541	0.6757	0.9369	0.9099	0.7387	0.8649	0.8829	0.6577	0.2883	0.0451	0.4955	0.5135	0.4054	0.5496	0.0180	0.0541	0.1261
D7S820	Fst	0.0009	0.0038	-0.0018	0.0041	0.0068	-0.0017	-0.0032	0.0675	0.0055	0.0019	-0.0010	-0.0010	0.0004	-0.0005	0.0083	0.0027	0.0056
	p	0.3153	0.2613	0.5856	0.1171	0.0270	0.6847	0.6937	**0.0000**	0.0901	0.1171	0.5676	0.5225	0.3423	0.5315	0.0090	0.0901	0.0631
D8S1179	Fst	-0.0022	0.0010	0.0042	-0.0040	-0.0006	-0.0009	-0.0020	-0.0060	-0.0045	-0.0016	0.0035	-0.0023	0.0048	0.0048	-0.0009	-0.0015	-0.0026
	p	0.7297	0.3153	0.0631	0.9009	0.5315	0.5856	0.7297	0.9640	0.8829	0.7297	0.0631	0.9820	0.0270	0.0090	0.5676	0.7387	0.8559
D10S1248	Fst	0.0016	0.0062	-0.0030	0.0001	-0.0018	0.0032	0.0025	-0.0031	-0.0055	0.0003	-0.0014	0.0020	0.0000	0.0003	-0.0011	0.0013	-0.0038
	p	0.1982	0.0901	0.8198	0.3964	0.6396	0.1081	0.1802	0.6306	0.8469	0.3333	0.6126	0.1712	0.3153	0.3063	0.6577	0.1892	0.9460

D12S391	Fst	0.0015	0.0047	-0.0015	-0.0042	-0.0038	-0.0029	-0.0043	-0.0017	-0.0056	0.0003	-0.0008	-0.0018	-0.0019	-0.0003	-0.0022	-0.0012	-0.0009
	p	0.2252	0.1261	0.6306	0.9640	0.9099	0.9009	0.9730	0.5676	0.9820	0.3063	0.6757	0.8919	0.8288	0.4955	0.9550	0.7297	0.5405
D13S317	Fst	0.0092	0.0159	-0.0040	-0.0018	-0.0005	-0.0007	-0.0043	-0.0023	0.0037	0.0106	-0.0016	-0.0018	-0.0009	0.0017	0.0078	0.0088	0.0027
	p	0.0270	0.0180	0.9279	0.6577	0.4775	0.4955	0.9640	0.6126	0.0901	**0.0000**	0.8469	0.7928	0.6396	0.1982	**0.0000**	**0.0000**	0.1622
D16S539	Fst	0.0033	0.0203	-0.0023	0.0041	0.0104	0.0049	0.0074	0.0060	-0.0027	0.0090	0.0022	0.0025	0.0002	0.0013	0.0105	0.0077	0.0071
	p	0.1171	**0.0000**	0.6487	0.1622	0.0270	0.0541	0.0180	0.0901	0.6487	**0.0000**	0.1351	0.0721	0.4324	0.1261	**0.0000**	0.0270	0.0270
D18S51	Fst	0.0009	0.0020	-0.0013	0.0001	0.0040	0.0008	0.0008	0.0022	-0.0028	0.0002	0.0001	-0.0009	0.0008	0.0007	0.0005	0.0012	0.0013
	p	0.2793	0.2793	0.7478	0.4865	0.0811	0.3694	0.2973	0.2883	0.7297	0.3514	0.4324	0.6216	0.1622	0.2432	0.3784	0.1712	0.2883
D19S433	Fst	-0.0032	-0.0010	-0.0041	-0.0033	-0.0041	-0.0025	-0.0031	-0.0015	-0.0052	0.0039	-0.0008	-0.0005	-0.0020	-0.0002	0.0016	0.0035	-0.0010
	p	0.9009	0.5045	0.9460	0.9099	0.9820	0.7748	0.8469	0.5496	0.9460	0.0360	0.6126	0.4234	0.9099	0.3604	0.1351	0.0180	0.5135
D21S11	Fst	0.0007	-0.0009	-0.0025	-0.0036	-0.0018	-0.0011	-0.0011	-0.0023	-0.0044	0.0004	-0.0022	-0.0012	-0.0012	-0.0017	-0.0010	0.0000	-0.0023
	p	0.3243	0.5135	0.7387	0.9189	0.7117	0.6036	0.5135	0.5676	0.9099	0.3964	0.9550	0.6306	0.7297	0.9099	0.5946	0.3874	0.7928
D22S1045	Fst	0.0004	0.0043	-0.0022	0.0000	0.0045	-0.0002	-0.0012	0.0008	-0.0013	0.0037	-0.0018	-0.0030	-0.0019	-0.0014	0.0010	0.0031	0.0030
	p	0.3784	0.1622	0.7207	0.4324	0.1261	0.4234	0.4955	0.3514	0.4865	0.0811	0.7568	0.9820	0.7387	0.6937	0.3784	0.0901	0.1081
FGA	Fst	0.0027	0.0027	-0.0004	0.0003	-0.0020	-0.0022	-0.0021	-0.0059	0.0003	-0.0009	-0.0021	-0.0024	-0.0026	-0.0017	-0.0022	-0.0019	0.0032
	p	0.1622	0.1712	0.4054	0.3514	0.6487	0.8739	0.7387	0.9820	0.4144	0.6847	0.9460	0.9730	0.9820	0.9279	0.9189	0.9189	0.0991
TH01	Fst	0.0008	0.0191	0.0011	0.0030	-0.0008	0.0019	-0.0037	0.0358	0.0081	0.0364	0.0029	0.0003	-0.0007	0.0008	0.0403	0.0306	0.0356
	p	0.2523	0.0180	0.3604	0.1622	0.3694	0.2252	0.8649	0.0090	0.1081	**0.0000**	0.1441	0.3604	0.5405	0.2072	**0.0000**	**0.0000**	**0.0000**
TPOX	Fst	0.0026	-0.0001	-0.0041	-0.0041	0.0069	-0.0027	-0.0035	-0.0025	-0.0037	0.0037	-0.0013	-0.0016	-0.0004	-0.0007	-0.0006	0.0007	-0.0010
	p	0.1712	0.3153	0.7658	0.8198	0.0811	0.7387	0.7387	0.5135	0.5496	0.0631	0.5766	0.5766	0.4324	0.4685	0.3964	0.2703	0.4775
VWA	Fst	-0.0001	0.0236	-0.0003	0.0006	0.0004	-0.0033	-0.0048	-0.0033	-0.0043	0.0048	-0.0018	-0.0024	-0.0016	-0.0001	0.0068	0.0047	0.0057
	p	0.4324	**0.0000**	0.3604	0.3333	0.3243	0.9189	0.9820	0.7117	0.7928	0.0360	0.7658	0.9189	0.7838	0.4234	0.0090	0.0270	0.0541

Continue Supplementary Table S10

Locus		Bangladeshi	Indian	Japanese	Korea-1	Korea-2	Native-American	West-Mexican-Mestizo	Monterrey-Mexican-Mestizo	South-Portuguese	Southern-Portugal-Angolan	Poland	Northern-Italy	Australian-Self-Declared-Aboriginal	Australian-Asian	Australian-Caucasian	Australian-Pure-Aboriginal	New-Zealand-East-Polynesian
CSF1PO	Fst	-0.0020	0.0028	0.0004	-0.0024	-0.0023	-0.0014	-0.0015	-0.0006	0.0014	0.0038	0.0042	0.0050	-0.0004	-0.0016	0.0020	0.0050	0.0046
	p	0.6036	0.1982	0.3153	0.8108	0.9099	0.6487	0.5766	0.4865	0.2252	0.1892	0.0631	0.0901	0.4414	0.6216	0.1982	0.0721	0.1261
D1S1656	Fst	0.0192	0.0245	0.0004	-0.0024	-0.0012	0.0206	0.0137	0.0194	0.0327	0.0328	0.0347	0.0274	0.0081	-0.0005	0.0248	0.0169	0.0224
	p	**0.0000**	**0.0000**	0.3964	0.9279	0.7117	**0.0000**	**0.0000**	**0.0000**	**0.0000**	**0.0000**	**0.0000**	**0.0000**	0.0090	0.5135	**0.0000**	**0.0000**	**0.0000**
D2S441	Fst	0.0138	0.0148	-0.0014	0.0034	0.0000	0.0651	0.0232	0.0352	0.0326	0.0210	0.0317	0.0344	0.0230	0.0008	0.0293	0.0673	0.1045
	p	**0.0000**	**0.0000**	0.6577	0.1081	0.5586	**0.0000**	**0.0000**	**0.0000**	**0.0000**	**0.0000**	**0.0000**	**0.0000**	**0.0000**	0.3153	**0.0000**	**0.0000**	**0.0000**
D2S1338	Fst	0.0036	0.0019	0.0062	-0.0012	-0.0011	0.0452	0.0149	0.0779	0.0284	0.0292	0.0398	0.0277	0.0083	-0.0006	0.0152	0.0175	0.0279
	p	0.1171	0.1892	**0.0000**	0.7838	0.7207	**0.0000**	**0.0000**	**0.0000**	**0.0000**	**0.0000**	**0.0000**	**0.0000**	**0.0000**	0.5766	**0.0000**	**0.0000**	**0.0000**
D3S1358	Fst	0.0007	-0.0021	0.0019	0.0010	0.0034	0.0464	0.0045	0.0110	0.0013	-0.0030	0.0149	0.0073	-0.0030	0.0034	0.0041	0.0016	-0.0042
	p	0.3964	0.6757	0.1712	0.1712	0.0991	**0.0000**	0.0991	**0.0000**	0.1712	0.7387	0.0090	0.0541	0.8469	0.1171	0.1261	0.2162	0.9099
D5S818	Fst	0.0022	0.0046	-0.0009	0.0003	0.0012	0.0641	0.0213	0.0304	0.0146	0.0270	0.0127	0.0180	0.0022	0.0004	0.0143	0.0017	0.0220
	p	0.1892	0.0631	0.5586	0.3333	0.2432	**0.0000**	**0.0000**	**0.0000**	**0.0000**	**0.0000**	0.0180	**0.0000**	0.1712	0.3063	**0.0000**	0.2072	**0.0000**
D7S820	Fst	0.0050	0.0072	0.0013	-0.0013	-0.0002	0.0027	0.0051	0.0098	0.0133	0.0245	0.0276	0.0155	0.0189	-0.0028	0.0169	0.0367	0.0032
	p	0.1171	0.0090	0.2613	0.6126	0.3964	0.1712	0.1081	0.0090	**0.0000**	0.0090	**0.0000**	**0.0000**	**0.0000**	0.9009	**0.0000**	**0.0000**	0.1622
D8S1179	Fst	0.0134	0.0109	0.0019	0.0013	0.0007	0.0051	0.0029	0.0056	0.0028	0.0116	0.0060	0.0006	-0.0022	0.0068	0.0013	0.0032	0.0115
	p	**0.0000**	**0.0000**	0.1622	0.1892	0.2613	0.0811	0.1982	0.0270	0.1351	**0.0000**	0.0270	0.3964	0.8919	0.0270	0.2523	0.0451	0.0180
D10S1248	Fst	0.0360	0.0215	-0.0029	-0.0008	-0.0021	0.0211	0.0047	0.0141	0.0021	0.0180	0.0142	0.0069	0.0112	-0.0004	0.0039	0.0251	0.0712
	p	**0.0000**	**0.0000**	0.9910	0.5135	0.8018	**0.0000**	0.1261	**0.0000**	0.1622	0.0180	**0.0000**	0.0631	0.0090	0.4144	0.0360	**0.0000**	**0.0000**
D12S391	Fst	-0.0017	0.0017	0.0001	-0.0020	0.0001	0.0123	0.0017	0.0035	0.0082	0.0112	0.0090	0.0067	0.0091	-0.0006	0.0094	0.0077	0.0024
	p	0.6216	0.2162	0.3243	0.7928	0.4144	**0.0000**	0.2162	0.0451	**0.0000**	0.0090	**0.0000**	0.0180	0.0090	0.5405	**0.0000**	0.0090	0.1982

D13S317	Fst	0.0078	0.0104	0.0026	-0.0031	0.0010	0.0561	0.0377	0.0339	0.0281	0.0804	0.0245	0.0315	0.0101	-0.0024	0.0268	0.0141	0.0390
	p	0.0270	**0.0000**	0.1081	0.9460	0.2252	**0.0000**	**0.0000**	**0.0000**	**0.0000**	**0.0000**	**0.0000**	**0.0000**	0.0180	0.8919	**0.0000**	**0.0000**	**0.0000**
D16S539	Fst	0.0054	0.0097	0.0197	0.0066	0.0001	0.0492	0.0472	0.0290	0.0289	0.0014	0.0425	0.0298	0.0409	0.0036	0.0264	0.0511	0.0101
	p	0.0901	**0.0000**	**0.0000**	0.0180	0.3514	**0.0000**	**0.0000**	**0.0000**	**0.0000**	0.3063	**0.0000**	**0.0000**	**0.0000**	0.1351	**0.0000**	**0.0000**	0.0360
D18S51	Fst	0.0075	0.0038	-0.0013	-0.0015	-0.0024	0.0141	0.0140	0.0129	0.0087	0.0373	0.0133	0.0052	0.0007	0.0024	0.0126	-0.0010	0.0578
	p	**0.0000**	0.0901	0.7658	0.7568	0.9640	**0.0000**	**0.0000**	**0.0000**	**0.0000**	**0.0000**	**0.0000**	0.0451	0.2703	0.1351	**0.0000**	0.6667	**0.0000**
D19S433	Fst	0.0070	0.0054	0.0087	0.0003	0.0003	0.0113	0.0142	0.0146	0.0192	0.0146	0.0295	0.0172	0.0337	-0.0008	0.0259	0.0496	0.0129
	p	0.0270	0.0721	**0.0000**	0.3514	0.2703	**0.0000**	0.0090	**0.0000**	**0.0000**	0.0090	**0.0000**	**0.0000**	**0.0000**	0.5946	**0.0000**	**0.0000**	**0.0000**
D21S11	Fst	0.0143	0.0179	-0.0008	-0.0001	-0.0004	0.1756	0.0010	-0.0001	0.0075	0.0381	0.0122	0.0048	0.0064	-0.0027	0.0101	0.0085	0.0075
	p	0.0090	**0.0000**	0.5946	0.3874	0.4865	**0.0000**	0.2613	0.4234	0.0270	**0.0000**	**0.0000**	0.0090	0.0090	0.9640	**0.0000**	0.0090	0.0360
D22S1045	Fst	0.0184	0.0229	-0.0004	-0.0006	0.0018	0.0685	0.0544	0.0809	0.0295	0.0056	0.0224	0.0254	0.0578	0.0032	0.0322	0.1146	0.0416
	p	**0.0000**	**0.0000**	0.4324	0.3874	0.2252	**0.0000**	**0.0000**	**0.0000**	**0.0000**	0.0811	**0.0000**	**0.0000**	**0.0000**	0.2252	**0.0000**	**0.0000**	**0.0000**
FGA	Fst	0.0013	0.0008	0.0001	-0.0014	-0.0014	0.0247	0.0041	0.1445	0.0074	-0.0019	0.0146	0.0039	0.0014	-0.0014	0.0092	0.0002	0.0232
	p	0.2162	0.2523	0.3604	0.6216	0.7838	**0.0000**	0.0541	**0.0000**	0.0180	0.6757	**0.0000**	0.0811	0.2432	0.8108	**0.0000**	0.4234	**0.0000**
TH01	Fst	-0.0016	0.0539	0.0166	0.0003	0.0009	0.2238	0.1143	0.1311	0.0813	0.1104	0.0050	0.1176	0.1233	0.0275	0.0006	0.1119	0.1464
	p	0.4505	**0.0000**	**0.0000**	0.2973	0.2613	**0.0000**	**0.0000**	**0.0000**	**0.0000**	**0.0000**	0.0631	**0.0000**	**0.0000**	**0.0000**	0.3243	**0.0000**	**0.0000**
TPOX	Fst	0.0137	0.0246	0.0032	-0.0015	0.0017	0.0267	0.0099	0.0126	0.0015	0.0214	0.0065	-0.0020	0.0656	0.0005	0.0019	0.1200	0.0226
	p	0.0180	**0.0000**	0.1081	0.5586	0.1982	**0.0000**	0.0180	0.0090	0.2793	**0.0000**	0.0631	0.5045	**0.0000**	0.2523	0.1351	**0.0000**	0.0090
VWA	Fst	0.0037	0.0067	-0.0008	-0.0009	-0.0022	0.0367	0.0270	0.0328	0.0123	0.0255	0.0139	0.0095	0.0145	-0.0024	0.0082	0.0242	0.0470
	p	0.1351	0.0090	0.6036	0.6216	0.9369	**0.0000**	**0.0000**	**0.0000**	**0.0000**	**0.0000**	**0.0000**	**0.0000**	**0.0000**	0.8649	0.0090	**0.0000**	**0.0000**

Continue Supplementary Table S10

Locus		New-Zealand-West-Polynesian	New-Zealand-Caucasian	North-Brazil	Northeast-Brazil	Central-West-Brazil	Southeast-Brazil	South-Brazil	Argentina-Chubut	Paraguay	Ecuador	South-Africa-Afrikaaner	South-Africa-Asian-Indian	South-Africa-Cape-Coloured	South-Africa-amaXhosa	South-Africa-amaZulu
CSF1PO	Fst	-0.0022	-0.0006	-0.0046	0.0061	-0.0001	0.0056	-0.0018	-0.0027	-0.0031	-0.0006	0.0062	0.0045	-0.0015	0.0016	0.0042
	p	0.6487	0.4685	0.9460	0.0901	0.3874	0.0991	0.5676	0.8829	0.9460	0.4955	0.0901	0.1712	0.5045	0.2793	0.1532
D1S1656	Fst	0.0241	0.0398	0.0287	0.0209	0.0337	0.0339	0.0235	0.0213	0.0251	0.0313	0.0346	0.0253	0.0081	0.0338	0.0338
	p	**0.0000**	**0.0000**	**0.0000**	**0.0000**	**0.0000**	**0.0000**	**0.0000**	**0.0000**	**0.0000**	**0.0000**	**0.0000**	**0.0000**	0.0451	**0.0000**	**0.0000**
D2S441	Fst	0.0401	0.0274	0.0363	0.0249	0.0152	0.0178	0.0179	0.0263	0.0290	0.0886	0.0261	0.0038	0.0052	0.0326	0.0368
	p	**0.0000**	**0.0000**	**0.0000**	**0.0000**	0.0090	**0.0000**	**0.0000**	**0.0000**	**0.0000**	**0.0000**	**0.0000**	0.1622	0.1171	**0.0000**	**0.0000**
D2S1338	Fst	0.0210	0.0103	0.0163	0.0215	0.0139	0.0222	0.0966	0.0222	0.0166	0.0322	0.0141	-0.0003	0.0090	0.0439	0.0373
	p	**0.0000**	**0.0000**	**0.0000**	**0.0000**	**0.0000**	**0.0000**	**0.0000**	**0.0000**	**0.0000**	**0.0000**	**0.0000**	0.4505	0.0270	**0.0000**	**0.0000**
D3S1358	Fst	-0.0001	0.0065	-0.0041	-0.0013	-0.0040	-0.0046	0.0010	0.0034	-0.0006	0.0145	0.0056	-0.0067	-0.0068	0.0091	0.0021
	p	0.4144	0.0631	0.8469	0.5496	0.9640	0.9730	0.2432	0.0631	0.4144	**0.0000**	0.1441	0.9640	0.9910	0.0360	0.1982
D5S818	Fst	0.0199	0.0100	0.0161	0.0132	0.0110	0.0120	0.0118	0.0355	0.0181	0.0492	0.0095	-0.0005	0.0017	0.0260	0.0191
	p	0.0090	0.0721	0.0090	0.0090	0.0090	0.0270	0.0090	**0.0000**	**0.0000**	**0.0000**	0.0631	0.5045	0.2973	**0.0000**	**0.0000**
D7S820	Fst	0.0292	0.0235	0.0037	0.0110	0.0085	0.0250	0.0116	0.0103	0.0083	0.0116	0.0248	0.0005	0.0126	0.0407	0.0223
	p	**0.0000**	**0.0000**	0.1261	0.0090	0.0090	**0.0000**	**0.0000**	0.0090	0.0090	**0.0000**	**0.0000**	0.3694	0.0631	**0.0000**	0.0090
D8S1179	Fst	-0.0019	0.0028	-0.0013	-0.0034	-0.0016	-0.0031	0.0001	0.0029	0.0011	0.0041	0.0078	0.0129	-0.0037	0.0089	0.0082
	p	0.5496	0.2162	0.6036	0.9369	0.6396	0.8469	0.4414	0.0631	0.2072	0.0541	0.0811	0.0451	0.8198	0.0360	0.0631
D10S1248	Fst	0.0277	0.0004	0.0051	-0.0007	-0.0008	0.0008	0.0009	0.0125	0.0157	0.0151	0.0170	0.0294	0.0056	0.0140	0.0180
	p	**0.0000**	0.2973	0.1351	0.5135	0.4595	0.3514	0.2793	0.0090	**0.0000**	**0.0000**	**0.0000**	**0.0000**	0.1351	**0.0000**	0.0090
D12S391	Fst	0.0024	0.0090	-0.0024	0.0021	0.0002	0.0012	0.0031	0.0010	0.0061	0.0145	0.0023	0.0007	0.0079	0.0011	0.0046
	p	0.1712	0.0090	0.8108	0.2072	0.3874	0.2703	0.0811	0.2342	**0.0000**	**0.0000**	0.2252	0.3694	0.0360	0.2523	0.0901

D13S317	Fst	0.0358	0.0324	0.0438	0.0503	0.0371	0.0537	0.0272	0.0431	0.0361	0.0574	0.0362	0.0032	0.0562	0.1066	0.1058
	p	**0.0000**	**0.0000**	**0.0000**	**0.0000**	**0.0000**	**0.0000**	**0.0000**	**0.0000**	**0.0000**	**0.0000**	**0.0000**	0.1982	**0.0000**	**0.0000**	**0.0000**
D16S539	Fst	0.0097	0.0292	0.0071	0.0062	0.0125	0.0138	0.0306	0.0164	0.0189	0.0180	0.0253	0.0146	0.0199	0.0059	0.0083
	p	0.0270	**0.0000**	0.0360	0.0541	0.0270	**0.0000**	**0.0000**	**0.0000**	**0.0000**	**0.0000**	**0.0000**	0.0270	**0.0000**	0.1532	0.0901
D18S51	Fst	0.0427	0.0095	0.0081	0.0122	0.0097	0.0081	0.0165	0.0111	0.0153	0.0161	0.0175	0.0051	0.0086	0.0362	0.0375
	p	**0.0000**	0.0451	0.0090	**0.0000**	**0.0000**	0.0180	**0.0000**	**0.0000**	**0.0000**	**0.0000**	0.0090	0.0901	0.0090	**0.0000**	**0.0000**
D19S433	Fst	0.0076	0.0310	0.0117	0.0085	0.0143	0.0109	0.0190	0.0211	0.0165	0.0215	0.0236	0.0017	0.0028	0.0133	0.0162
	p	0.0270	**0.0000**	**0.0000**	0.0090	**0.0000**	0.0180	**0.0000**	**0.0000**	**0.0000**	**0.0000**	**0.0000**	0.3063	0.2252	0.0090	**0.0000**
D21S11	Fst	0.0385	0.0082	0.0026	0.0114	0.0132	0.0111	0.0085	0.0013	0.0008	0.0042	0.0100	0.0022	0.0022	0.0672	0.0826
	p	**0.0000**	0.0360	0.1622	**0.0000**	**0.0000**	0.0090	0.0090	0.2162	0.2432	0.0451	0.0541	0.1892	0.2523	**0.0000**	**0.0000**
D22S1045	Fst	0.0799	0.0338	0.0417	0.0203	0.0231	0.0297	0.0276	0.0788	0.0557	0.0873	0.0256	0.0075	0.0151	0.0147	0.0070
	p	**0.0000**	**0.0000**	**0.0000**	**0.0000**	**0.0000**	**0.0000**	**0.0000**	**0.0000**	**0.0000**	**0.0000**	**0.0000**	0.0811	0.0090	0.0270	0.0811
FGA	Fst	0.0230	0.0084	0.0041	0.0016	0.0003	0.0088	-0.0002	0.0120	0.0064	0.0138	0.0036	-0.0050	-0.0026	-0.0029	0.0038
	p	**0.0000**	0.0180	0.0721	0.2072	0.4054	0.0090	0.4865	**0.0000**	**0.0000**	**0.0000**	0.1622	0.9640	0.7387	0.8559	0.1351
TH01	Fst	0.1500	0.1152	0.1187	0.0783	0.1129	0.1100	0.1210	0.1409	0.1108	0.1697	0.1390	0.0521	0.0819	0.1403	0.1223
	p	**0.0000**	**0.0000**	**0.0000**	**0.0000**	**0.0000**	**0.0000**	**0.0000**	**0.0000**	**0.0000**	**0.0000**	**0.0000**	**0.0000**	**0.0000**	**0.0000**	**0.0000**
TPOX	Fst	0.0185	-0.0012	0.0035	0.0033	-0.0001	-0.0035	0.0027	0.0081	0.0125	0.0153	0.0007	0.0309	0.0094	0.0302	0.0277
	p	0.0270	0.4685	0.1441	0.1532	0.3423	0.8108	0.2523	0.0090	0.0270	**0.0000**	0.2883	**0.0000**	0.1171	**0.0000**	0.0090
VWA	Fst	0.0045	0.0138	0.0436	0.0238	0.0200	0.0257	0.0146	0.0324	0.0268	0.0650	0.0074	0.0113	0.0151	0.0301	0.0261
	p	0.1532	0.0090	**0.0000**	**0.0000**	**0.0000**	**0.0000**	**0.0000**	**0.0000**	**0.0000**	**0.0000**	0.0811	0.0270	0.0180	**0.0000**	**0.0000**

The bold font means significant.

Supplementary Table S11. The Fst and corresponding p values of locus-by-locus pairwise comparisons between the Sichuan Chengdu Tibetan population and the other two studied populations and 47 reference populations (after Bonferroni correction p < 0.00003).

Locus		Ningxia-Wuzhong-Hui	Xinjiang-Kumul-Uyghur	Hannan-Han	Sichuan-Liangshan-Yi	Sichuan-Liangshan-Tibetan	Sichuan-Han-1	Sihcuan-Han-2	Xinjiang-Uyghur-1	Tibet-Tibetan	Xinjiang-Uyghur-2	Guangdong-Han	Central-Chinese-Han	Xiamen-Han	Guizhou-Han	Xinjiang-Kazakh	Xinjiang-Uyghur-3	Ulaanbaatar-Mongolian
CSF1PO	Fst	0.0020	0.0189	0.0144	-0.0036	0.0133	0.0023	0.0021	0.0073	-0.0050	0.0177	0.0023	0.0030	0.0068	0.0016	0.0101	0.0142	0.0084
	p	0.2432	0.0180	0.0090	0.8198	0.0090	0.1441	0.2432	0.1441	0.8919	**0.0000**	0.1261	0.1081	0.0090	0.1982	0.0180	**0.0000**	0.0541
D1S1656	Fst	0.0048	0.0072	0.0048	0.0039	0.0051	0.0100	0.0136	0.0001	0.0011	0.0068	0.0058	0.0072	0.0040	0.0060	0.0049	0.0039	0.0067
	p	0.0811	0.0721	0.0541	0.0541	0.0631	0.0090	**0.0000**	0.4234	0.2162	**0.0000**	0.0180	0.0090	0.0360	**0.0000**	**0.0000**	0.0090	0.0270
D2S441	Fst	-0.0015	0.0123	0.0098	-0.0010	-0.0005	-0.0012	0.0010	0.0091	0.0004	0.0130	0.0033	0.0029	0.0082	0.0040	0.0100	0.0143	0.0177
	p	0.4865	0.0180	0.0090	0.5045	0.4234	0.5135	0.3063	0.0631	0.3423	**0.0000**	0.0360	0.1712	**0.0000**	0.0631	0.0090	**0.0000**	0.0090
D2S1338	Fst	0.0072	0.0150	0.0094	0.0017	0.0031	0.0054	0.0034	0.0089	-0.0036	0.0046	0.0053	0.0061	0.0044	0.0051	0.0070	0.0066	0.0057
	p	**0.0000**	**0.0000**	0.0090	0.2793	0.0991	0.0090	0.0721	0.0090	0.8018	**0.0000**	**0.0000**	**0.0000**	0.0090	0.0180	0.0180	**0.0000**	0.0090
D3S1358	Fst	-0.0005	0.0045	-0.0031	0.0022	0.0036	-0.0020	-0.0028	0.0036	-0.0023	0.0004	-0.0016	-0.0007	-0.0013	-0.0018	0.0003	0.0013	0.0016
	p	0.3964	0.2072	0.7658	0.2703	0.1712	0.6487	0.6667	0.1982	0.6216	0.3063	0.7478	0.4955	0.6036	0.8018	0.3063	0.1802	0.2703
D5S818	Fst	0.0071	0.0088	0.0039	0.0010	0.0022	0.0024	0.0075	0.0156	-0.0013	0.0118	0.0009	0.0015	0.0012	0.0010	0.0111	0.0113	0.0095
	p	0.0541	0.0541	0.1712	0.2342	0.2342	0.1622	0.0360	0.0180	0.5135	**0.0000**	0.2432	0.1892	0.2613	0.2342	**0.0000**	**0.0000**	0.0090
D7S820	Fst	0.0009	0.0118	-0.0002	-0.0003	0.0050	-0.0028	-0.0024	0.0793	-0.0019	0.0097	-0.0006	-0.0015	-0.0010	-0.0006	0.0168	0.0073	0.0162
	p	0.3153	0.0180	0.3423	0.4595	0.0541	0.9009	0.6667	**0.0000**	0.5856	**0.0000**	0.5496	0.6396	0.5766	0.4685	**0.0000**	**0.0000**	0.0090
D8S1179	Fst	-0.0022	0.0070	0.0025	-0.0032	0.0009	0.0005	-0.0010	-0.0051	-0.0009	0.0038	0.0042	0.0004	0.0050	0.0057	0.0037	0.0018	0.0017
	p	0.7297	0.0721	0.1982	0.8378	0.2973	0.3063	0.5496	0.8559	0.4685	0.0541	0.0090	0.3784	0.0090	**0.0000**	0.0541	0.1351	0.2973
D10S1248	Fst	0.0016	0.0025	-0.0016	-0.0025	-0.0027	-0.0027	-0.0024	0.0093	-0.0052	0.0100	-0.0009	-0.0023	-0.0013	-0.0014	0.0034	0.0124	0.0024
	p	0.1982	0.2342	0.5946	0.7117	0.7387	0.8288	0.6577	0.0541	0.8288	**0.0000**	0.6847	0.9189	0.6757	0.7658	0.0541	**0.0000**	0.1081
D12S391	Fst	0.0015	0.0137	0.0106	0.0018	-0.0016	0.0012	-0.0008	0.0092	-0.0018	0.0050	0.0061	0.0010	0.0045	0.0052	0.0054	0.0060	0.0063

	p	0.2252	0.0180	0.0090	0.2342	0.6306	0.2252	0.4324	0.0541	0.5946	0.0090	**0.0000**	0.2432	0.0270	**0.0000**	**0.0000**	**0.0000**	0.0631
D13S317	Fst	0.0092	0.0086	0.0080	0.0031	-0.0001	0.0070	0.0064	0.0009	-0.0032	0.0048	0.0077	0.0073	0.0072	0.0151	-0.0013	0.0014	-0.0026
	p	0.0270	0.0451	0.0360	0.1171	0.4054	0.0090	0.0541	0.3604	0.7117	0.0090	**0.0000**	**0.0000**	0.0090	**0.0000**	0.7478	0.1892	0.8649
D16S539	Fst	0.0033	0.0215	0.0038	0.0041	-0.0007	0.0151	0.0205	-0.0033	-0.0063	0.0050	0.0047	0.0100	0.0076	0.0058	0.0096	0.0027	0.0112
	p	0.1171	**0.0000**	0.1441	0.0901	0.4595	**0.0000**	**0.0000**	0.7117	0.9820	0.0360	0.0270	**0.0000**	**0.0000**	0.0180	**0.0000**	0.1081	**0.0000**
D18S51	Fst	0.0009	0.0070	0.0080	-0.0019	-0.0018	0.0090	0.0086	0.0109	-0.0044	0.0091	0.0068	0.0032	0.0099	0.0100	0.0082	0.0112	0.0087
	p	0.2793	0.0811	**0.0000**	0.7658	0.7658	0.0090	0.0090	0.0180	0.9460	**0.0000**	**0.0000**	0.0270	**0.0000**	**0.0000**	0.0090	**0.0000**	0.0090
D19S433	Fst	-0.0032	0.0045	-0.0016	-0.0009	-0.0029	0.0005	0.0004	0.0048	-0.0044	0.0107	0.0030	0.0037	0.0017	0.0026	0.0076	0.0106	0.0046
	p	0.9009	0.0991	0.6487	0.5225	0.8919	0.3243	0.2793	0.1351	0.8288	**0.0000**	0.0631	0.0631	0.1081	0.0631	**0.0000**	**0.0000**	0.0811
D21S11	Fst	0.0007	-0.0069	-0.0027	-0.0017	-0.0001	-0.0018	-0.0012	0.0016	0.0021	0.0054	0.0010	-0.0006	0.0023	0.0010	0.0037	0.0061	0.0029
	p	0.3243	0.9910	0.8018	0.7207	0.4505	0.8378	0.5405	0.1892	0.2703	0.0180	0.2613	0.4685	0.0901	0.2523	0.0451	0.0090	0.1351
D22S1045	Fst	0.0004	0.0075	0.0079	-0.0052	-0.0025	0.0109	0.0066	0.0107	-0.0067	0.0122	0.0079	0.0029	0.0072	0.0091	0.0032	0.0112	-0.0018
	p	0.3784	0.0721	0.0270	0.9910	0.6487	0.0090	0.0811	0.0631	0.9730	**0.0000**	0.0180	0.1171	0.0090	**0.0000**	0.1171	**0.0000**	0.6126
FGA	Fst	0.0027	0.0071	0.0057	0.0030	0.0036	0.0055	0.0037	0.0050	-0.0020	0.0126	0.0037	0.0039	0.0043	0.0054	0.0089	0.0092	0.0167
	p	0.1622	0.0451	**0.0000**	0.1532	0.0811	0.0270	0.0631	0.0721	0.6126	**0.0000**	0.0180	0.0180	0.0270	0.0090	0.0090	**0.0000**	**0.0000**
TH01	Fst	0.0008	0.0399	0.0049	-0.0021	-0.0023	-0.0010	-0.0020	0.0473	-0.0015	0.0448	0.0019	-0.0016	-0.0017	-0.0007	0.0514	0.0417	0.0351
	p	0.2523	**0.0000**	0.1622	0.6216	0.6667	0.5135	0.5856	**0.0000**	0.4685	**0.0000**	0.2252	0.7027	0.7027	0.5766	**0.0000**	**0.0000**	**0.0000**
TPOX	Fst	0.0026	0.0116	-0.0013	0.0011	0.0023	-0.0014	-0.0022	0.0053	0.0120	0.0014	0.0003	0.0020	0.0002	0.0026	-0.0006	0.0043	-0.0025
	p	0.1712	0.0541	0.4505	0.2883	0.1712	0.4865	0.6577	0.1351	0.0631	0.2613	0.2973	0.1351	0.3784	0.1532	0.4324	0.0270	0.7387
VWA	Fst	-0.0001	0.0200	0.0106	-0.0023	0.0017	-0.0003	-0.0005	-0.0036	0.0001	0.0007	0.0059	0.0025	0.0066	0.0075	0.0019	0.0008	0.0013
	p	0.4324	**0.0000**	0.0090	0.7387	0.1892	0.4775	0.4324	0.7027	0.3243	0.2883	**0.0000**	0.1171	0.0090	**0.0000**	0.1622	0.1982	0.2162

Continue Supplementary Table S11

Locus		Bangladeshi	Indian	Japanese	Korea-1	Korea-2	Native-American	West-Mexican-Mestizo	Monterrey-Mexican-Mestizo	South-Portuguese	Southern-Portugal-Angolan	Poland	Northern-Italy	Australian-Self-Declared-Aboriginal	Australian-Asian	Australian-Caucasian	Australian-Pure-Aboriginal	New-Zealand-East-Polynesian
CSF1PO	Fst	-0.0001	0.0085	-0.0009	-0.0002	0.0014	-0.0003	0.0078	0.0107	0.0184	0.0241	0.0253	0.0260	0.0067	0.0091	0.0194	0.0175	0.0187
	p	0.3243	0.0360	0.5766	0.4234	0.1712	0.4595	0.0451	0.0090	**0.0000**	**0.0000**	**0.0000**	**0.0000**	0.0631	0.0180	**0.0000**	**0.0000**	**0.0000**
D1S1656	Fst	0.0124	0.0161	0.0046	0.0034	0.0045	0.0246	0.0096	0.0189	0.0245	0.0360	0.0265	0.0194	0.0024	0.0047	0.0194	0.0061	0.0303
	p	**0.0000**	**0.0000**	0.0090	0.0721	0.0270	**0.0000**	**0.0000**	**0.0000**	**0.0000**	**0.0000**	**0.0000**	**0.0000**	0.1351	0.0180	**0.0000**	0.0090	**0.0000**
D2S441	Fst	0.0198	0.0179	0.0022	0.0153	0.0087	0.0453	0.0114	0.0197	0.0374	0.0358	0.0351	0.0426	0.0097	0.0046	0.0315	0.0439	0.0800
	p	**0.0000**	**0.0000**	0.1081	**0.0000**	**0.0000**	**0.0000**	0.0180	**0.0000**	**0.0000**	**0.0000**	**0.0000**	**0.0000**	0.0090	0.0721	**0.0000**	**0.0000**	**0.0000**
D2S1338	Fst	0.0040	0.0086	0.0158	0.0097	0.0099	0.0326	0.0147	0.0721	0.0410	0.0361	0.0611	0.0452	0.0148	0.0071	0.0268	0.0165	0.0431
	p	0.0541	**0.0000**	**0.0000**	**0.0000**	**0.0000**	**0.0000**	**0.0000**	**0.0000**	**0.0000**	**0.0000**	**0.0000**	**0.0000**	**0.0000**	**0.0000**	**0.0000**	**0.0000**	**0.0000**
D3S1358	Fst	0.0025	0.0006	0.0017	0.0025	0.0060	0.0529	0.0099	0.0186	0.0125	-0.0016	0.0318	0.0182	0.0027	-0.0003	0.0165	0.0078	-0.0029
	p	0.2162	0.2162	0.1892	0.1261	0.0360	**0.0000**	0.0270	**0.0000**	**0.0000**	0.5946	**0.0000**	0.0090	0.1261	0.4324	**0.0000**	0.0180	0.6757
D5S818	Fst	0.0079	0.0067	0.0049	0.0003	0.0002	0.0453	0.0268	0.0258	0.0341	0.0678	0.0314	0.0407	0.0098	0.0009	0.0295	0.0101	0.0372
	p	0.0270	0.0180	0.0631	0.3514	0.4144	**0.0000**	**0.0000**	**0.0000**	**0.0000**	**0.0000**	**0.0000**	**0.0000**	0.0090	0.2342	**0.0000**	0.0090	**0.0000**
D7S820	Fst	0.0125	0.0179	0.0049	-0.0009	-0.0017	0.0045	0.0144	0.0177	0.0224	0.0422	0.0409	0.0274	0.0288	0.0009	0.0267	0.0542	0.0037
	p	0.0270	**0.0000**	0.0541	0.6667	0.8108	0.0541	**0.0000**	**0.0000**	**0.0000**	**0.0000**	**0.0000**	**0.0000**	**0.0000**	0.2703	**0.0000**	**0.0000**	0.1261
D8S1179	Fst	0.0087	0.0084	0.0028	0.0021	0.0017	0.0048	0.0078	0.0100	0.0072	0.0091	0.0095	0.0068	0.0015	0.0072	0.0049	0.0067	0.0130
	p	0.0180	**0.0000**	0.0631	0.0991	0.2162	0.0180	0.0090	**0.0000**	**0.0000**	0.0090	0.0090	0.0180	0.2432	**0.0000**	0.0270	0.0270	**0.0000**
D10S1248	Fst	0.0627	0.0419	0.0031	-0.0021	0.0007	0.0522	0.0214	0.0375	0.0143	0.0257	0.0376	0.0166	0.0288	-0.0018	0.0177	0.0392	0.0388
	p	**0.0000**	**0.0000**	0.0451	0.7568	0.2973	**0.0000**	0.0090	**0.0000**	**0.0000**	**0.0000**	**0.0000**	**0.0000**	**0.0000**	0.7117	**0.0000**	**0.0000**	**0.0000**
D12S391	Fst	0.0024	0.0089	0.0024	-0.0003	-0.0009	0.0183	0.0109	0.0102	0.0204	0.0172	0.0238	0.0148	0.0178	0.0092	0.0245	0.0109	0.0105
	p	0.2162	**0.0000**	0.1171	0.4865	0.5676	**0.0000**	**0.0000**	**0.0000**	**0.0000**	**0.0000**	**0.0000**	**0.0000**	**0.0000**	**0.0000**	**0.0000**	**0.0000**	0.0090

D13S317	Fst	-0.0027	-0.0017	0.0012	0.0063	0.0032	0.0360	0.0133	0.0129	0.0143	0.0462	0.0223	0.0106	0.0090	0.0057	0.0102	0.0230	0.0091
	p	0.8108	0.6847	0.1532	0.0180	0.0811	**0.0000**	**0.0000**	**0.0000**	**0.0000**	**0.0000**	**0.0000**	**0.0000**	0.0270	0.0270	0.0180	**0.0000**	0.0270
D16S539	Fst	0.0001	0.0000	0.0314	0.0112	0.0065	0.0240	0.0210	0.0092	0.0176	-0.0035	0.0296	0.0189	0.0200	-0.0004	0.0142	0.0238	0.0061
	p	0.3604	0.4595	**0.0000**	0.0090	0.0090	**0.0000**	**0.0000**	**0.0000**	**0.0000**	0.8288	**0.0000**	**0.0000**	**0.0000**	0.5135	**0.0000**	**0.0000**	0.1171
D18S51	Fst	0.0215	0.0158	0.0053	0.0033	0.0036	0.0256	0.0280	0.0253	0.0206	0.0578	0.0304	0.0150	0.0091	0.0147	0.0246	0.0055	0.0713
	p	**0.0000**	**0.0000**	**0.0000**	0.0541	0.0270	**0.0000**	**0.0000**	**0.0000**	**0.0000**	**0.0000**	**0.0000**	**0.0000**	**0.0000**	**0.0000**	**0.0000**	0.0180	**0.0000**
D19S433	Fst	0.0127	0.0126	0.0175	0.0054	0.0043	0.0139	0.0196	0.0203	0.0305	0.0200	0.0421	0.0288	0.0467	0.0051	0.0375	0.0658	0.0183
	p	**0.0000**	**0.0000**	**0.0000**	0.0270	0.0360	**0.0000**	**0.0000**	**0.0000**	**0.0000**	**0.0000**	**0.0000**	**0.0000**	**0.0000**	0.0360	**0.0000**	**0.0000**	**0.0000**
D21S11	Fst	0.0238	0.0236	0.0058	0.0102	0.0088	0.1773	0.0103	0.0075	0.0170	0.0427	0.0191	0.0100	0.0107	0.0009	0.0148	0.0116	0.0130
	p	**0.0000**	**0.0000**	0.0090	**0.0000**	0.0090	**0.0000**	0.0090	**0.0000**	**0.0000**	**0.0000**	**0.0000**	0.0090	**0.0000**	0.2613	**0.0000**	**0.0000**	**0.0000**
D22S1045	Fst	0.0214	0.0373	0.0125	0.0118	0.0151	0.1048	0.0884	0.1198	0.0551	0.0126	0.0441	0.0506	0.0939	0.0197	0.0586	0.1600	0.0432
	p	**0.0000**	**0.0000**	**0.0000**	**0.0000**	**0.0000**	**0.0000**	**0.0000**	**0.0000**	**0.0000**	0.0090	**0.0000**	**0.0000**	**0.0000**	**0.0000**	**0.0000**	**0.0000**	**0.0000**
FGA	Fst	0.0091	0.0130	0.0077	0.0048	0.0044	0.0324	0.0117	0.1441	0.0189	0.0036	0.0272	0.0166	0.0100	0.0047	0.0206	0.0073	0.0334
	p	0.0090	**0.0000**	0.0090	0.0180	0.0360	**0.0000**	**0.0000**	**0.0000**	**0.0000**	0.0631	**0.0000**	**0.0000**	**0.0000**	0.0180	**0.0000**	**0.0000**	**0.0000**
TH01	Fst	-0.0015	0.0803	0.0249	0.0062	0.0059	0.2186	0.1273	0.1379	0.1006	0.1224	0.0058	0.1395	0.1455	0.0397	-0.0022	0.1333	0.1608
	p	0.4955	**0.0000**	**0.0000**	0.0541	0.0270	**0.0000**	**0.0000**	**0.0000**	**0.0000**	**0.0000**	0.0360	**0.0000**	**0.0000**	**0.0000**	0.7117	**0.0000**	**0.0000**
TPOX	Fst	0.0418	0.0554	0.0158	0.0075	0.0084	0.0384	0.0147	0.0130	0.0015	0.0493	-0.0005	0.0051	0.1074	-0.0017	0.0016	0.1706	0.0389
	p	**0.0000**	**0.0000**	**0.0000**	0.0270	0.0180	**0.0000**	0.0090	0.0090	0.2252	**0.0000**	0.3153	0.0721	**0.0000**	0.5766	0.2072	**0.0000**	**0.0000**
VWA	Fst	-0.0010	0.0045	0.0020	0.0025	0.0010	0.0201	0.0166	0.0184	0.0091	0.0201	0.0112	0.0050	0.0053	0.0030	0.0047	0.0103	0.0561
	p	0.4955	0.0811	0.1622	0.0991	0.2072	**0.0000**	**0.0000**	**0.0000**	**0.0000**	**0.0000**	**0.0000**	0.0811	0.0180	0.1441	0.0270	**0.0000**	**0.0000**

Continue Supplementary Table S11

Locus		New-Zealand-West-Polynesian	New-Zealand-Caucasian	North-Brazil	Northeast-Brazil	Central-West-Brazil	Southeast-Brazil	South-Brazil	Argentina-Chubut	Paraguay	Ecuador	South-Africa-Afrikaaner	South-Africa-Asian-Indian	South-Africa-Cape-Coloured	South-Africa-amaXhosa	South-Africa-amaZulu
CSF1PO	Fst	0.0091	0.0162	0.0000	0.0267	0.0156	0.0271	0.0088	0.0084	0.0060	0.0092	0.0320	0.0043	0.0129	0.0201	0.0217
	p	0.0451	**0.0000**	0.4505	0.0090	**0.0000**	**0.0000**	0.0180	0.0090	0.0451	**0.0000**	**0.0000**	0.2072	0.0090	0.0180	0.0090
D1S1656	Fst	0.0174	0.0289	0.0274	0.0174	0.0274	0.0256	0.0204	0.0167	0.0204	0.0260	0.0248	0.0190	0.0036	0.0354	0.0372
	p	**0.0000**	**0.0000**	**0.0000**	**0.0000**	**0.0000**	**0.0000**	**0.0000**	**0.0000**	**0.0000**	**0.0000**	**0.0000**	**0.0000**	0.1892	**0.0000**	**0.0000**
D2S441	Fst	0.0268	0.0283	0.0215	0.0274	0.0165	0.0195	0.0182	0.0209	0.0186	0.0604	0.0317	0.0076	0.0138	0.0514	0.0544
	p	0.0090	**0.0000**	**0.0000**	**0.0000**	0.0090	**0.0000**	**0.0000**	**0.0000**	**0.0000**	**0.0000**	**0.0000**	0.0721	0.0360	**0.0000**	**0.0000**
D2S1338	Fst	0.0414	0.0232	0.0220	0.0323	0.0232	0.0332	0.1105	0.0294	0.0267	0.0283	0.0253	0.0023	0.0105	0.0447	0.0358
	p	**0.0000**	**0.0000**	**0.0000**	**0.0000**	**0.0000**	**0.0000**	**0.0000**	**0.0000**	**0.0000**	**0.0000**	**0.0000**	0.2072	0.0180	**0.0000**	**0.0000**
D3S1358	Fst	-0.0023	0.0245	-0.0008	0.0087	0.0029	-0.0006	0.0121	0.0107	0.0081	0.0200	0.0181	-0.0005	-0.0014	0.0104	0.0050
	p	0.4775	0.0090	0.3514	0.0631	0.2072	0.4324	0.0180	**0.0000**	0.0180	**0.0000**	0.0180	0.4234	0.4054	0.0270	0.1892
D5S818	Fst	0.0427	0.0259	0.0260	0.0386	0.0316	0.0357	0.0297	0.0327	0.0283	0.0391	0.0301	0.0074	0.0256	0.0621	0.0514
	p	**0.0000**	**0.0000**	**0.0000**	**0.0000**	**0.0000**	**0.0000**	**0.0000**	**0.0000**	**0.0000**	**0.0000**	**0.0000**	0.1351	0.0090	**0.0000**	**0.0000**
D7S820	Fst	0.0205	0.0285	0.0133	0.0216	0.0170	0.0337	0.0168	0.0191	0.0124	0.0105	0.0362	0.0083	0.0249	0.0594	0.0428
	p	0.0090	**0.0000**	0.0090	**0.0000**	**0.0000**	**0.0000**	**0.0000**	**0.0000**	0.0090	**0.0000**	**0.0000**	0.0991	**0.0000**	**0.0000**	**0.0000**
D8S1179	Fst	0.0058	0.0053	-0.0008	-0.0008	0.0001	-0.0008	0.0033	0.0097	0.0035	0.0050	0.0140	0.0102	-0.0005	0.0120	0.0095
	p	0.0541	0.0901	0.5315	0.5586	0.4865	0.5225	0.1081	0.0090	0.0631	0.0360	0.0270	0.0180	0.4685	0.0270	0.0270
D10S1248	Fst	0.0096	0.0070	0.0204	0.0109	0.0144	0.0106	0.0177	0.0376	0.0391	0.0400	0.0288	0.0501	0.0226	0.0233	0.0300
	p	0.0360	0.0451	**0.0000**	0.0090	0.0090	0.0090	0.0090	**0.0000**	**0.0000**	**0.0000**	0.0090	**0.0000**	**0.0000**	**0.0000**	**0.0000**
D12S391	Fst	0.0050	0.0235	0.0034	0.0170	0.0092	0.0128	0.0133	0.0056	0.0228	0.0252	0.0153	0.0059	0.0174	0.0051	0.0079
	p	0.1261	**0.0000**	0.0991	**0.0000**	**0.0000**	**0.0000**	**0.0000**	**0.0000**	**0.0000**	**0.0000**	**0.0000**	0.1261	0.0090	0.1261	0.0541

D13S317	Fst	0.0213	0.0195	0.0137	0.0203	0.0123	0.0215	0.0076	0.0172	0.0095	0.0330	0.0099	-0.0046	0.0197	0.0579	0.0619
	p	**0.0000**	**0.0000**	**0.0000**	**0.0000**	**0.0000**	**0.0000**	0.0180	**0.0000**	**0.0000**	**0.0000**	0.0360	0.8829	**0.0000**	**0.0000**	**0.0000**
D16S539	Fst	0.0015	0.0151	0.0046	0.0046	0.0053	0.0056	0.0182	0.0077	0.0111	0.0080	0.0142	0.0028	0.0080	-0.0010	-0.0030
	p	0.2162	0.0090	0.1532	0.0541	0.0631	0.0360	**0.0000**	0.0090	**0.0000**	0.0180	0.0360	0.1982	0.0270	0.4955	0.6667
D18S51	Fst	0.0579	0.0269	0.0195	0.0280	0.0257	0.0235	0.0335	0.0252	0.0285	0.0309	0.0275	0.0189	0.0217	0.0530	0.0575
	p	**0.0000**	**0.0000**	**0.0000**	**0.0000**	**0.0000**	**0.0000**	**0.0000**	**0.0000**	**0.0000**	**0.0000**	**0.0000**	0.0090	**0.0000**	**0.0000**	**0.0000**
D19S433	Fst	0.0051	0.0429	0.0174	0.0165	0.0220	0.0214	0.0295	0.0291	0.0258	0.0246	0.0338	0.0103	0.0095	0.0191	0.0216
	p	0.0991	**0.0000**	**0.0000**	**0.0000**	**0.0000**	**0.0000**	**0.0000**	**0.0000**	**0.0000**	**0.0000**	**0.0000**	0.0270	0.0180	**0.0000**	**0.0000**
D21S11	Fst	0.0387	0.0166	0.0100	0.0186	0.0254	0.0206	0.0119	0.0123	0.0122	0.0121	0.0175	0.0091	0.0133	0.0752	0.0833
	p	**0.0000**	0.0090	0.0090	**0.0000**	**0.0000**	**0.0000**	**0.0000**	**0.0000**	**0.0000**	**0.0000**	**0.0000**	0.0631	0.0090	**0.0000**	**0.0000**
D22S1045	Fst	0.1043	0.0562	0.0715	0.0429	0.0470	0.0556	0.0530	0.1190	0.0891	0.1267	0.0459	0.0176	0.0287	0.0193	0.0019
	p	**0.0000**	**0.0000**	**0.0000**	**0.0000**	**0.0000**	**0.0000**	**0.0000**	**0.0000**	**0.0000**	**0.0000**	**0.0000**	**0.0000**	**0.0000**	**0.0000**	0.2072
FGA	Fst	0.0309	0.0196	0.0153	0.0128	0.0096	0.0203	0.0100	0.0226	0.0178	0.0224	0.0141	0.0061	0.0074	0.0040	0.0077
	p	**0.0000**	**0.0000**	**0.0000**	**0.0000**	**0.0000**	**0.0000**	**0.0000**	**0.0000**	**0.0000**	**0.0000**	**0.0000**	0.0541	0.0360	0.0991	0.0180
TH01	Fst	0.1564	0.1382	0.1402	0.0940	0.1286	0.1257	0.1420	0.1579	0.1261	0.1785	0.1594	0.0824	0.1093	0.1527	0.1333
	p	**0.0000**	**0.0000**	**0.0000**	**0.0000**	**0.0000**	**0.0000**	**0.0000**	**0.0000**	**0.0000**	**0.0000**	**0.0000**	**0.0000**	**0.0000**	**0.0000**	**0.0000**
TPOX	Fst	0.0519	-0.0022	0.0132	0.0113	0.0137	0.0054	0.0116	0.0128	0.0271	0.0148	-0.0039	0.0685	0.0366	0.0633	0.0605
	p	**0.0000**	0.6126	0.0090	**0.0000**	0.0090	0.0901	0.0541	**0.0000**	**0.0000**	0.0090	0.6036	**0.0000**	**0.0000**	**0.0000**	**0.0000**
VWA	Fst	0.0117	0.0105	0.0325	0.0152	0.0135	0.0200	0.0089	0.0204	0.0163	0.0466	0.0014	0.0051	0.0156	0.0321	0.0189
	p	0.0270	0.0360	**0.0000**	**0.0000**	**0.0000**	**0.0000**	0.0360	**0.0000**	**0.0000**	**0.0000**	0.2973	0.1441	**0.0000**	**0.0000**	0.0090

The bold font means significant.

Supplementary Table S12. The Fst and corresponding p values of locus-by-locus pairwise comparisons between the Xinjiang Kumul Uyghur population and the other two studied populations and 47 reference populations (after Bonferroni correction p < 0.00003).

Locus		Ningxia-Wuzhong-Hui	Sichuan-Chengdu-Tibetan	Hannan-Han	Sichuan-Liangshan-Yi	Sichuan-Liangshan-Tibetan	Sichuan-Han-1	Sihcuan-Han-2	Xinjiang-Uyghur-1	Tibet-Tibetan	Xinjiang-Uyghur-2	Guangdong-Han	Central-Chinese-Han	Xiamen-Han	Guizhou-Han	Xinjiang-Kazakh	Xinjiang-Uyghur-3	Ulaanbaatar-Mongolian
CSF1PO	Fst	0.0001	0.0189	0.0007	0.0163	-0.0026	0.0064	0.0029	-0.0046	0.0086	-0.0025	0.0080	0.0083	0.0024	0.0077	0.0009	0.0016	0.0016
	p	0.3604	0.0180	0.3063	0.0180	0.5225	0.0631	0.2162	0.6306	0.1171	0.6577	0.0631	0.0451	0.1892	0.0270	0.3153	0.2523	0.2523
D1S1656	Fst	0.0040	0.0072	0.0144	0.0067	0.0044	0.0147	0.0201	0.0090	0.0103	0.0075	0.0130	0.0126	0.0110	0.0089	0.0042	0.0071	0.0069
	p	0.1441	0.0721	**0.0000**	0.0811	0.1261	**0.0000**	**0.0000**	0.0451	0.0360	0.0270	**0.0000**	**0.0000**	0.0090	**0.0000**	0.0811	0.0180	0.0631
D2S441	Fst	0.0085	0.0123	0.0080	0.0078	0.0059	0.0140	0.0136	0.0058	0.0117	0.0047	0.0077	0.0074	0.0171	0.0105	-0.0002	0.0025	0.0046
	p	0.0901	0.0180	0.0991	0.0721	0.1081	**0.0000**	0.0270	0.1802	0.1081	0.0991	0.0360	0.0901	0.0090	**0.0000**	0.4955	0.1532	0.1351
D2S1338	Fst	0.0038	0.0150	0.0036	0.0029	0.0139	0.0042	0.0046	-0.0036	0.0108	0.0011	0.0050	0.0050	0.0031	0.0074	0.0004	0.0026	0.0056
	p	0.1441	**0.0000**	0.1892	0.1351	0.0180	0.1261	0.1261	0.7387	0.0451	0.2162	0.0180	0.0541	0.1351	0.0090	0.4234	0.0991	0.0811
D3S1358	Fst	-0.0020	0.0045	0.0060	0.0165	0.0211	0.0079	0.0083	0.0003	-0.0051	0.0029	0.0085	0.0090	0.0028	0.0068	0.0032	0.0021	0.0096
	p	0.5225	0.2072	0.0991	0.0090	0.0090	0.1441	0.0541	0.3784	0.7387	0.1171	0.0180	0.0270	0.1532	0.0451	0.0901	0.2883	0.0541
D5S818	Fst	-0.0024	0.0088	0.0000	-0.0019	-0.0028	-0.0012	-0.0038	-0.0068	-0.0006	-0.0017	0.0020	-0.0004	0.0024	0.0027	0.0009	-0.0013	-0.0039
	p	0.6757	0.0541	0.3784	0.4865	0.6306	0.4595	0.7297	0.8378	0.3874	0.5766	0.2162	0.3964	0.2432	0.1802	0.3514	0.5225	0.7568
D7S820	Fst	0.0038	0.0118	0.0102	0.0054	0.0077	0.0061	0.0068	0.0436	0.0093	-0.0022	0.0079	0.0096	0.0137	0.0114	-0.0030	-0.0007	-0.0016
	p	0.2613	0.0180	0.0811	0.1261	0.0541	0.0631	0.0991	**0.0000**	0.0721	0.7297	0.0360	0.0180	**0.0000**	0.0180	0.7748	0.4324	0.5315
D8S1179	Fst	0.0010	0.0070	0.0159	0.0055	0.0045	0.0058	0.0043	-0.0001	0.0059	0.0001	0.0100	0.0029	0.0084	0.0111	-0.0020	0.0003	-0.0035
	p	0.3153	0.0721	0.0090	0.1171	0.0991	0.0811	0.1171	0.3964	0.0901	0.4234	**0.0000**	0.1532	0.0360	**0.0000**	0.6306	0.2793	0.8559
D10S1248	Fst	0.0062	0.0025	0.0003	-0.0040	-0.0018	0.0020	0.0022	0.0042	-0.0010	0.0094	0.0052	0.0031	0.0017	0.0068	0.0051	0.0092	0.0048
	p	0.0901	0.2342	0.3423	0.8198	0.5045	0.2613	0.2432	0.1892	0.4865	**0.0000**	0.1532	0.1171	0.2342	0.0541	0.1441	0.0090	0.1622
D12S391	Fst	0.0047	0.0137	0.0045	0.0005	0.0054	0.0041	0.0050	-0.0003	0.0008	0.0053	0.0037	0.0070	0.0080	0.0062	0.0035	0.0054	0.0048

	p	0.1261	0.0180	0.0541	0.4324	0.0541	0.1441	0.1261	0.4324	0.3333	0.0631	0.1351	0.0270	0.0360	**0.0000**	0.1261	0.0901	0.1081
D13S317	Fst	0.0159	0.0086	0.0182	0.0180	0.0119	0.0228	0.0169	0.0035	0.0180	-0.0009	0.0218	0.0180	0.0228	0.0358	0.0056	0.0031	0.0117
	p	0.0180	0.0451	**0.0000**	0.0090	0.0090	**0.0000**	0.0090	0.2162	0.0090	0.5946	**0.0000**	**0.0000**	**0.0000**	**0.0000**	0.0451	0.1532	0.0270
D16S539	Fst	0.0203	0.0215	0.0145	0.0003	0.0431	0.0137	0.0166	0.0087	0.0169	0.0041	0.0113	0.0143	0.0166	0.0108	0.0099	0.0074	0.0106
	p	**0.0000**	**0.0000**	0.0451	0.3514	**0.0000**	0.0180	0.0090	0.0721	0.0721	0.0901	**0.0000**	**0.0000**	0.0090	0.0090	0.0090	0.0360	0.0090
D18S51	Fst	0.0020	0.0070	-0.0029	0.0070	0.0066	0.0022	0.0027	-0.0007	0.0066	0.0019	0.0012	0.0011	0.0006	0.0035	0.0040	0.0023	0.0065
	p	0.2793	0.0811	0.7838	0.0541	0.0721	0.2072	0.1532	0.5135	0.0451	0.2342	0.2613	0.2162	0.2973	0.0901	0.1081	0.1982	0.0270
D19S433	Fst	-0.0010	0.0045	-0.0015	-0.0042	0.0002	-0.0014	-0.0019	-0.0025	-0.0030	-0.0001	-0.0009	-0.0012	-0.0007	-0.0004	0.0007	-0.0016	-0.0001
	p	0.5045	0.0991	0.5405	0.8829	0.3423	0.5586	0.5856	0.5766	0.5856	0.3694	0.5766	0.5946	0.4685	0.4865	0.2883	0.5946	0.4685
D21S11	Fst	-0.0009	-0.0069	-0.0042	-0.0032	-0.0019	-0.0034	-0.0028	0.0004	0.0003	0.0039	-0.0007	-0.0022	0.0009	-0.0007	0.0021	0.0049	0.0014
	p	0.5135	0.9910	0.9009	0.7748	0.6396	0.8288	0.6757	0.3063	0.4054	0.0541	0.5135	0.7658	0.3153	0.5315	0.2793	0.0991	0.2793
D22S1045	Fst	0.0043	0.0075	0.0001	0.0066	0.0034	0.0136	0.0076	0.0039	0.0016	0.0158	0.0083	0.0066	0.0039	0.0087	0.0012	0.0070	0.0010
	p	0.1622	0.0721	0.3423	0.1261	0.2432	0.0360	0.0991	0.1351	0.3784	**0.0000**	0.0451	0.0721	0.0991	0.0270	0.2613	0.0360	0.3964
FGA	Fst	0.0027	0.0071	0.0040	-0.0016	0.0016	0.0056	0.0028	0.0044	0.0037	0.0076	0.0029	0.0026	0.0023	0.0024	0.0051	0.0067	0.0101
	p	0.1712	0.0451	0.1532	0.6306	0.2432	0.0451	0.1982	0.2072	0.1802	0.0090	0.1351	0.1081	0.1622	0.1892	0.0541	0.0451	0.0270
TH01	Fst	0.0191	0.0399	0.0317	0.0368	0.0336	0.0334	0.0257	0.0127	0.0428	0.0165	0.0331	0.0400	0.0300	0.0380	0.0134	0.0074	0.0348
	p	0.0180	**0.0000**	**0.0000**	**0.0000**	**0.0000**	**0.0000**	**0.0000**	0.0360	**0.0000**	**0.0000**	**0.0000**	**0.0000**	**0.0000**	**0.0000**	0.0090	0.0180	**0.0000**
TPOX	Fst	-0.0001	0.0116	0.0036	-0.0037	0.0260	0.0024	0.0027	-0.0015	-0.0020	0.0053	0.0031	0.0007	0.0037	0.0014	0.0057	0.0014	0.0044
	p	0.3153	0.0541	0.1622	0.6577	0.0090	0.2252	0.2523	0.5045	0.4505	0.0721	0.1532	0.2613	0.1261	0.2432	0.1351	0.2072	0.1261
VWA	Fst	0.0236	0.0200	0.0390	0.0203	0.0134	0.0204	0.0222	0.0163	0.0092	0.0165	0.0255	0.0225	0.0240	0.0347	0.0116	0.0117	0.0111
	p	**0.0000**	**0.0000**	**0.0000**	**0.0000**	0.0090	**0.0000**	**0.0000**	0.0270	0.0901	**0.0000**	**0.0000**	**0.0000**	**0.0000**	**0.0000**	0.0090	0.0090	0.0541

Continue Supplementary Table S12

Locus		Bangladeshi	Indian	Japanese	Korea-1	Korea-2	Native-American	West-Mexican-Mestizo	Monterrey-Mexican-Mestizo	South-Portuguese	Southern-Portugal-Angolan	Poland	Northern-Italy	Australian-Self-Declared-Aboriginal	Australian-Asian	Australian-Caucasian	Australian-Pure-Aboriginal	New-Zealand-East-Polynesian
CSF1PO	Fst	0.0104	0.0113	0.0148	0.0084	0.0078	0.0097	0.0004	0.0006	-0.0030	-0.0007	-0.0012	-0.0011	0.0070	0.0034	-0.0001	0.0083	0.0055
	p	0.0901	0.0451	0.0090	0.0541	0.0360	0.0451	0.3874	0.3604	0.7207	0.3964	0.4595	0.4144	0.0991	0.1892	0.3874	0.0360	0.1802
D1S1656	Fst	0.0142	0.0114	0.0073	0.0055	0.0045	0.0203	0.0100	0.0185	0.0306	0.0394	0.0277	0.0208	0.0107	0.0087	0.0215	0.0190	0.0344
	p	**0.0000**	0.0090	0.0090	0.0811	0.0631	**0.0000**	0.0180	**0.0000**	**0.0000**	**0.0000**	**0.0000**	**0.0000**	**0.0000**	0.0090	**0.0000**	**0.0000**	**0.0000**
D2S441	Fst	-0.0020	0.0002	0.0062	0.0172	0.0107	0.0452	0.0179	0.0318	0.0351	0.0367	0.0276	0.0393	0.0220	0.0158	0.0264	0.0688	0.1230
	p	0.5315	0.3063	0.0811	**0.0000**	0.0090	**0.0000**	0.0090	**0.0000**	**0.0000**	**0.0000**	0.0090	**0.0000**	**0.0000**	0.0090	**0.0000**	**0.0000**	**0.0000**
D2S1338	Fst	0.0003	-0.0001	-0.0019	0.0005	-0.0001	0.0339	0.0101	0.0482	0.0210	0.0219	0.0408	0.0220	0.0026	0.0055	0.0095	0.0147	0.0212
	p	0.4595	0.4865	0.7297	0.3423	0.4775	**0.0000**	0.0090	**0.0000**	**0.0000**	**0.0000**	**0.0000**	**0.0000**	0.2162	0.0360	**0.0000**	**0.0000**	**0.0000**
D3S1358	Fst	0.0001	-0.0001	0.0073	0.0080	0.0123	0.0612	0.0115	0.0167	0.0022	0.0029	0.0166	0.0011	0.0012	0.0111	0.0074	0.0006	0.0012
	p	0.3694	0.4054	0.0811	0.0901	0.0180	**0.0000**	0.0451	**0.0000**	0.2072	0.2793	**0.0000**	0.3063	0.4144	0.0360	0.0541	0.3243	0.3333
D5S818	Fst	-0.0039	-0.0036	0.0026	0.0034	0.0023	0.0514	0.0098	0.0180	0.0023	0.0213	0.0039	0.0063	0.0019	0.0065	0.0026	0.0120	0.0382
	p	0.7478	0.7478	0.2342	0.1892	0.2162	**0.0000**	0.0811	0.0180	0.1982	0.0180	0.2072	0.1261	0.2432	0.0451	0.2162	**0.0000**	**0.0000**
D7S820	Fst	-0.0012	-0.0023	0.0106	0.0086	0.0126	0.0177	0.0119	0.0143	0.0046	0.0035	0.0098	0.0000	0.0005	0.0022	0.0019	0.0163	0.0151
	p	0.5315	0.6216	0.0270	**0.0000**	0.0180	**0.0000**	0.0180	0.0090	0.1712	0.2523	0.0090	0.3964	0.4144	0.2793	0.1802	**0.0000**	0.0270
D8S1179	Fst	0.0232	0.0205	0.0038	0.0038	0.0042	0.0059	0.0022	-0.0006	-0.0050	0.0262	-0.0006	-0.0036	0.0021	0.0105	-0.0027	0.0114	0.0151
	p	**0.0000**	**0.0000**	0.0721	0.1081	0.0451	0.0721	0.2432	0.5225	0.9910	**0.0000**	0.5496	0.8378	0.2432	**0.0000**	0.6847	0.0180	**0.0000**
D10S1248	Fst	0.0566	0.0357	0.0080	0.0040	0.0054	0.0506	0.0185	0.0277	0.0083	0.0276	0.0287	0.0020	0.0226	0.0022	0.0096	0.0315	0.0354
	p	**0.0000**	**0.0000**	0.0270	0.1171	0.0991	**0.0000**	**0.0000**	**0.0000**	0.0811	**0.0000**	**0.0000**	0.1622	**0.0000**	0.2432	0.0270	**0.0000**	**0.0000**
D12S391	Fst	0.0115	0.0087	0.0134	0.0075	0.0125	0.0111	0.0007	0.0056	0.0131	0.0225	0.0133	0.0147	0.0101	0.0048	0.0097	0.0110	0.0067
	p	**0.0000**	0.0090	**0.0000**	0.0270	**0.0000**	0.0270	0.3874	0.0631	**0.0000**	**0.0000**	**0.0000**	**0.0000**	**0.0000**	0.0901	0.0090	**0.0000**	0.0451

D13S317	Fst	0.0174	0.0091	0.0177	0.0158	0.0184	0.0149	0.0044	0.0038	0.0059	0.0301	0.0091	0.0051	0.0208	0.0166	0.0052	0.0425	0.0088
	p	0.0090	0.0270	**0.0000**	**0.0000**	0.0090	**0.0000**	0.1351	0.1261	0.0360	**0.0000**	0.0090	0.0991	**0.0000**	**0.0000**	0.1171	**0.0000**	0.0180
D16S539	Fst	0.0172	0.0186	0.0499	0.0197	0.0234	0.0342	0.0281	0.0215	0.0024	0.0320	0.0068	0.0063	0.0224	0.0098	0.0063	0.0355	0.0465
	p	0.0180	0.0090	**0.0000**	0.0090	**0.0000**	**0.0000**	**0.0000**	**0.0000**	0.2162	0.0090	0.1171	0.0991	**0.0000**	**0.0000**	0.0631	**0.0000**	**0.0000**
D18S51	Fst	0.0043	0.0018	0.0007	-0.0001	0.0006	0.0135	0.0052	0.0051	0.0043	0.0381	0.0111	0.0019	0.0029	0.0006	0.0059	0.0097	0.0465
	p	0.1351	0.2793	0.3333	0.4775	0.3694	**0.0000**	0.1081	0.0090	0.0451	**0.0000**	0.0090	0.2252	0.0991	0.4054	0.0721	**0.0000**	**0.0000**
D19S433	Fst	-0.0031	-0.0017	0.0070	0.0003	0.0001	0.0073	0.0096	0.0114	0.0090	0.0119	0.0193	0.0074	0.0225	-0.0009	0.0155	0.0366	0.0100
	p	0.7207	0.5856	0.0451	0.3153	0.3874	0.0180	0.0270	0.0180	0.0180	0.0090	**0.0000**	0.0541	**0.0000**	0.5405	**0.0000**	**0.0000**	0.0360
D21S11	Fst	0.0229	0.0226	0.0041	0.0088	0.0072	0.1769	0.0088	0.0059	0.0162	0.0420	0.0184	0.0089	0.0096	-0.0006	0.0139	0.0103	0.0114
	p	**0.0000**	**0.0000**	0.1441	0.0451	0.0270	**0.0000**	0.0180	0.0811	**0.0000**	**0.0000**	**0.0000**	0.0360	0.0180	0.5315	**0.0000**	0.0090	0.0451
D22S1045	Fst	0.0328	0.0467	0.0126	0.0130	0.0122	0.0772	0.0574	0.0780	0.0247	0.0209	0.0186	0.0347	0.0694	0.0142	0.0343	0.1439	0.0760
	p	**0.0000**	**0.0000**	0.0090	**0.0000**	0.0180	**0.0000**	**0.0000**	**0.0000**	**0.0000**	0.0090	**0.0000**	**0.0000**	**0.0000**	**0.0000**	**0.0000**	**0.0000**	**0.0000**
FGA	Fst	0.0004	0.0057	0.0069	0.0039	0.0043	0.0199	0.0009	0.1174	0.0147	-0.0002	0.0250	0.0092	0.0067	0.0055	0.0195	0.0074	0.0263
	p	0.3694	0.0631	0.0180	0.0901	0.0991	**0.0000**	0.2973	**0.0000**	**0.0000**	0.4144	**0.0000**	0.0090	0.0360	0.0541	**0.0000**	0.0180	**0.0000**
TH01	Fst	0.0330	0.0107	0.0186	0.0190	0.0202	0.1858	0.0593	0.0814	0.0237	0.0864	0.0322	0.0450	0.0609	0.0158	0.0440	0.0671	0.0756
	p	**0.0000**	**0.0000**	**0.0000**	0.0090	**0.0000**	**0.0000**	**0.0000**	**0.0000**	**0.0000**	**0.0000**	**0.0000**	**0.0000**	**0.0000**	**0.0000**	**0.0000**	**0.0000**	**0.0000**
TPOX	Fst	0.0136	0.0165	-0.0016	-0.0018	-0.0006	0.0032	0.0030	0.0053	0.0086	0.0227	0.0171	0.0071	0.0832	0.0080	0.0089	0.1362	0.0074
	p	0.0451	0.0090	0.5135	0.4685	0.3874	0.1712	0.2072	0.1351	0.0631	**0.0000**	0.0090	0.0811	**0.0000**	0.0901	0.0631	**0.0000**	0.1081
VWA	Fst	0.0120	0.0137	0.0183	0.0128	0.0183	0.0488	0.0306	0.0334	0.0126	0.0216	0.0103	0.0114	0.0094	0.0220	0.0107	0.0145	0.0186
	p	0.0360	0.0090	**0.0000**	**0.0000**	**0.0000**	**0.0000**	**0.0000**	**0.0000**	**0.0000**	0.0090	0.0090	0.0541	0.0180	**0.0000**	**0.0000**	**0.0000**	**0.0000**

Continue Supplementary Table S12

Locus		New-Zealand-West-Polynesian	New-Zealand-Caucasian	North-Brazil	Northeast-Brazil	Central-West-Brazil	Southeast-Brazil	South-Brazil	Argentina-Chubut	Paraguay	Ecuador	South-Africa-Afrikaaner	South-Africa-Asian-Indian	South-Africa-Cape-Coloured	South-Africa-amaXhosa	South-Africa-amaZulu
CSF1PO	Fst	0.0009	-0.0018	0.0031	0.0001	-0.0028	-0.0016	0.0002	-0.0013	-0.0005	0.0022	-0.0058	0.0181	0.0002	-0.0003	0.0048
	p	0.4054	0.4685	0.2252	0.3784	0.5676	0.4414	0.3604	0.4865	0.3694	0.2072	0.6577	0.0090	0.2703	0.3333	0.1622
D1S1656	Fst	0.0240	0.0329	0.0288	0.0198	0.0317	0.0266	0.0177	0.0177	0.0226	0.0268	0.0233	0.0143	0.0094	0.0391	0.0372
	p	**0.0000**	**0.0000**	**0.0000**	**0.0000**	**0.0000**	**0.0000**	**0.0000**	**0.0000**	**0.0000**	**0.0000**	**0.0000**	**0.0000**	0.0270	**0.0000**	**0.0000**
D2S441	Fst	0.0691	0.0199	0.0373	0.0281	0.0222	0.0210	0.0148	0.0160	0.0241	0.0841	0.0209	-0.0037	0.0069	0.0467	0.0650
	p	**0.0000**	**0.0000**	**0.0000**	**0.0000**	**0.0000**	**0.0000**	**0.0000**	0.0090	**0.0000**	**0.0000**	**0.0000**	0.6667	0.1441	**0.0000**	**0.0000**
D2S1338	Fst	0.0153	0.0054	0.0084	0.0124	0.0077	0.0155	0.1049	0.0103	0.0059	0.0161	0.0090	-0.0030	0.0085	0.0410	0.0354
	p	**0.0000**	0.1081	0.0270	0.0090	0.0090	**0.0000**	**0.0000**	0.0090	0.0631	**0.0000**	0.0360	0.6216	0.0180	**0.0000**	**0.0000**
D3S1358	Fst	0.0059	0.0086	0.0044	0.0045	0.0025	-0.0033	0.0020	0.0116	0.0023	0.0245	0.0121	-0.0056	-0.0009	0.0299	0.0193
	p	0.1712	0.1081	0.1892	0.1622	0.1982	0.7297	0.2162	0.0270	0.2432	**0.0000**	0.0721	0.8469	0.4595	0.0090	0.0451
D5S818	Fst	0.0337	-0.0008	0.0051	0.0029	0.0019	0.0044	0.0023	0.0248	0.0083	0.0390	-0.0005	-0.0079	-0.0020	0.0198	0.0133
	p	0.0090	0.4505	0.0991	0.2883	0.3243	0.1351	0.2162	**0.0000**	0.0451	**0.0000**	0.3514	0.9189	0.4865	0.0360	0.0180
D7S820	Fst	0.0220	0.0026	0.0012	-0.0003	-0.0025	0.0078	0.0034	0.0195	0.0097	0.0279	0.0080	-0.0041	-0.0032	0.0151	0.0031
	p	**0.0000**	0.2703	0.3153	0.4054	0.6757	0.0991	0.1982	**0.0000**	0.0270	**0.0000**	0.1261	0.7207	0.5586	0.0270	0.2523
D8S1179	Fst	0.0057	-0.0053	-0.0004	0.0012	-0.0028	-0.0015	-0.0040	-0.0045	-0.0018	0.0007	-0.0067	0.0248	0.0009	0.0283	0.0249
	p	0.1171	0.8559	0.4324	0.2793	0.6937	0.5135	0.8469	0.9640	0.5856	0.3423	0.9099	**0.0000**	0.3694	**0.0000**	0.0090
D10S1248	Fst	0.0093	0.0003	0.0091	0.0065	0.0125	0.0023	0.0146	0.0316	0.0280	0.0350	0.0117	0.0419	0.0138	0.0102	0.0162
	p	0.0631	0.3874	0.0451	0.0991	0.0090	0.2072	**0.0000**	**0.0000**	**0.0000**	**0.0000**	0.0541	**0.0000**	0.0541	0.0811	0.0180
D12S391	Fst	0.0026	0.0132	0.0006	0.0069	0.0043	0.0076	0.0050	0.0050	0.0126	0.0215	0.0102	0.0161	0.0039	0.0056	0.0158
	p	0.2342	0.0090	0.3063	0.0270	0.0811	0.0451	0.0991	0.0270	**0.0000**	**0.0000**	0.0541	**0.0000**	0.1712	0.1081	0.0270

D13S317	Fst	0.0005	0.0108	0.0045	0.0154	0.0027	0.0126	-0.0002	0.0053	0.0035	0.0205	0.0107	0.0060	0.0324	0.0481	0.0476
	p	0.2973	0.0270	0.1622	**0.0000**	0.2072	0.0270	0.4324	0.0901	0.1802	**0.0000**	0.0721	0.0541	**0.0000**	**0.0000**	**0.0000**
D16S539	Fst	0.0347	0.0106	0.0048	0.0009	0.0027	0.0057	0.0013	0.0034	0.0037	0.0276	0.0013	0.0109	0.0086	0.0427	0.0311
	p	**0.0000**	0.0541	0.1532	0.2973	0.2342	0.0631	0.2883	0.1802	0.2252	**0.0000**	0.3063	0.0811	0.0901	**0.0000**	**0.0000**
D18S51	Fst	0.0367	0.0057	0.0061	0.0073	0.0074	0.0053	0.0078	0.0091	0.0092	0.0132	0.0092	0.0024	0.0073	0.0438	0.0474
	p	**0.0000**	0.0631	0.0901	0.0991	0.0180	0.0721	0.0270	**0.0000**	0.0180	**0.0000**	0.0901	0.3514	0.0631	**0.0000**	**0.0000**
D19S433	Fst	0.0169	0.0215	0.0066	0.0023	0.0040	0.0060	0.0091	0.0132	0.0084	0.0197	0.0163	-0.0036	-0.0031	0.0095	0.0132
	p	0.0180	**0.0000**	0.0631	0.1802	0.1441	0.0901	0.0360	**0.0000**	0.0180	**0.0000**	0.0090	0.7297	0.7387	0.0360	**0.0000**
D21S11	Fst	0.0391	0.0158	0.0093	0.0177	0.0245	0.0199	0.0111	0.0107	0.0110	0.0104	0.0170	0.0080	0.0121	0.0753	0.0829
	p	**0.0000**	0.0090	0.0721	**0.0000**	**0.0000**	**0.0000**	**0.0000**	0.0090	**0.0000**	0.0090	0.0180	0.0721	0.0360	**0.0000**	**0.0000**
D22S1045	Fst	0.1221	0.0200	0.0363	0.0166	0.0175	0.0238	0.0269	0.0945	0.0520	0.0781	0.0158	0.0225	0.0170	0.0172	0.0121
	p	**0.0000**	0.0090	0.0090	0.0090	0.0180	**0.0000**	**0.0000**	**0.0000**	**0.0000**	**0.0000**	0.0270	**0.0000**	0.0451	0.0090	0.0360
FGA	Fst	0.0159	0.0175	0.0080	0.0049	0.0064	0.0142	0.0066	0.0108	0.0120	0.0120	0.0126	0.0020	0.0067	-0.0012	0.0036
	p	0.0180	**0.0000**	0.0180	0.1351	0.0631	0.0270	0.0451	0.0090	0.0090	0.0090	0.0180	0.2523	0.0451	0.5405	0.1802
TH01	Fst	0.1053	0.0393	0.0543	0.0307	0.0567	0.0547	0.0533	0.0713	0.0522	0.1102	0.0601	0.0061	0.0373	0.1264	0.1041
	p	**0.0000**	**0.0000**	**0.0000**	**0.0000**	**0.0000**	**0.0000**	**0.0000**	**0.0000**	**0.0000**	**0.0000**	**0.0000**	0.1712	**0.0000**	**0.0000**	**0.0000**
TPOX	Fst	0.0271	0.0029	-0.0042	0.0035	0.0021	-0.0004	-0.0026	-0.0005	-0.0015	0.0043	0.0086	0.0247	0.0158	0.0327	0.0270
	p	0.0090	0.2162	0.6847	0.2703	0.2703	0.3423	0.5496	0.5135	0.4595	0.1351	0.1532	0.0180	0.0451	**0.0000**	**0.0000**
VWA	Fst	0.0058	0.0063	0.0262	0.0245	0.0220	0.0116	0.0103	0.0315	0.0243	0.0614	0.0083	0.0102	0.0147	0.0358	0.0266
	p	0.1532	0.1171	0.0090	**0.0000**	**0.0000**	**0.0000**	0.0451	**0.0000**	**0.0000**	**0.0000**	0.0721	0.0901	0.0451	**0.0000**	**0.0000**

The bold font means significant.

Supplementary Table S13. The Nei's standard genetic distances between our three studied populations and 47 previously published populations from worldwide.

Population	P1	P2	P3	P4	P5	P6	P7	P8	P9	P10	P11	P12	P13	P14	P15	P16	P17
[P1]Ningxia-Wuzhong-Hui																	
[P2]Sichuan-Chengdu-Tibetan	0.0259																
[P3]Xinjiang-Kumul-Uyghur	0.0474	0.0691															
[P4]Hainan-Han	0.0146	0.0359	0.0588														
[P5]Sichuan-Liangshan-Yi	0.0176	0.0181	0.0519	0.0255													
[P6]Sichuan-Liangshan-Tibetan	0.0219	0.0217	0.0599	0.0345	0.0204												
[P7]Sihcuan-Han-1	0.0119	0.0255	0.0560	0.0139	0.0148	0.0285											
[P8]Sihcuan-Han-2	0.0123	0.0289	0.0558	0.0152	0.0180	0.0306	0.0022										
[P9]Xinjiang-Uyghur-1	0.0421	0.0696	0.0549	0.0526	0.0530	0.0606	0.0482	0.0484									
[P10]Tibet-Tibetan	0.0242	0.0193	0.0623	0.0379	0.0201	0.0257	0.0269	0.0302	0.0599								
[P11]Xinjiang-Uyghur-2	0.0283	0.0466	0.0360	0.0362	0.0359	0.0432	0.0297	0.0312	0.0230	0.0414							
[P12]Guangdong-Han	0.0092	0.0228	0.0508	0.0073	0.0135	0.0247	0.0054	0.0083	0.0446	0.0240	0.0269						
[P13]Central-Chinese-Han	0.0072	0.0198	0.0486	0.0115	0.0107	0.0216	0.0040	0.0053	0.0478	0.0226	0.0292	0.0034					
[P14]Xiamen-Han	0.0104	0.0257	0.0544	0.0073	0.0184	0.0274	0.0076	0.0092	0.0502	0.0271	0.0332	0.0035	0.0052				
[P15]Guizhou-Han	0.0104	0.0254	0.0576	0.0085	0.0160	0.0300	0.0061	0.0094	0.0505	0.0258	0.0328	0.0025	0.0052	0.0041			
[P16]Xinjiang-Kazakh	0.0281	0.0430	0.0326	0.0389	0.0324	0.0385	0.0300	0.0307	0.0261	0.0368	0.0088	0.0278	0.0278	0.0342	0.0340		
[P17]Xinjiang-Uyghur-3	0.0248	0.0438	0.0324	0.0329	0.0322	0.0396	0.0287	0.0296	0.0222	0.0388	0.0035	0.0251	0.0265	0.0304	0.0307	0.0067	
[P18]Ulaanbaatar-Mongolian	0.0262	0.0434	0.0450	0.0370	0.0280	0.0346	0.0253	0.0258	0.0382	0.0366	0.0206	0.0257	0.0233	0.0317	0.0313	0.0114	0.0189
[P19]Bangladeshi	0.0612	0.0809	0.0738	0.0686	0.0682	0.0824	0.0697	0.0725	0.0559	0.0691	0.0377	0.0615	0.0674	0.0674	0.0641	0.0446	0.0316
[P20]Indian	0.0637	0.0878	0.0669	0.0722	0.0743	0.0878	0.0733	0.0776	0.0537	0.0794	0.0355	0.0658	0.0722	0.0729	0.0686	0.0447	0.0295
[P21]Japanese	0.0270	0.0441	0.0601	0.0294	0.0330	0.0484	0.0246	0.0273	0.0570	0.0475	0.0402	0.0204	0.0220	0.0232	0.0249	0.0367	0.0364
[P22]Korea-1	0.0122	0.0303	0.0469	0.0171	0.0192	0.0312	0.0126	0.0145	0.0490	0.0319	0.0321	0.0084	0.0081	0.0100	0.0107	0.0306	0.0281
[P23]Korea-2	0.0110	0.0270	0.0486	0.0176	0.0184	0.0286	0.0131	0.0157	0.0515	0.0292	0.0335	0.0085	0.0079	0.0099	0.0104	0.0311	0.0285
[P24]Native-American	0.1540	0.1531	0.1402	0.1638	0.1434	0.1640	0.1434	0.1560	0.1309	0.1451	0.1001	0.1390	0.1486	0.1550	0.1480	0.1032	0.1045
[P25]West-Mexican-Mestizo	0.0995	0.1108	0.0806	0.1072	0.1020	0.1140	0.0956	0.1009	0.0762	0.1063	0.0504	0.0930	0.1001	0.1034	0.1012	0.0566	0.0502
[P26]Monterrey-Mexican-Mestizo	0.1479	0.1553	0.1176	0.1520	0.1479	0.1595	0.1440	0.1507	0.1234	0.1456	0.0919	0.1383	0.1488	0.1481	0.1471	0.0964	0.0948
[P27]South-Portuguese	0.0887	0.1193	0.0653	0.0949	0.1049	0.1141	0.0973	0.0969	0.0547	0.1082	0.0407	0.0895	0.0981	0.0954	0.0980	0.0488	0.0371
[P28]Southern-Portugal-Angolan	0.1333	0.1634	0.1412	0.1405	0.1500	0.1497	0.1480	0.1487	0.1047	0.1460	0.0968	0.1403	0.1454	0.1454	0.1528	0.1081	0.0974
[P29]Poland	0.1161	0.1579	0.0885	0.1280	0.1430	0.1465	0.1323	0.1305	0.0724	0.1476	0.0615	0.1236	0.1327	0.1314	0.1342	0.0693	0.0566
[P30]Northern-Italy	0.1178	0.1466	0.0867	0.1276	0.1360	0.1420	0.1276	0.1274	0.0764	0.1381	0.0619	0.1228	0.1296	0.1303	0.1311	0.0712	0.0590

[P31]Australian-Self-Declared-	0.1004	0.1290	0.0975	0.1145	0.1149	0.1206	0.1110	0.1174	0.0758	0.1143	0.0664	0.1031	0.1139	0.1171	0.1116	0.0731	0.0605
[P32]Australian-Asian	0.0172	0.0336	0.0528	0.0129	0.0258	0.0345	0.0133	0.0172	0.0389	0.0359	0.0226	0.0073	0.0146	0.0109	0.0092	0.0261	0.0212
[P33]Australian-Caucasian	0.1001	0.1304	0.0731	0.1075	0.1158	0.1233	0.1072	0.1081	0.0556	0.1179	0.0436	0.1012	0.1100	0.1111	0.1107	0.0505	0.0397
[P34]Australian-Pure-Aboriginal	0.1362	0.1651	0.1460	0.1528	0.1522	0.1548	0.1485	0.1605	0.1255	0.1550	0.1161	0.1407	0.1509	0.1558	0.1479	0.1237	0.1107
[P35]New-Zealand-East-Polynesian	0.1730	0.1849	0.1710	0.1805	0.1679	0.1971	0.1595	0.1650	0.1623	0.1715	0.1489	0.1661	0.1714	0.1722	0.1696	0.1557	0.1502
[P36]New-Zealand-West-Polynesian	0.1417	0.1555	0.1550	0.1405	0.1396	0.1581	0.1241	0.1335	0.1305	0.1446	0.1132	0.1325	0.1381	0.1426	0.1386	0.1270	0.1164
[P37]New-Zealand-Caucasian	0.1411	0.1684	0.1158	0.1522	0.1530	0.1639	0.1465	0.1452	0.0934	0.1551	0.0899	0.1433	0.1526	0.1476	0.1513	0.0950	0.0839
[P38]North-Brazil	0.0989	0.1147	0.0761	0.1113	0.1043	0.1211	0.0971	0.1004	0.0716	0.1087	0.0495	0.0955	0.1025	0.1059	0.1071	0.0520	0.0484
[P39]Northeast-Brazil	0.0823	0.1117	0.0658	0.0898	0.0952	0.1042	0.0876	0.0880	0.0505	0.0971	0.0345	0.0845	0.0906	0.0907	0.0936	0.0412	0.0327
[P40]Central-West-Brazil	0.0870	0.1135	0.0696	0.0940	0.0971	0.1085	0.0916	0.0947	0.0543	0.1004	0.0378	0.0851	0.0936	0.0939	0.0940	0.0428	0.0351
[P41]Southeast-Brazil	0.0994	0.1249	0.0725	0.1041	0.1098	0.1153	0.1043	0.1070	0.0621	0.1102	0.0444	0.0985	0.1071	0.1064	0.1097	0.0509	0.0418
[P42]South-Brazil	0.1118	0.1397	0.0869	0.1200	0.1231	0.1352	0.1138	0.1157	0.0773	0.1276	0.0600	0.1105	0.1180	0.1201	0.1188	0.0677	0.0558
[P43]Argentina-Chubut	0.1098	0.1316	0.0874	0.1198	0.1192	0.1343	0.1087	0.1138	0.0846	0.1234	0.0539	0.1050	0.1141	0.1158	0.1138	0.0630	0.0555
[P44]Paraguay	0.0972	0.1219	0.0734	0.1065	0.1057	0.1247	0.0973	0.1008	0.0697	0.1085	0.0495	0.0941	0.1022	0.1029	0.1039	0.0522	0.0459
[P45]Ecuador	0.1455	0.1497	0.1285	0.1533	0.1432	0.1573	0.1353	0.1434	0.1292	0.1464	0.0953	0.1354	0.1447	0.1456	0.1460	0.0954	0.0965
[P46]South-Afica-Afrikaaner	0.1177	0.1436	0.0822	0.1254	0.1308	0.1315	0.1252	0.1253	0.0694	0.1321	0.0571	0.1204	0.1272	0.1275	0.1300	0.0628	0.0531
[P47]South-Afica-Asian-Indian	0.0748	0.0975	0.0778	0.0827	0.0804	0.0995	0.0838	0.0872	0.0704	0.0889	0.0509	0.0769	0.0813	0.0843	0.0796	0.0591	0.0419
[P48]South-Afica-Cape-Coloured	0.1043	0.1290	0.1049	0.1138	0.1199	0.1170	0.1177	0.1205	0.0840	0.1160	0.0757	0.1134	0.1204	0.1191	0.1198	0.0822	0.0713
[P49]South-Afica-amaXhosa	0.1913	0.2240	0.2140	0.1947	0.1932	0.2019	0.2049	0.2076	0.1643	0.1973	0.1634	0.1944	0.1995	0.1993	0.2058	0.1703	0.1622
[P50]South-Afica-amaZulu	0.1683	0.1872	0.1815	0.1774	0.1704	0.1752	0.1811	0.1829	0.1333	0.1734	0.1260	0.1749	0.1785	0.1798	0.1859	0.1395	0.1268

Continue Supplementary Table S13

Population	P18	P19	P20	P21	P22	P23	P24	P25	P26	P27	P28	P29	P30	P31	P32	P33	P34
[P19]Bangladeshi	0.0641																
[P20]Indian	0.0691	0.0130															
[P21]Japanese	0.0397	0.0748	0.0701														
[P22]Korea-1	0.0268	0.0707	0.0700	0.0134													
[P23]Korea-2	0.0276	0.0690	0.0689	0.0136	0.0032												
[P24]Native-American	0.1169	0.1505	0.1313	0.1254	0.1399	0.1344											
[P25]West-Mexican-Mestizo	0.0788	0.0991	0.0797	0.0864	0.0922	0.0910	0.0366										
[P26]Monterrey-Mexican-Mestizo	0.1240	0.1372	0.1213	0.1252	0.1400	0.1370	0.0400	0.0303									
[P27]South-Portuguese	0.0849	0.0770	0.0665	0.0943	0.0922	0.0939	0.1359	0.0538	0.0901								
[P28]Southern-Portugal-Angolan	0.1237	0.1239	0.1216	0.1455	0.1422	0.1432	0.2070	0.1408	0.1826	0.1002							

[P29]Poland	0.1111	0.0956	0.0846	0.1273	0.1266	0.1290	0.1670	0.0767	0.1176	0.0163	0.1267						
[P30]Northern-Italy	0.1084	0.0912	0.0790	0.1270	0.1235	0.1253	0.1733	0.0787	0.1226	0.0229	0.1196	0.0350					
[P31]Australian-Self-Declared-	0.1029	0.0718	0.0533	0.1040	0.1053	0.1061	0.1286	0.0756	0.1100	0.0612	0.1325	0.0747	0.0794				
[P32]Australian-Asian	0.0313	0.0585	0.0565	0.0292	0.0180	0.0185	0.1317	0.0785	0.1221	0.0695	0.1310	0.0981	0.1000	0.0818			
[P33]Australian-Caucasian	0.0897	0.0790	0.0645	0.1040	0.1044	0.1052	0.1293	0.0518	0.0902	0.0077	0.1106	0.0149	0.0250	0.0529	0.0771		
[P34]Australian-Pure-Aboriginal	0.1457	0.1106	0.0850	0.1378	0.1388	0.1399	0.1655	0.1281	0.1590	0.1350	0.1830	0.1551	0.1493	0.0246	0.1214	0.1274	
[P35]New-Zealand-East-Polynesian	0.1701	0.1923	0.1756	0.1686	0.1665	0.1669	0.2325	0.1858	0.2269	0.1695	0.1818	0.2078	0.1803	0.2192	0.1506	0.1684	0.2776
[P36]New-Zealand-West-Polynesian	0.1439	0.1408	0.1242	0.1392	0.1454	0.1473	0.1686	0.1342	0.1608	0.1465	0.1427	0.1788	0.1540	0.1350	0.1164	0.1412	0.1685
[P37]New-Zealand-Caucasian	0.1367	0.1355	0.1209	0.1475	0.1440	0.1444	0.1947	0.1022	0.1391	0.0357	0.1420	0.0457	0.0554	0.1029	0.1153	0.0364	0.1871
[P38]North-Brazil	0.0817	0.0847	0.0722	0.0861	0.0975	0.0953	0.0636	0.0226	0.0440	0.0373	0.1132	0.0603	0.0658	0.0676	0.0823	0.0393	0.1267
[P39]Northeast-Brazil	0.0698	0.0713	0.0614	0.0870	0.0885	0.0877	0.1096	0.0428	0.0734	0.0149	0.0625	0.0341	0.0380	0.0647	0.0680	0.0199	0.1322
[P40]Central-West-Brazil	0.0714	0.0683	0.0607	0.0848	0.0879	0.0862	0.0906	0.0357	0.0689	0.0172	0.0694	0.0392	0.0420	0.0562	0.0683	0.0218	0.1206
[P41]Southeast-Brazil	0.0817	0.0808	0.0685	0.0955	0.0998	0.1004	0.1153	0.0461	0.0752	0.0150	0.0678	0.0341	0.0333	0.0615	0.0788	0.0191	0.1270
[P42]South-Brazil	0.0982	0.0898	0.0751	0.1093	0.1125	0.1137	0.1190	0.0565	0.1011	0.0394	0.1128	0.0415	0.0616	0.0741	0.0922	0.0366	0.1418
[P43]Argentina-Chubut	0.0919	0.0938	0.0746	0.0932	0.1026	0.0996	0.0442	0.0167	0.0320	0.0474	0.1496	0.0685	0.0717	0.0732	0.0906	0.0449	0.1289
[P44]Paraguay	0.0795	0.0845	0.0681	0.0799	0.0935	0.0909	0.0537	0.0175	0.0392	0.0350	0.1303	0.0550	0.0618	0.0643	0.0804	0.0350	0.1222
[P45]Ecuador	0.1171	0.1457	0.1259	0.1180	0.1380	0.1309	0.0272	0.0251	0.0312	0.1065	0.2082	0.1347	0.1427	0.1197	0.1236	0.1036	0.1673
[P46]South-Afica-Afrikaaner	0.1019	0.0963	0.0853	0.1220	0.1229	0.1238	0.1480	0.0649	0.1011	0.0151	0.1199	0.0215	0.0312	0.0784	0.0958	0.0111	0.1635
[P47]South-Afica-Asian-Indian	0.0794	0.0226	0.0184	0.0833	0.0787	0.0781	0.1533	0.0999	0.1415	0.0803	0.1377	0.1056	0.0957	0.0693	0.0718	0.0821	0.0984
[P48]South-Afica-Cape-Coloured	0.1022	0.0898	0.0792	0.1268	0.1167	0.1172	0.1789	0.1128	0.1588	0.0864	0.0939	0.1072	0.1040	0.1021	0.0999	0.0903	0.1524
[P49]South-Afica-amaXhosa	0.1787	0.1743	0.1782	0.2031	0.2007	0.1993	0.2603	0.2022	0.2409	0.1808	0.0820	0.2157	0.1996	0.2020	0.1901	0.1882	0.2534
[P50]South-Afica-amaZulu	0.1535	0.1420	0.1409	0.1824	0.1781	0.1794	0.2356	0.1776	0.2168	0.1383	0.0275	0.1666	0.1548	0.1615	0.1675	0.1453	0.2109

Continue Supplementary Table S13

Population	P35	P36	P37	P38	P39	P40	P41	P42	P43	P44	P45	P46	P47	P48	P49	P50
[P36]New-Zealand-West-Polynesian	0.0941															
[P37]New-Zealand-Caucasian	0.1505	0.1741														
[P38]North-Brazil	0.1708	0.1263	0.0809													
[P39]Northeast-Brazil	0.1531	0.1154	0.0537	0.0251												
[P40]Central-West-Brazil	0.1607	0.1173	0.0564	0.0198	0.0089											
[P41]Southeast-Brazil	0.1569	0.1197	0.0501	0.0273	0.0093	0.0100										
[P42]South-Brazil	0.1810	0.1428	0.0731	0.0473	0.0387	0.0380	0.0396									
[P43]Argentina-Chubut	0.1839	0.1390	0.0970	0.0204	0.0411	0.0330	0.0439	0.0564								

[P44]Paraguay	0.1801	0.1365	0.0784	0.0116	0.0273	0.0222	0.0295	0.0450	0.0128						
[P45]Ecuador	0.2279	0.1662	0.1549	0.0348	0.0834	0.0703	0.0888	0.1012	0.0279	0.0313					
[P46]South-Afica-Afrikaaner	0.1783	0.1580	0.0409	0.0487	0.0267	0.0302	0.0243	0.0438	0.0583	0.0445	0.1174				
[P47]South-Afica-Asian-Indian	0.2005	0.1458	0.1327	0.0878	0.0782	0.0737	0.0856	0.0912	0.0952	0.0832	0.1464	0.1011			
[P48]South-Afica-Cape-Coloured	0.1874	0.1435	0.1309	0.1014	0.0705	0.0767	0.0786	0.1020	0.1190	0.1047	0.1741	0.0954	0.0912		
[P49]South-Afica-amaXhosa	0.2362	0.1899	0.2306	0.1805	0.1376	0.1393	0.1434	0.1842	0.2098	0.1979	0.2636	0.1915	0.1985	0.1571	
[P50]South-Afica-amaZulu	0.1943	0.1628	0.1719	0.1446	0.0955	0.1030	0.1017	0.1427	0.1826	0.1684	0.2467	0.1473	0.1585	0.1188	0.0727

Supplementary Table S14. The Nei's standard genetic distances between our three studied populations and 14 previously published populations of China.

Population	[P1]	[P2]	[P3]	[P4]	[P5]	[P6]	[P7]	[P8]	[P9]	[P10]	[P11]	[P12]	[P13]	[P14]	[P15]	[P16]	[P17]
[P1]Ningxia-Wuzhong-Hui																	
[P2]Sichuan-Chengdu-Tibetan	0.0259																
[P3]Xinjiang-Kumul-Uyghur	0.0474	0.0691															
[P4]Hainan-Han	0.0146	0.0359	0.0588														
[P5]Sichuan-Liangshan-Yi	0.0176	0.0181	0.0519	0.0255													
[P6]Sichuan-Liangshan-Tibetan	0.0219	0.0217	0.0599	0.0345	0.0204												
[P7]Sihcuan-Han-1	0.0119	0.0255	0.0560	0.0139	0.0148	0.0285											
[P8]Sihcuan-Han-2	0.0123	0.0289	0.0558	0.0152	0.0180	0.0306	0.0022										
[P9]Xinjiang-Uyghur-1	0.0421	0.0696	0.0549	0.0526	0.0530	0.0606	0.0482	0.0484									
[P10]Tibet-Tibetan	0.0242	0.0193	0.0623	0.0379	0.0201	0.0257	0.0269	0.0302	0.0599								
[P11]Xinjiang-Uyghur-2	0.0283	0.0466	0.0360	0.0362	0.0359	0.0432	0.0297	0.0312	0.0230	0.0414							
[P12]Guangdong-Han	0.0092	0.0228	0.0508	0.0073	0.0135	0.0247	0.0054	0.0083	0.0446	0.0240	0.0269						
[P13]Central-Chinese-Han	0.0072	0.0198	0.0486	0.0115	0.0107	0.0216	0.0040	0.0053	0.0478	0.0226	0.0292	0.0034					
[P14]Xiamen-Han	0.0104	0.0257	0.0544	0.0073	0.0184	0.0274	0.0076	0.0092	0.0502	0.0271	0.0332	0.0035	0.0052				
[P15]Guizhou-Han	0.0104	0.0254	0.0576	0.0085	0.0160	0.0300	0.0061	0.0094	0.0505	0.0258	0.0328	0.0025	0.0052	0.0041			
[P16]Xinjiang-Kazakh	0.0281	0.0430	0.0326	0.0389	0.0324	0.0385	0.0300	0.0307	0.0261	0.0368	0.0088	0.0278	0.0278	0.0342	0.0340		
[P17]Xinjiang-Uyghur-3	0.0248	0.0438	0.0324	0.0329	0.0322	0.0396	0.0287	0.0296	0.0222	0.0388	0.0035	0.0251	0.0265	0.0304	0.0307	0.0067	

Supplementary Table S15. Proportion of membership of each pre-defined population in each of the three clusters (structure analysis based on 23 STRs included in the Huaxia Platinum System). The population full names are displayed in **Supplementary Table S2**.

Population abbreviation	Population size	Cluster 1	Cluster 2	Cluster 3
NWH*	183	0.3619	0.3717	0.2665
SCT*	200	0.3006	0.4903	0.2091
SLT	198	0.3173	0.4655	0.2172
TT	100	0.3158	0.4400	0.2442
SLY	177	0.3347	0.4280	0.2373
XKU*	110	0.3109	0.2693	0.4198
XJU-1	100	0.3178	0.2628	0.4194
XJU-2	1218	0.3069	0.2418	0.4513
HNH	193	0.3685	0.3903	0.2412
SCH-1	309	0.3844	0.3786	0.2370
SCH-2	202	0.3794	0.3772	0.2435

* stand for our investigated populations.
